# Glycomic Characterization of Induced Pluripotent Stem Cells Derived from a Patient Suffering from Phosphomannomutase 2 Congenital Disorder of Glycosylation (PMM2-CDG)* [Fn FN2]

**DOI:** 10.1074/mcp.M115.054122

**Published:** 2016-01-19

**Authors:** Christina T. Thiesler, Samanta Cajic, Dirk Hoffmann, Christian Thiel, Laura van Diepen, René Hennig, Malte Sgodda, Robert Weiβmann, Udo Reichl, Doris Steinemann, Ulf Diekmann, Nicolas M. B. Huber, Astrid Oberbeck, Tobias Cantz, Andreas W. Kuss, Christian Körner, Axel Schambach, Erdmann Rapp, Falk F. R. Buettner

**Affiliations:** From the ‡REBIRTH-Cluster of Excellence, Hannover Medical School, 30625 Hannover, Germany;; §Institute for Cellular Chemistry, Hannover Medical School, 30625 Hannover, Germany;; ¶Max Planck Institute for Dynamics of Complex Technical Systems, 39106 Magdeburg, Germany;; ‖Institute of Experimental Hematology, Hannover Medical School, 30625 Hannover, Germany;; **Center for Child and Adolescent Medicine, Department Kinderheilkunde I, 69120 Heidelberg, Germany;; ‡‡Department of Human Genetics, University Medicine Greifswald and Interfaculty Institute for Genetics and Functional Genomics, Ernst-Moritz-Arndt University, 17475 Greifswald, Germany;; §§glyXera GmbH, 39120 Magdeburg, Germany;; ¶¶Translational Hepatology and Stem Cell Biology, Dept. of Gastroenterology, Hepatology, and Endocrinology, Hannover Medical School, 30625 Hannover, Germany;; ‖‖Institute of Human Genetics, Hannover Medical School, 30625 Hannover, Germany;; *^a^*Institute of Clinical Biochemistry, Hannover Medical School, Hannover, Germany

## Abstract

PMM2-CDG, formerly known as congenital disorder of glycosylation-Ia (CDG-Ia), is caused by mutations in the gene encoding phosphomannomutase 2 (*PMM2*). This disease is the most frequent form of inherited CDG-diseases affecting protein N-glycosylation in human. PMM2-CDG is a multisystemic disease with severe psychomotor and mental retardation. In order to study the pathophysiology of PMM2-CDG in a human cell culture model, we generated induced pluripotent stem cells (iPSCs) from fibroblasts of a PMM2-CDG-patient (PMM2-iPSCs). Expression of pluripotency factors and *in vitro* differentiation into cell types of the three germ layers was unaffected in the analyzed clone PMM2-iPSC-C3 compared with nondiseased human pluripotent stem cells (hPSCs), revealing no broader influence of the PMM2 mutation on pluripotency in cell culture. Analysis of gene expression by deep-sequencing did not show obvious differences in the transcriptome between PMM2-iPSC-C3 and nondiseased hPSCs. By multiplexed capillary gel electrophoresis coupled to laser induced fluorescence detection (xCGE-LIF) we could show that PMM2-iPSC-C3 exhibit the common hPSC N-glycosylation pattern with high-mannose-type N-glycans as the predominant species. However, phosphomannomutase activity of PMM2-iPSC-C3 was 27% compared with control hPSCs and lectin staining revealed an overall reduced protein glycosylation. In addition, quantitative assessment of N-glycosylation by xCGE-LIF showed an up to 40% reduction of high-mannose-type N-glycans in PMM2-iPSC-C3, which was in concordance to the observed reduction of the Glc3Man9GlcNAc2 lipid-linked oligosaccharide compared with control hPSCs. Thus we could model the PMM2-CDG disease phenotype of hypoglycosylation with patient derived iPSCs *in vitro*. Knock-down of *PMM2* by shRNA in PMM2-iPSC-C3 led to a residual activity of 5% and to a further reduction of the level of N-glycosylation. Taken together we have developed human stem cell-based cell culture models with stepwise reduced levels of N-glycosylation now enabling to study the role of N-glycosylation during early human development.

Congenital disorder of glycosylation type Ia (CDG-Ia^1^, recently named PMM2-CDG) is an inherited autosomal recessive rare disease caused by mutations in the phosphomannomutase 2 (*PMM2*) gene (1) and affected more than 800 patients worldwide in 2009 (calculated frequency 1:20.000 to 1:50.000 births) (2, 3). The majority of compound heterozygous PMM2-CDG patients carry the mutations 422 G>A and 357 C>A in the *PMM2* gene leading to Arg^141^His and Phe^119^Leu mutations in the respective proteins (4). These patients show a broad clinical picture affecting nearly all organ systems and an overall mortality of 20% during childhood. Typical pediatric symptoms include failure to thrive, hypotonia, hepatic dysfunction, and dysmorphic features like inverted nipples and subcutaneous fat pads. After infancy patients show psychomotor and mental retardation (5, 6). PMM2 catalyzes the conversion of mannose-6-phosphate (Man-6-P) to mannose-1-phosphate (Man-1-P), which is essential to synthesize GDP-mannose (GDP-Man). The activated donor sugar GDP-Man is required for the synthesis of the lipid-linked oligosaccharide (LLO), the glycan donor for N-glycosylation (7). A marked reduction in PMM2 activity has been shown in fibroblasts and leukocytes of PMM2-CDG patients and was identified as the cause of PMM2-CDG (1, 8).

Fibroblasts derived from PMM2-CDG patients that were cultured under low glucose conditions reflected the expected phenotype and accumulated precursors of the LLO, which are poor substrates for the oligosaccharyltransferase complex. Raising the glucose level to physiological conditions can lead to abundant synthesis of LLO without hypoglycosylation. However, high levels of Man-6-P in PMM-CDG function as activator for cleavage of LLO leading to futile cycling of the LLO pathway (9). In order to study PMM2-CDG under more complex physiological conditions, different animal models have been developed. Morpholino-mediated knock-down of *pmm2* in *Xenopus laevis* embryos caused underglycosylation and developmental defects (10). Similarily, in a morpholino-based PMM2-CDG model of zebrafish the developmental abnormalities seen in PMM2-CDG patients could be partially imitated. Furthermore, N-glycosylation and LLO levels were reduced in morphant zebrafish embryos and Man-6-P was proven to induce cleavage of the LLO (11). Targeted disruption of the *Pmm2* gene in mice resulted in embryonic lethality around day 3.5 suggesting an essential role of PMM2 for early embryonic development (12). Similar results were observed for a homozygous Arg^137^His (corresponding to Arg^141^His in humans) mutation of Pmm2 in mice (13). In a hypomorphic Pmm2 mouse model Arg^137^His and Phe^118^Leu mutations, resembling the predominant human mutations, embryos survived to embryonic day 9.5 and had a reduced staining with the lectin wheat germ agglutinin (WGA) that binds to sialic acids and N-acetylglucosamine residues. Interestingly, embryonic lethality of mutant embryos could be rescued by feeding mannose to pregnant mice and the offspring survived beyond weaning. Under mannose supplementation histological examination revealed no abnormal morphology of mutant embryos on embryonic day 16.5 and they displayed a normal WGA staining (13). These findings emphasize the particular meaning of glycosylation for embryonic development, which can be studied *in vitro* in a less complex environment by using embryonic stem cells (ESCs) (14). The seminal work of Takahashi and Yamanaka, showing the generation of induced pluripotent stem cells (iPSCs) from differentiated adult fibroblasts (15) has enabled the generation of patient-specific iPSCs for disease modeling. IPSCs and their differentiated progeny present an important tool for the study of molecular mechanisms and cellular pathways resulting in disease manifestation. Furthermore, disease-specific iPSCs display an unlimited cell source for the generation of differentiated progeny as well as for the development and testing of new therapeutic compounds *in vitro* (16, 17).

In order to take advantage of these possibilities, we generated iPSCs as a disease model for PMM2-CDG by reprogramming fibroblasts from a PMM2-CDG patient (PMM2-iPSCs). The PMM2-iPSCs were characterized in depth by glycomics and we could clearly show reduction of high-mannose-type N-glycans as early as on the stem cell level. The development and characterization of iPSC models for PMM2-CDG offer the possibility to dissect the effects of (aberrant) glycosylation in early human development in future.

## EXPERIMENTAL PROCEDURES

### 

#### 

##### Fibroblast Culture

Fibroblasts (PMM2-CDG patient derived fibroblasts were obtained from the NIGMS Human Genetic Cell Repository at the Coriell Institute for Medical Research (GM20942), Camden, NJ; human embryonic fibroblasts (huEFs) for use as feeder cells: ATCC, Manassas, VA (CCD919); mouse embryonic fibroblasts (MEFs) for use as feeder cells: EmbryoMax® PMEF-P3, strain CF-1, Millipore, Billerica, MA) were cultured on 0.1% [w/v] gelatin (Sigma-Aldrich, St. Louis, MO) coated culture flasks in high glucose DMEM medium, supplemented with 10% [v/v] FCS, 1% [v/v] MEM Nonessential Amino Acids, and 0.05% [v/v] GlutaMax^TM^ (all from Live Technologies, Carlsbad, CA) at 37 °C, 5% CO_2_ and 85% relative humidity. Fibroblasts intended for use as feeder cells were irradiated (3000 cGy).

##### Stem Cell Culture

Human embryonic stem cells (ES03: ES Cell International, National Stem Cell Bank Wisconsin, WI; H9: WA09 Wisconsin Alumni Research Foundation (Wicell Research Institute, Inc.) Madison, WI) were cultured on irradiated huEFs. Human induced pluripotent stem cells (CBiPSC2: human cord blood derived induced pluripotent stem cell clone 2, reprogrammed from cord blood derived endothelial cells (18); HD2-iPSC: healthy donor derived iPSC clone 2, reprogrammed from human skin fibroblasts (not published); PMM2-iPSC: reprogrammed from PMM2-CDG fibroblasts (this study)) were cultured on irradiated MEFs. hESCs and hiPSCs were cultured on feeder cells in stem cell medium consisting of KnockOut^TM^ DMEM supplemented with 20% [v/v] KnockOut^TM^ Serum Replacement, 1% [v/v] MEM Nonessential Amino Acids, 0.05% [v/v] GlutaMax^TM^, 0.1 mm 2-mercaptoethanol (all reagents from Life Technologies) and 50 ng/ml (ES03), 10 ng/ml (CBiPSC2) or 40 ng/ml (HD2-iPSC and PMM2-iPSC) basic fibroblast growth factor (bFGF, Institute of Technical Chemistry, Leibniz University Hannover, Hannover, Germany) at 37 °C, 5% CO_2_ and 85% relative humidity. For passaging, cells were washed with PBS and incubated for 5 min with 0.2% [w/v] Collagenase IV (Life Technologies) at 37 °C and subsequently transferred to new feeder cells.

For analyses, cells were cultivated at least three passages under feeder-free conditions on Matrigel^TM^ (1:30–1:60) (BD Biosciences, Bedford, MA) in mTeSR^TM^1 medium (StemCell Technologies, Grenoble, France) or murine embryonic fibroblast-conditioned medium (MEF-CM) as described previously (19). For passaging, cells were briefly washed with PBS and incubated for 7 min with 1 mg/ml [w/v] Dispase (StemCell Technologies) at 37 °C and reseeded onto new Matrigel^TM^ coated flasks.

##### Reprogramming of PMM2 Patient-derived Fibroblasts

For reprogramming, the all in one self-inactivating lentiviral vector pRRL.PPT.SF.Oct4co.Klf4co.Sox2co.cMyc.idTom.pre carrying the four reprogramming factors (RFs) OCT4, KLF4, SOX2 and cMYC was used. RFs were codon-optimized for the human system and coexpressed with the fluorescent protein dTomato to directly monitor RF-expression. Expression is driven by the spleen focus-forming virus (SFFV) promoter that is rapidly silenced upon epigenetic cell remodeling during the reprogramming process (20). Human skin fibroblasts from a male PMM2-CDG patient (NIGMS Human Genetic Cell Repository) were cultured in fibroblast medium (low-glucose DMEM, 15% FCS, 2 mm l-glutamine, 100 units/ml penicillin, 100 μg/ml streptomycin (all from PAA, Pasching, Austria), 0.1 mm MEM Nonessential Amino Acids (Life Technologies) and 100 μm 2-mercaptoethanol (Sigma-Aldrich)) and transduced with the lentiviral reprogramming vector in the presence of 4 μg/ml protamine sulfate. Cells were first spin-inoculated at 860 × *g* for one hour and afterward incubated overnight. Medium was replaced daily by fibroblast medium supplemented with 50 μg/ml 2-phospho-vitamin C (pVitC) (Sigma-Aldrich) and 2 mm valpronic acid (VPA, Ergenyl®, Sanofi-Aventis, Frankfurt, Germany) for the following 7 days. At day eight, cells were trypsinized and transferred to irradiated (3000 cGy) C3H MEF cells (Charles River, Sulzfeld, Germany) and cultured in iPSC medium (DMEM/F12 with GlutaMax^TM^, 20% KnockOut^TM^ Serum Replacement, 2 mm l-glutamine, 1% MEM Nonessential Amino Acids, 100 μm 2-mercaptoethanol (all Life Technologies), 20 ng/ml bFGF, 100 units/ml penicillin, 100 μg/ml streptomycin, 50 μg/ml pVitC and 2 mm VPA) and medium was changed daily. First colonies were manually picked at around day 20 and expanded on C3H MEFs in iPSC medium.

##### Knock-down of PMM2 by RNAi

Stable expression of shRNAs from an optimized mir-30 backbone (21) was achieved upon lentiviral transduction. PMM2-iPSC-C3 were transduced with VSV-G pseudotyped particles expressing three different *PMM2*-specific shRNAs or Renilla shRNA as control (supplemental Table S1) at a multiplicity of infection (MOI) of 10. For transduction, PMM2-iPSC-C3 were cultured on Matrigel^TM^ in mTeSR^TM^1 incubated for 2 h with 10 μm Rock Inhibitor (RI) and detached by incubation with Trypsin/EDTA (TE, Life Technologies). Trypsin was inactivated by addition of fibroblast medium. Subsequently, cells were centrifuged at 500 × *g* for 5 min at room temperature. Fibroblast medium was removed and cells were resuspended in mTeSR^TM^1 medium supplemented with 10 μm RI. 3 × 10^4^ cells were transduced in single cell suspension (100 μl) with lentiviral particles in the presence of 4 μg/ml protamine sulfate for 1 h at 37 °C and 5% CO_2_. Afterward, virus-cells suspensions were transferred to Matrigel^TM^ coated 21 mm wells containing 500 μl fresh mTeSR^TM^1 with 10 μm RI. Cells were cultured for two passages. Stable GFP-positive cells (expressing the shRNA) were sorted by flow cytometry.

##### Differentiation of PMM2-iPSC-C3 into the Three Germ Layers

Differentiation into hepatic cells was performed according to a modified monolayer based protocol developed by Sgodda and coworkers (22). In brief, single cells were seeded at a density of 5 × 10^4^ cells/cm^2^ on Matrigel^TM^-coated plates in mTeSR^TM^1 medium containing 10 μm RI. After reaching confluence, differentiation was initiated by culture for 3 days in RPMI medium supplemented with 0,5 mg/ml BSA, 5% KnockOut^TM^ Serum Replacement, 100 ng/ml Activin A (Peprotech, Hamburg, Germany) and 10 μm Ly294002 (Merck, Darmstadt, Germany). At the first day of differentiation 3 μm CHIR 99021 (Institute of Technical Chemistry) was added. In the following 4 days, cells were differentiated into definitive endoderm in hepatocyte culture medium (HCM) without EGF supplemented with 2% fatty acid free BSA (Lonza, Cologne, Germany), 10 μm SB431542 (Sigma Aldrich), 30 ng/ml FGF4 (Peprotech) and 20 ng/ml BMP2 (Peprotech). Only on day four 0.5 μg/ml sFRP-5 (R&D Systems, Heidelberg, Germany) was added. At day 9, medium was exchanged to HCM without EGF supplemented with 2% fatty acid free BSA (Lonza), 20 ng/ml HGF (Peprotech) and 10 μm SB431542. Under these conditions, cells were differentiated into hepatic cells for further 4 days and maintained till day 15.

Cardiomyogenic differentiation was performed by small molecule-based modulation of WNT signaling (23). Briefly, hPSCs cultivated on Matrigel^TM^ in mTeSR^TM^1 were detached with 0.5 mm EDTA in PBS and seeded as single cells on Matrigel^TM^ in mTeSR^TM^1 supplemented with 5 μm RI (Institute of Technical Chemistry) at a density of 1 × 10^5^ to 2 × 10^5^ cells/cm^2^. The medium was exchanged against mTeSR^TM^1 without RI, on the following day. After reaching confluence mTeSR^TM^1 was exchanged against RPMI (Life Technologies) supplemented with B-27® without insulin (B-27-I) (Life Technologies) and 6 μm CHIR-99021 (Selleckchem, Houston, TX). The next day, medium was exchanged against RPMI/B27-I. On day four RPMI/B-27-I media was supplemented with 5 μm IWP4 (Stemgent, Cambridge, MA). At day 6, IWP4 was removed and at day 8, media were exchanged against RPMI/B-27® with insulin (B-27+I, Life Technologies).

Using STEMdiff^TM^ Neural Induction Medium (StemCell Technologies), hPSCs were differentiated into neural progenitor cells (NPCs) according to the manufacturer's protocol. Briefly, hPSCs were seeded as single cells on Matrigel^TM^ in STEMdiff^TM^ Neural Induction Medium supplemented with 10 μm RI at a density of 2.0 × 10^5^ to 2.5 × 10^5^ cells/cm^2^. This medium (without RI) was exchanged daily for 10 days.

##### Immunofluorescence (IF) Microscopy

Cells were washed with PBS and fixed with 4% [w/v] paraformaldehyde in PBS (AppliChem, Darmstadt, Germany) for 30 min followed by blocking with 2% [w/v] BSA in PBS (blocking buffer) for 1 h. All antibodies were incubated for 1 h in blocking buffer and cells were washed with PBS between each incubation step. After labeling of cell surface epitopes, cells were again fixed for 10 min with 4% [w/v] paraformaldehyde in PBS. For labeling of intracellular epitopes, cells were permeabilized and blocked in the presence of 0.1% Triton X-100. Antibodies and dilutions used for IF are listed in supplemental Table S2 and S3. Nuclei were counterstained with Hoechst32258 (AppliChem). Cells were embedded into Dako fluorescence mounting medium (Agilent Technologies, Santa Clara, CA) and analyzed with a Zeiss Axiovert 200 m microscope equipped with an AxioCam MRm digital camera and Zen 2012 (blue edition) software (Zeiss, Oberkochen, Germany).

##### Array Comparative Genomic Hybridization (Array-CGH)

Array-CGH was performed using the Agilent Human Genome Microarray format 4 × 180k (Agilent Technologies), a 60-mer oligonucleotide based microarray with median overall probe spacing of about 13 KB. Labeling and hybridization of genomic DNA was performed according to the manufacturer's protocol (Agilent). Briefly, 1 μg of DNA, each was labeled by random priming using the Agilent Genomic DNA Labeling Kit Plus. After labeling sample DNA with Cy3-dUTP and reference DNA, consisting of a pool of 10 control samples with Cy5-dUTP, products were purified by Amicon Ultra 30k filters (Millipore). Sample and reference DNA were pooled and mixed with 50 μg human Cot-1 DNA (to later prevent unspecific hybridization), Agilent 10× Blocking Agent, and Agilent 2X Hybridization Buffer. This mixture was hybridized to Agilent's 4 × 180k Human Genome CGH microarray at 65 °C with 20 rpm rotation for 24 h. Washing steps were performed according to the manufacturer's protocol (Agilent Technologies). Microarray slides were scanned immediately using a microarray scanner at a resolution of 2 μm (G2505B, Agilent Technologies). For image analysis, default CGH settings of Feature Extraction Software (Agilent Technologies) were applied. Output files from Feature Extraction were subsequently imported into Agilent's CGH data analysis software, Genomic-Workbench. The Aberration Algorithm ADM2 was applied and set to 5.0 and Aberration Filters were set to: centralization threshold 6.0, at least 3 probes with mean log2 ratio of −/+0.2 leading to a real resolution of around 120 KB. Copy number variations were reported according to the Database of Genomic Variants (http://dgv.tcag.ca/).

##### Total RNA Preparation

Cells were harvested in TRIzol® (Life Technologies). RNA extraction was performed adding 200 μl chloroform (VWR International, Radnor, PA) per ml TRIzol® followed by short stirring and centrifuging for 15 min at 12,000 × *g* and room temperature. Ethanol (70% [v/v], (VWR International)), was added in a 1:1 ratio to the upper, aqueous phase. The following filtration steps were performed according to the NucleoSpin® RNA II Kit (Macherey-Nagel, Düren, Germany). Purified RNA was stored at −80 °C.

##### cDNA Synthesis

Residual genomic DNA was digested with RQ1 RNase-free DNase (Promega, Madison, WI) for 30 min at 37 °C. The reaction was stopped applying RQ1 DNase stop solution (Promega) for 10 min at 70 °C. cDNA was synthesized with random hexamer primers (Life Technologies) and RevertAid^TM^ Premium Reverse Transcriptase (Thermo Scientific, Waltham, MA) for 10 min at 25 °C, 50 min at 42 °C and 10 min at 70 °C and stored at −20 °C.

##### Quantitative Real-time PCR (qPCR)

qPCR reactions were performed in a total volume of 20 μl with a volume of cDNA corresponding to 20 or 25 ng of purified RNA, 9.5 pmol primer pair, 4 nmol dNTPs (dATP, dCTP, dTTP, dGTP), 45 nmol MgCl_2_, 10% [v/v] Maxima Hot Start TaqPCR buffer, 0.5 U Maxima Hot Start TaqDNA Polymerase (all Thermo Scientific), 1% [v/v] ROX reference dye and 8% [v/v] of SYBR Green Nucleic Acid Stain diluted 1:10000 (both from Life Technologies). Reactions were performed with a 7500 Fast Real-time PCR System in sealed 96-well optical reaction plates (both from Life Technologies) for 40 cycles (15 s at 90 °C, 60 s at 60 °C) after 10 min denaturation at 95 °C. Amplicon sizes were evaluated by melting-curve analysis (95 °C, 15 s, 60 °C 60 s, 95 °C, 30 s, 60 °C 15 s). Relative expression of target genes was determined by normalization to the respective housekeeping genes. Ct-values were determined automatically by the 7500 Software v2.0.5. (Relative expression of target genes was calculated by determination of 2^−ΔΔCt^-values.) Used primer pairs (all from Sigma-Aldrich) are listed in supplemental Table S4.

##### Determination of Genomic Vector Integrations

Genomic DNA from PMM2-iPSCs was isolated using the QIAamp DNA blood kit according to the manufacturer's instructions (Qiagen, Hilden, Germany). Vector copy numbers of PMM2-iPSCs were determined by quantitative PCR using Taqman PCR kit and Step One Plus real-time PCR system (ABI, Darmstadt, Germany). Primers and Taqman probes for the vector-specific PRE and the PTBP2 intron and the reference sequence plasmid were used as described previously (24).

##### Verification of Mutation Arg^141^His/Phe^119^Leu in PMM2-iPSCs

RNA purification and cDNA synthesis of CBiPSC2 and PMM2-iPSCs was performed as described above. A DNA region of 371 bp, covering the two respective point mutations [357C>A] and [422G>A] leading to the amino acid changes Phe^119^Leu and Arg^141^His, was amplified by PCR using primers 5′-CCAGAAAATGGCTTGGTAGC-3′ and 5′-CAGTATCTCTTGTCCCATCC-3′. PCR products were separated by 1% agarose gel electrophoresis, bands were cut out, purified with the NucleoSpin® Gel and PCR Clean-up Kit (Machery-Nagel) according to the manufacturer's instructions and sequenced (GATC Biotech, Konstanz, Germany). Data analysis was performed with the SaqMan^TM^-tool of the Lasergene 7® software (DNASTAR, Inc., Madison, WI).

##### Deep-Sequencing

hPSCs cultivated on Matrigel^TM^ in MEF-CM were harvested, lysed in TRIzol® (Life Technologies) and total RNA was extracted applying NucleoSpin® RNA II Kit (Machery-Nagel). To remove rRNA the Ribo-Zero^TM^ Kit (Epicenter, Madison, WI) was used. Subsequently, 10 μg of each total RNA sample was mixed with Ambion® ERCC Spike-in Control Mixes (Life Technologies). For sequencing, RNA was prepared according to the SOLiD® Total RNA-Seq Kit (Life Technologies). Briefly, rRNA-free RNA was hydrolyzed into small fragments followed by a phosphorylation step. After adaptor-ligation and hybridization, cDNA was reverse transcribed from the purified RNA-fragments. cDNA fragments of correct length were purified by Agencourt AMPure XP bead purification (Beckman Coulters Genomics, Danvers, MA) and amplified for 12 PCR cycles in a Biometra T3 Thermocycler using barcoded primers. Size distribution and concentration of fragments was determined with an Agilent 2100 Bioanalyzer and its provided chemistry (Agilent Technologies). cDNA fragments were pooled in equimolar ratios and diluted to a concentration of 500 pm (61 pg/μl). In compliance with the E80 scale protocol provided by the manufacturer (Life Technologies), 50 μl of cDNA mixtures were spiked with a fresh oil emulsion, P1 beads, P1 and P2 chemicals in a SOLiD EZ Bead Emulsifier. Amplification of cDNA was conducted in the E80-modus of a SOLiD EZ Bead Amplifier (Life Technologies). Beads, carrying the amplified template DNA, were purified on a SOLiD EZ Bead Enricher (Life Technologies). The purified beads were put upside down onto a SOLiD 6-lane Flowchip for 1 h at 37 °C. The DNA on the Flowchip was sequenced in read length of 75 bases in a 5500xl SOLiD System.

Procession and read mapping of the run was carried out with the Lifescope software analysis tool (Life Technologies) and reference genome GRCh37/hg19. Statistical analysis was performed with R (25). Normalization and differential expression calculations were carried out using the R packages EDASeq (26) and DESeq (27), respectively.

##### Determination of Phosphomannomutase Activity

Phosphomannomutase activity was determined as the turnover rate of Man-6-P to Man-1,6-P in the presence of cofactor Glu-1,6-P. Twelve micrograms protein of cell homogenates were mixed with 1 μl [2-^3^H]Man-6-P in a buffer containing 50 mm Tris pH 7.5, 2 mm MgCl_2_ and 2 mm Glu-1,6-P. Reaction mixtures were incubated for 30 min at 37 °C followed by 3 min at 95 °C to stop the reaction. Afterward, samples were put on ice and mixed with 1 μl 5% Orange G. Samples were loaded onto 3 mm Whatman paper soaked with 80 mm pyridine buffer (adjusted to pH 5.5 with glacial acetic acid). [2-^3^H]Man-6-P and [2-^3^H]Man-1,6-P were separated via high voltage electrophoresis at 65 V/cm for 45 min. After drying, paper sheets were analyzed using a flatbed scanner (Trancemaster 20, Berthold) (28).

##### Analysis of Lipid-linked Oligosaccharides, Incorporation of [2-^3^H]Mannose and [^35^S]Methionine into Newly Synthesized Glycoproteins and Dol-P-Man

Analysis of lipid-linked oligosaccharides (LLO) was performed with 1.2 × 10^6^ of control and patient-derived cells. For metabolic labeling 125 μCi [2-^3^H]mannose or 25 μCi [^35^S]methionine were applied. Aliquots were used for protein determination and further procedure was conducted as described previously (28, 29).

##### Lectin/Western Blots

Cells were lysed in 20 mm Tris (Merck), 130 mm NaCl (VWR International), 1% [v/v] Nonidet P-40 (Roche, Basel, Switzerland) supplemented with the protease inhibitors Aprotinin (0.8 μm, (Roth, Karlsruhe, Germany)), Bestatin (50 μm, (Sigma)), Leupeptin (20 μm, (Roche)), Pepstatin A (10 μm, (Sigma)) and PMSF (100 μm, (Roche)), followed by two rounds of sonication for 1 min. In between, lysates were incubated on ice for 10 min. Cell debris were separated by centrifugation for 2 min at 13,000 × *g* and 4 °C. Protein concentrations of the supernatant were determined with the Pierce^©^660 nm Protein Assay Kit (Thermo Scientific). Lysates were dissolved in Laemmli buffer (35 mm Tris pH 6.8 (Merck), 2.8% [w/v] SDS (Roth), 7% [v/v] glycerol (VWR International) and 0.005% [w/v] bromphenol blue (AppliChem)) and incubated for 5 min at 95 °C followed by 5 min on ice. Proteins were separated by 10% SDS-PAGE and blotted in a semi-dry mode with blotting buffer (48 mm Tris pH 9, 39 mm glycine) onto nitrocellulose membranes (Whatman^TM^, Protran BA85, GE Healthcare, Little Chalfont, UK) for 1 h at 2 mA/cm^2^. Blots were developed according to the DIG Glycan Differentiation Kit (Roche). Briefly, after incubating in blocking solution (10x Blocking Reagent and PBS) for 30 min at RT or overnight at 4 °C, membranes were washed twice with TBS for 10 min and once with lectin binding buffer (1× TBS, 1 mm MgCl_2_, 1 mm MnCl_2_, 1 mm CaCl_2_, pH 7.5). Staining with DIG-labeled GNA (supplemental Table S5) was performed for 1 h at RT followed by 3 times TBS-washing for 10 min. For development with alkaline phosphatase, membranes were incubated with anti-DIG-AP 1:1000 (Roche) in TBS for 1 h followed by TBS and a short AP-Buffer (100 mm Tris pH 9.5, 100 mm NaCl) wash. Lectin binding was visualized by incubation with 0.02% [v/v] BCIP (Roth)/NBT (Sigma) in AP-Buffer.

For Western blot analysis, membranes were blocked with Odyssey™ Blocking Buffer (LI-COR, Lincoln, NE) for 1 h at room temperature and incubated with the respective primary antibodies (supplemental Table S2) overnight at 4 °C. Membranes were washed three times with PBS-T (PBS/0.1% TWEEN 20 (Sigma-Aldrich)) followed by one-hour incubation with the respective secondary LI-COR antibody (supplemental Table S3). Membranes were washed again 3 times with PBS-T and analyzed with an ODYSSEY®-infrared imaging system and the corresponding software Odyssey v3.0 (LI-COR).

##### Flow Cytometry

For lectin flow cytometry, cells were washed twice with PBS and incubated for 5 min at 37 °C with 0.5 mm EDTA in PBS to make a single cell suspension. For each staining, single cells were incubated with the respective lectin (supplemental Table S5) in flow buffer (0.2% BSA in PBS) for 30 min at room temperature. As a specificity control for GNA, 1 m d-mannose (Sigma Aldrich) was added to the flow buffer before lectin supplementation. After washing twice with buffer, cells were analyzed using a CyFlow ML flow cytometer (Partec GmbH, Münster, Germany) and Flowing Software 2 (Perttu Terho, Turku Centre for Biotechnology, Turku, Finland).

##### Multiplexed Capillary Gel Electrophoresis with Laser Induced Fluorescence Detection (xCGE-LIF)

Cells were lysed in RIPA-Buffer (50 mm Tris pH 8, 150 mm NaCl, 1% [v/v] Nonidet P-40 (Roche), 0.5% [w/v] natrium desoxycholate (Sigma Aldrich) and 1% [w/v] SDS (Serva, Heidelberg, Germany)), supplemented with HALT protease inhibitor (Thermo Scientific) by pipetting up and down a few times followed by freezing at −80 °C overnight. Thawed cells were sonicated two times for 1 min followed by centrifuging for 15 min at 13,000 × *g* and 4 °C. Proteins were precipitated with a 4-fold excess volume of ice cold acetone at −20 °C overnight. After centrifugation, protein pellets were fully dissolved in 2% [w/v] SDS in 1x PBS (both Applichem). Further sample preparation for xCGE-LIF-based glycoanalysis was performed as previously described (30) with minor modifications. Briefly, to remove contaminants, 200 μg total protein of each sample was loaded onto an SDS-PAGE gel and run only for a short time (8 min). The resulting band of the still unseparated Coomassie-stained proteins was excised and then treated with peptide-N-glycosidase F (PNGase F from *Elizabethkingia meningoseptica*, BioReagent, Sigma Aldrich) to release the attached N-glycans. The enzymatically released N-glycans were extracted from the gel pieces with Milli-Q water. To obtain N-glycosylation patterns by xCGE-LIF, released N-glycans were first labeled with 8-aminopyrene-1,3,6-trisulfonic acid (APTS, Sigma Aldrich) and excess label was then removed using polyacrylamide based stationary phase in hydrophilic-interaction-chromatography (HILIC) mode. Subsequently, purified fluorescently labeled N-glycans were separated by multiplexed capillary gel electrophoresis (xCGE) and monitored via laser induced fluorescence (LIF) detection. xCGE-LIF data processing and normalization of migration times to an internal standard were performed with glyXtool™ software (glyXera, Magdeburg, Germany). The generated N-glycan “fingerprints” (normalized electropherograms) were used for annotation of N-glycan peaks via migration time matching with the in-house N-glycan database (glyXtool v. 5.3.0). All database-matched N-glycan structures were confirmed by extensive exoglycosidase digests and subsequent repeated analysis by xCGE-LIF. Following specific enzymes were used: α(2–3) sialidase (Sialidase S, recombinant from *Streptococcus pneumoniae*, expressed in *Escherichia coli*; Prozyme, Hayward, CA), α(2–3,6,8) sialidase (Sialidase A, recombinant from *Arthrobacter ureafaciens*, expressed in *Escherichia coli*; Prozyme), α(1–3,4) fucosidase (recombinant from *Xanthomonas*; QA-Bio), α(1–2) fucosidase (recombinant from *Xanthomonas manihotis*, expressed in *Escherichia coli*; New England Biolabs, Ipswich, MA), α(1–2,3,4,6) fucosidase (from bovine kidney; Prozyme), β(1–3) galactosidase (recombinant from *Xanthomonas manihotis*, expressed in *Escherichia coli*; New England Biolabs), β(1–4) galactosidase (recombinant from *Bacteroides fragilis*, expressed in *Escherichia coli*; New England Biolabs), β(1–4,6) galactosidase (from Jack bean; Prozyme), β(1–2,3,4,6)-N-acetylglucosaminidase (recombinant from *Xanthomonas manihotis*, expressed in *Escherichia coli*; New England Biolabs), α(1–2,3,6) mannosidase (from Jack bean; Prozyme). Exoglycosidase digestions were carried out at 37 °C in buffers and under conditions recommended by the suppliers of the enzymes. Samples were purified by HILIC solid phase extraction (SPE) prior to xCGE-LIF analysis. Specific activity and possible side activity was carefully tested for each exoglycosidase enzyme used. For inter-sample quantitative comparison, 1 μg of bovine asialofetuin was spiked to each sample as an internal standard prior to short-time SDS-PAGE separation. One asialofetuin derived N-glycan peak inside the xCGE-LIF electropherogram was used for quantitative normalization of sample peak intensities.

##### Statistics

Statistical analysis was carried out with GraphPad Prism software. Unless stated otherwise, mean value, standard error of the mean (S.E.) and number of replicates (n) are displayed. One asterisk denotes *p* < 0.05, two asterisks denote *p* < 0.01 and three asterisks denote *p* < 0.001.

## RESULTS

### 

#### 

##### Generation and Characterization of PMM2-iPSCs

**Fig. 1.**
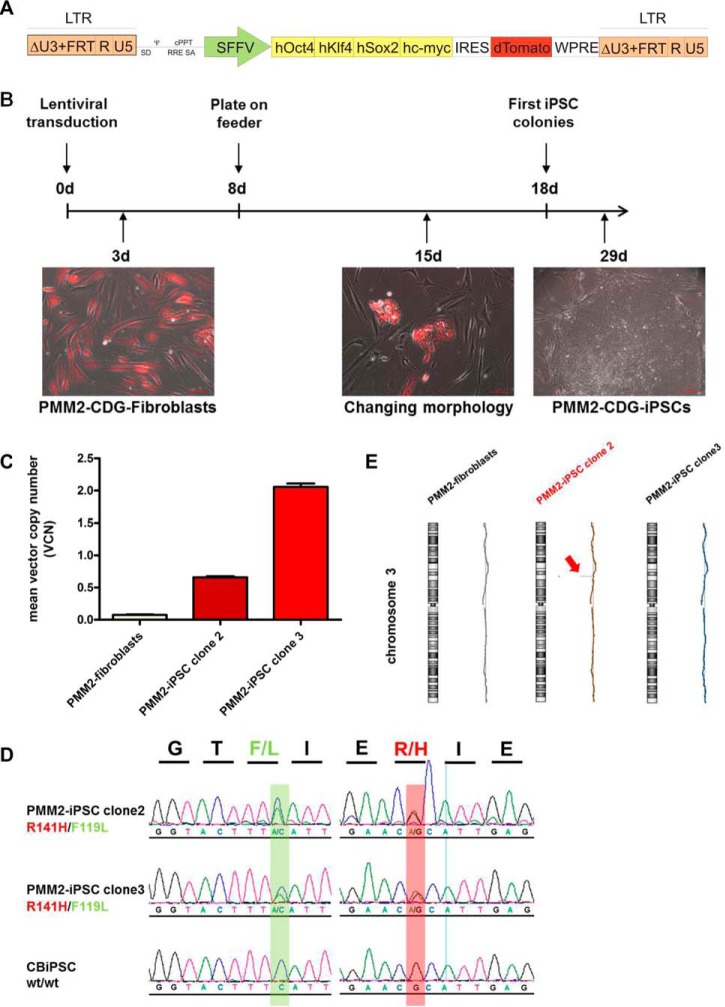
**Generation and genomic characterization of PMM2-iPSC-C3.** *A*, scheme of lentiviral reprogramming vector construct (modified from (20)). *B*, timeline of reprogramming. PMM2-fibroblasts 3 days (3d) post lentiviral transduction (left image), reprogrammed cells emerged on MEFs 15d post transduction (middle image), PMM2-iPSC colony on MEFs 29d post transduction (right image). *C*, determination of vector copy numbers. Number of integrated vector constructs were determined by qPCR (according to (24)). Bar diagram shows mean ± S.E. with *n* = 3 (PMM2-fibroblasts and PMM2-iPSC clone 2) and *n* = 2 (PMM2-iPSC clone 3). *D*, confirmation of the *PMM2* mutations in PMM2-iPSCs by sequencing. Sequence analysis revealed the two heterozygous point mutations 357C>A and 422G>A in the *PMM2* gene of the generated PMM2-iPSC clones 2 and 3. CBiPSC2 were used as control cells. *E*, Array-CGH analysis of PMM2-iPSC clone 2 and 3. The analysis of chromosomal integrity by Array-CGH revealed a deletion in the tumor suppressor gene *FHIT* on chromosome 3 in clone 2.

PMM2-iPSCs were reprogrammed from skin fibroblasts of a PMM2-CDG patient harboring the most frequent genomic *PMM2* mutational combination of 422G>A in one allele and 357C>A in the other allele. These fibroblasts were transduced with a polycistronic lentiviral vector construct expressing the reprogramming factors (RFs) OCT4, KLF4, SOX2, and c-MYC (20) (Fig. 1*A*). Upon transduction, expression of the red fluorescent protein dTomato could be monitored in the majority of cells indicating expression of RFs. In the course of 2 weeks, the fibroblasts changed their morphology from long and slim shape into compact roundish cells with high nucleus to cytoplasm ratio, which is typical for hiPSCs (Fig. 1*B*). PMM2-iPSC colonies with typical stem cell shape emerged after vector silencing, which is indicated by absence of dTomato expression 4 weeks post transduction (Fig. 1*B*, right picture). Of 40,000 fibroblasts transduced in two independent reprogramming approaches only three clones emerged and could be maintained in culture (PMM2-iPSC-C1, PMM2-iPSC-C2 and PMM2-iPSC-C3). One of these clones, PMM2-iPSC-C1, was excluded for further analyses because of morphological abnormalities. Because retroviral vector integrations can lead to gene deletion or proto-oncogene activation (31), we determined the lentiviral vector copy number integrations by qPCR. For PMM2-iPSC-C2 and -C3 one and two integrations were detected, respectively (Fig. 1*C*), suggesting minimal influences on global gene expression. Genetic analysis confirmed the patient specific hypomorphic *PMM2*^*422G*>*A/357C*>*A*^ mutations in both clones (Fig. 1*D*). As the reprogramming of somatic cells can cause chromosomal aberrations and unwanted mutations (32), we analyzed PMM2-iPSC-C2, PMM2-iPSC-C3 and the parental PMM2-fibroblasts by Array-CGH. This analysis revealed a deletion of a locus containing the tumor suppressor gene *FHIT* in PMM2-iPSC-C2 whereas no genomic aberrations were detected in PMM2-iPSC-C3 and parental fibroblasts (Fig. 1*E*). We therefore decided to perform the further studies with PMM2-iPSC-C3, only.

**Fig. 2.**
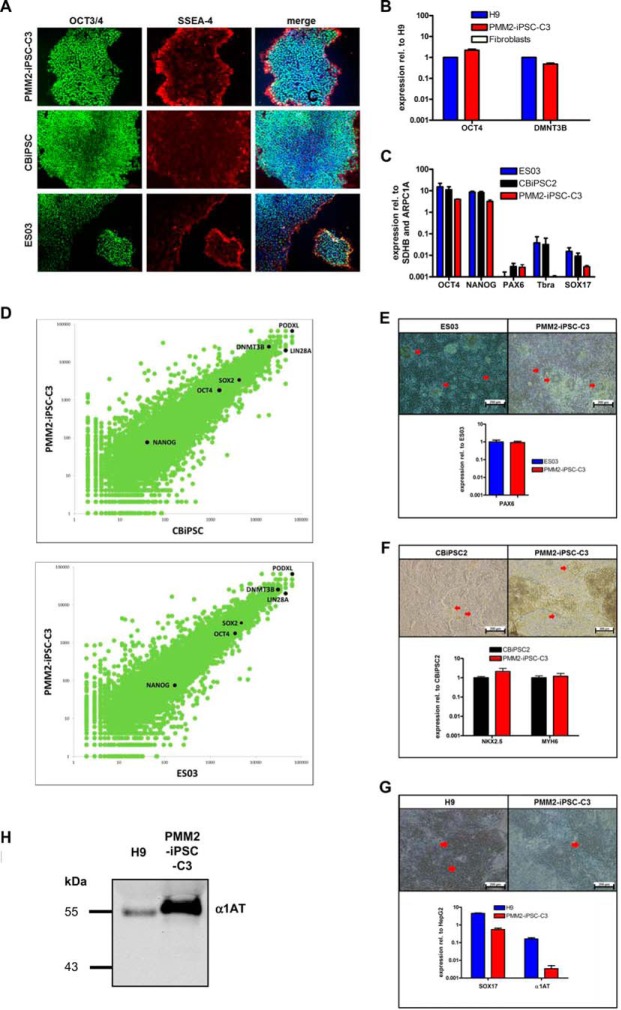
**PMM2-iPSC-C3 are pluripotent.** *A*, Immunocytochemistry of PMM2-iPSC-C3, CBiPSC2 and ES03 grown on Matrigel^TM^ with mTeSR^TM^1 using antibodies against the pluripotency markers OCT3/4 (green, left panel) and SSEA-4 (red, middle panel). Merge of OCT3/4, SSEA-4 and DAPI (blue, right panel). *B*, qPCR analysis of pluripotency markers *OCT4* (endogenous) *and DNMT3B* in H9, PMM2-iPSC-C3 and fibroblasts. Expression levels are normalized to levels in H9. *C*, qPCR analysis of the pluripotency markers *OCT4* and *NANOG* together with early differentiation markers *PAX6* (ectoderm), *T/Bra* (mesendoderm) and *SOX17* (endoderm) in ES03, CBiPSC2 and PMM2-iPSC-C3. Expression levels are relative to the housekeeping genes *succinate dehydrogenase complex subunit B* (*SDHB*) and *actin related protein 2/3 complex subunit 1A* (*ARPC1A*). *D*, whole genome expression analysis by deep-sequencing. Scatter plot of gene expression levels in PMM2-iPSC-C3 compared with CBiPSC2 (upper panel) and ES03 (lower panel). Expression of the pluripotency markers *NANOG*, *OCT4*, *SOX2*, *LIN28A*, *DNMTB3* and *PODXL* is depicted in black, 18456 transcripts were identified in total. *E*, neural differentiation. Formation of neural rosettes (upper panel) and qPCR analysis for expression of *PAX6* (lower panel) upon culture of ES03 and PMM2-iPSC-C3 for 10 days in STEMdiff^TM^ Neural Induction Medium (StemCell Technologies). *F*, cardiomyogenic differentiation. Light microscopic pictures (upper panel) and qPCR analysis of the cardiomyocyte-specific transcription factors *NKX2.5* and *MYH6* (lower panel) around day 10 of differentiation of CBiPSC2 and PMM2-iPSC2-C3. *G*, hepatic differentiation. Light microscopic pictures (upper panel) and qPCR analysis of the human embryonic stem cell line H9 as control and PMM2-iPSC-C3 at day 10 of differentiation for the endodermal markers *SOX17* and α*1AT* relative to the human liver carcinoma cell line HepG2. Bar diagrams show mean ± S.E., *n* = 3, except for PAX6 *n* = 2. *H*, Western blot against α1AT of proteins precipitated from culture supernatants of H9 and PMM2-iPSC-C3 upon hepatic differentiation for 13–15 days.

PMM2-iPSC-C3 displayed an ES cell-like morphology when cultivated either on mouse embryonic fibroblasts (MEFs, Fig. 1*B*, right picture) or on Matrigel^TM^ (Fig. 2*A*) and stained positive for OCT3/4 and SSEA-4 (Fig. 2*A*). Disappearance of dTomato expression from the reprogramming cassette suggested effective epigenetic silencing of the lentiviral construct (Fig. 1*B*). qPCR analysis revealed high expression levels of endogenous OCT4 and DMNT3B (33) in PMM2-iPSC-C3 (Fig. 2*B*) and only minimal expression of early differentiation markers like *PAX6* (ectoderm, (34)), *T/Brachyury* (mesoderm, (23)) or *SOX17* (endoderm, (35)). All these expression levels were comparable to controls ES03 and CBiPSC2 (Fig. 2*C*). Global gene expression of PMM2-iPSC-C3, assessed by deep-sequencing, revealed a closely related profile to the pluripotent control cell lines ES03 and CBiPSC2. Notably, expression levels of pluripotency markers *NANOG*, *OCT4*, *SOX2*, *LIN28*, *DNMT3B* and *PODXL* (33) were nearly identical. Furthermore, *PODXL*, *LIN28A* and *DNMT3B* belonged to the highest expressed genes in all three cell lines tested (Fig. 2*D*, supplemental Table S6). We assessed the capacity of PMM2-iPSC-C3 to be differentiated into the three germ layers applying directed *in vitro* differentiation. PMM2-iPSC-C3 could be differentiated into neural progenitor cells (ectoderm) showing typical rosette shaped morphology (36) and expression of the early ectodermal marker *PAX6* (Fig. 2*E*). Directed cardiomyogenic differentiation (mesoderm) was performed by temporal modulation of canonical Wnt-signaling (23) leading to the formation of beating foci with typical rhythmic contractions (supplemental Movie S1) unambiguously showing the generation of cardiomyocytes (37). These cells expressed the cardiomyocyte-specific marker genes *NKX2.5* and *MYH6* (38, 39) (Fig. 2*F*). A hepatic differentiation (endoderm) protocol (22) lead to the formation of cells showing a “cobble-stone”-like morphology that is typical for hepatic precursors and the expression of the early endoderm marker *SOX17* and the hepatocyte-specific marker alpha-1-antitrypsin (α1AT) (40) (Fig. 2*G*). α1AT-expression of hepatically differentiated PMM2-iPSC-C3 was also shown on the protein level by Western blot analysis (Fig. 2*H*).

##### Determination of Phosphomannomutase Enzymatic Activity and Analysis of Dolichol-linked Oligosaccharides

**Fig. 3.**
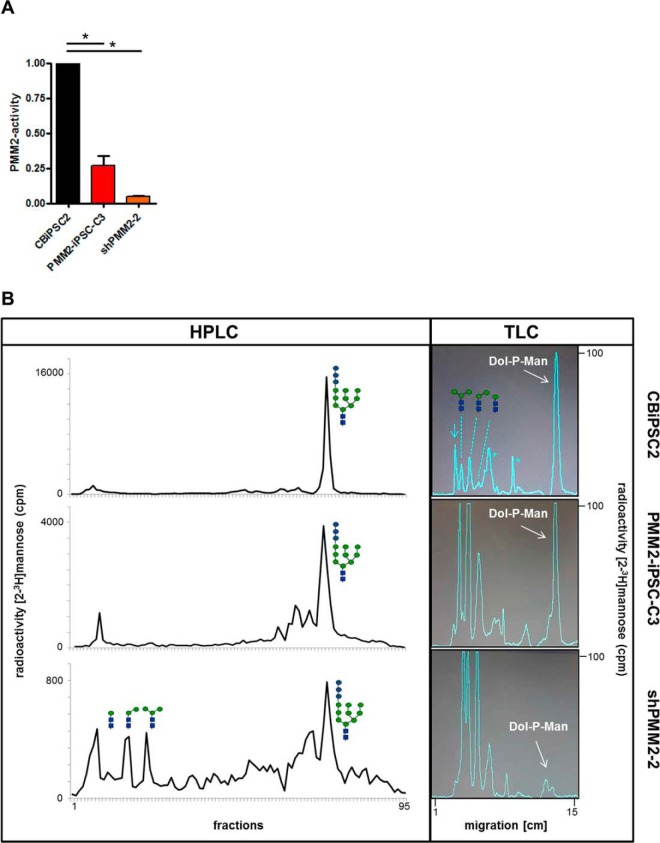
**Determination of phosphomannomutase activity and dolichol-linked oligosaccharides by HPLC and TLC.** *A*, phosphomannomutase activity was determined as the turnover of radio-labeled Man-6-P to Man-1,6-P in the presence of cofactor Glu-1,6-P from whole cell lysates that were adjusted to equal protein amounts. Bar diagrams show mean ± S.D. for CBiPSC2, PMM2-iPSC-C3 and knock-down PMM2-iPSC-C3 (shPMM2–2), CBiPSC2 activity was set to 1, for statistics a ratio t-Test was applied. *B*, analysis of dolichol-linked oligosaccharides was performed with 1.2 × 10^6^ of control (CBiPSC2), patient-derived (PMM2-iPSC-C3) and knock-down PMM2-iPSC-C3 (shPMM2–2) cells that were metabolically labeled with [2-^3^H]mannose. [^3^H]Oligosaccharides were released from dolichol and separated by HPLC (left) or thin layer chromatography (TLC; right). Used symbols: blue square: N-acetylglucosamine; green circle: mannose; blue circle: glucose; arrow: origin; asterisk: unspecific [^3^H]compound arising because of the starvation conditions before labeling.

The phosphomannomutase activity of PMM2-iPSC-C3 and PMM2-iPSC-C3 with an additional PMM2 knock-down (shPMM2–2, will be introduced below) was determined and compared with the healthy control hiPSC line CBiPSC2. As both, PMM1 and PMM2 catalyze the same reaction, discrimination between these phosphomannomutases is not possible and the measured activity is the sum of the activities of both enzymes. Protein-normalized cell lysates of PMM2-iPSC-C3 showed a reduction in phosphomannomutase activity that was 27 ± 5% compared with CBiPSC2, whereas the residual activity of shPMM2–2 was severely reduced to 5 + 0.5% (Fig. 3*A*). To determine whether the allocation of full-length oligosaccharide Glc3Man9GlcNAc2-PP-dolichol is affected because of the reduced phosphomannomutase activity, [2-^3^H]mannose-labeled oligosaccharides were extracted, released from dolichol and analyzed by HPLC. The main peak fraction of CBiPSC2 and PMM2-iPSC-C3 eluted with a Glc3Man9GlcNAc2 standard, respectively (Fig. 3*B*), whereas the structural composition of the oligosaccharides derived from the knock-down cell line (shPMM2–2) showed in addition shortened intermediates of the structures Man3GlcNAc2 to Man1GlcNAc2. In contrast to CBiPSC2 and PMM2-iPSC-C3, in which the full-length oligosaccharide predominates, whereas truncated structures with three or fewer mannose residues represent between 5.7–6.9% of all [^3^H]oligosaccharides, this level was elevated to 17.4% in the knock-down cell line (shPMM2–2). When we compared the overall radioactivity incorporated into the shortened dolichol-linked sugars with the amount of [^3^H] found in the Glc3Man9GlcNAc2 fractions, it even became more obvious that in case of CBiPSC2 and PMM2-iPSC-C3, 13.2% and 18.4% of the radioactivity was found in the early HPLC fractions, respectively, whereas in the knock-down cells the level was increased to 72.8%.

Next we determined the incorporation of [2-^3^H]mannose into Dol-P-Man which is found together with the shortened dolichol-linked oligosaccharides in the chloroform/methanol extracts. Although in the CBiPSC2 cells 42.6% of the overall radioactive mannose measured was found as Dol-P-Man, only 23.9% and 6.7% were detected in case of PMM2-iPSC-C3 and the knock-down cells, respectively. Finally, we were also interested to see if the PMM2 deficiency led to a reduced incorporation of [2-^3^H]mannose structures into nascent glycoproteins by comparing the incorporation of [2-^3^H]mannose to that of [^35^S]methionine into TCA insoluble material. The [^3^H]/[^35^S] ratio in PMM2-iPSC-C3 was 71.08% of the control and was further decreased to 24.3% in the knock-down cells.

##### Lectin Analysis Revealed Reduced Levels of High-mannose-type N-glycans on Cell Surface Proteins of PMM2-iPSC-C3

**Fig. 4.**
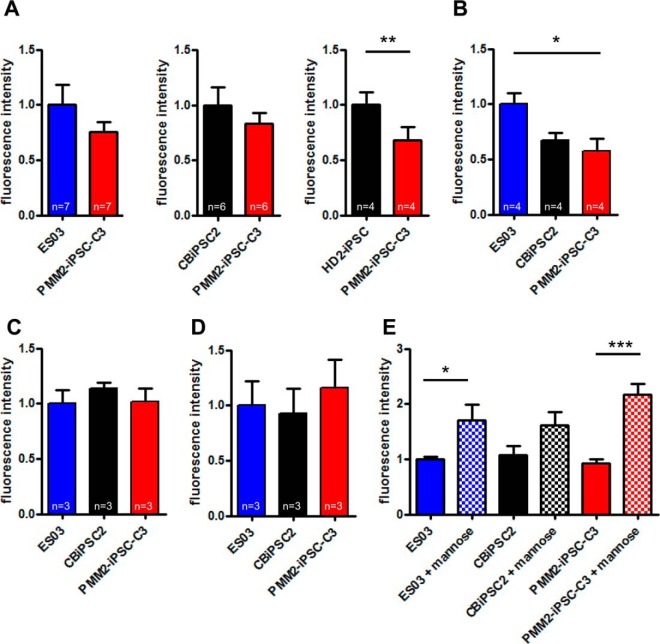
**Quantification of mannosylation by lectin flow cytometry.** *A* to *D*, flow cytometry with the indicated cell lines was performed with GNA-FITC (*A*), Con A-TRITC (*B*), SNA-TRITC (*C*) and MAA-FITC (*D*) as glycan specific reagents. Bar diagrams display means of fluorescence intensities + S.E. for the respective cell lines. Numbers of independent replicates are indicated in the figure, a paired Student's *t* test was performed and statistical significant differences (*p* < 0.05) are highlighted. *E*, quantification of cell surface mannosylation after culture in medium supplemented with 10 mm mannose by GNA-FITC flow cytometry; bar diagrams show mean ± S.E., unpaired Student's *t*-test was performed between each of the cell lines with and without additional nutritional mannose, *n* = 4 for samples without mannose, *n* = 3 for samples with mannose.

Patients with hypomorphic Arg^141^His and Phe^119^Leu alleles of PMM2 display reduced N-glycosylation of serum glycoproteins (41). Here we used the patient-derived PMM2-iPSC-C3 in order to study the effect of these mutations on N-glycosylation in the pluripotent state. To quantitatively assess cell surface glycosylation, flow cytometry with different lectins was performed. Binding of FITC-labeled *Galanthus nivalis* agglutinin (GNA)—preferentially detecting α1,3- and α1,6-linked terminal mannose of high-mannose-type N-glycans (42, 43)—to viable cells of PMM2-iPSC-C3 was reduced in comparison to different control hPSCs (ES03, CBiPSC2, HD2-iPSC) (Fig. 4*A*). The specificity for binding of GNA-FITC to mannosylated structures on cells was confirmed by competition with mannose during staining, which considerably reduced GNA-FITC binding to cells (supplemental Fig. S1*A*). Concordantly, binding of the lectin GNA to blotted proteins of PMM2-iPSC-C3 was slightly impaired in comparison to control hPSCs (ES03, CBiPSC2) under physiological as well as under low glucose culture conditions (supplemental Fig. S1*B*). Flow cytometry using TRITC-labeled concanavalin A (Con A), detecting high-mannose-type N-glycans with highest affinity (44), also revealed reduced binding to PMM2-iPSC-C3 if compared with ES03 (Fig. 4*B*). These findings indicate diminished mannosylation of N-glycans expressed at the cell surface in PMM2-iPSC-C3. Flow cytometry using the lectins *Sambucus nigra* agglutinin (SNA, Fig. 4*C*) or *Maackia amurensis* agglutinin (MAA, Fig. 4*D*), which are specific for sialic acid attached to galactose in either α-2,6 or α-2,3 linkage, respectively, did not reveal any differences between PMM2-iPSC-C3 and control cell lines. Culturing of PMM2-iPSC-C3 and control hPSCs (ES03 and CBiPSC2) for at least three passages in mTeSR^TM^1 medium supplemented with 10 mm mannose increased binding of GNA-FITC in all cell lines tested (Fig. 4*E*, supplemental Fig. S1*B*). This mannose supplementation did not obviously change stem cell morphology or growth behavior.

##### PMM2-iPSC-C3 Display Characteristic but Reduced N-glycan Levels

**Fig. 5.**
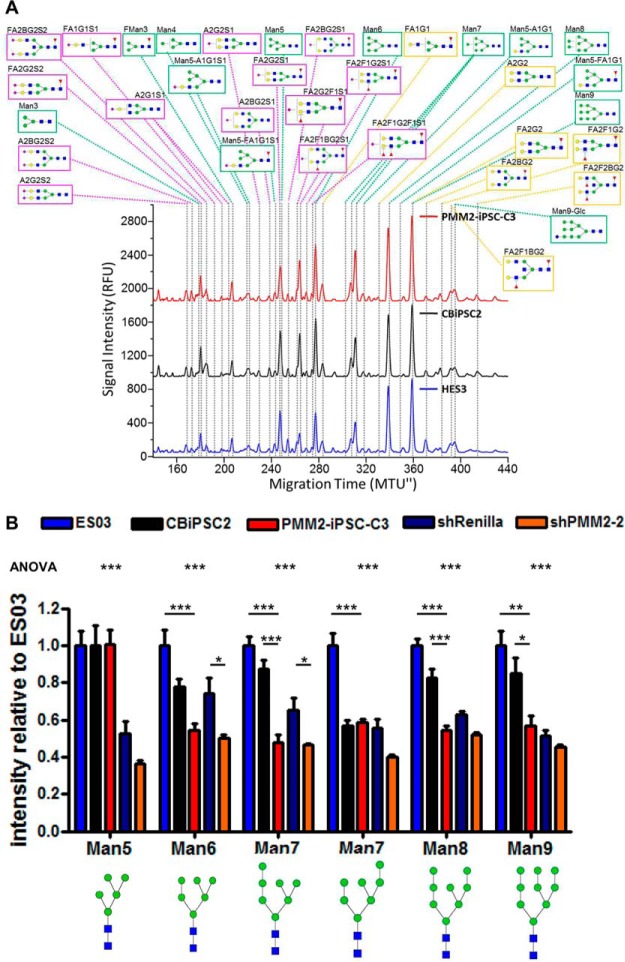
**Analysis and quantification of PMM2-iPSC-C3 N-glycans.** *A*, electropherograms of PMM2-iPSC-C3, CBiPSC2 and ES03 derived N-glycans, labeled with APTS and analyzed by xCGE-LIF. Peaks were annotated depending on migration time matching with an in-house N-glycan database and exoglycosidase digests (supplemental Fig. S2). This electropherogram shows representative data of three independent experiments. An intra-sample relative quantification of the different N-glycan levels is presented as supplemental Table S7. *B*, inter-sample relative quantitative comparison of levels of the most abundant high-mannose-type N-glycans of ES03, CBiPSC2, PMM2-iPSC-C3, PMM2-iPSC-C3 transduced with shRenilla (will be introduced below) and PMM2-iPSC-C3 transduced with shPMM2–2 (will be introduced below). Individual spectra were quantitatively normalized to a defined spiked-in asialofetuin N-glycan. Bar diagram shows mean ± S.E., *n* = 3 each. The mean intensity of ES03 was set to 1 and intensities for other cell lines are relative to ES03. For statistics ANOVA with Bonferroni's post hoc test was used. *A* and *B*, N-glycan structures were drawn with GlycoWorkbench v.1.0.3353 (84) by the guidelines of the Consortium for Functional Glycomics (85). Green circle: mannose; yellow circle: galactose; blue quadratic rectangle: N-acetylglucosamine; violet diamonds: sialic acid; red triangle: fucose. Quantification of less abundant N-glycans including hybrid- and complex-type N-glycans is presented as supplemental Fig. S3.

N-glycosylation of PMM2-iPSC-C3 was analyzed by multiplexed capillary gel electrophoresis with laser induced fluorescence detection (xCGE-LIF (30, 45–48)) and compared with the hPSC lines ES03 and CBiPSC2. Identification and annotation of N-glycans was carried out based on our internal N-glycan database and by comprehensive exoglycosidase digests (supplemental Fig. S2). We could clearly assign 61 different N-glycan species subdividing into 13 high-mannose-type and 48 complex-type N-glycans in all investigated cell lines. This approach enabled an intra-sample relative quantitative assessment of N-glycan intensities and clearly demonstrated that the N-glycosylation patterns of PMM2-iPCS-C3, ES03 and CBiPSC2 were similar and high-mannose-type N-glycans were the predominant species (Fig. 5*A*, supplemental Table S7). Interestingly, treatment with the α(2,3)-sialic acid-specific sialidase S from *Streptococcus pneumoniae* did not affect the xCGE-LIF peak pattern in any of the human pluripotent stem cell lines analyzed (ES03, CBiPSC2, PMM2-iPSC-C3), proving that hPSCs at least under the tested culture conditions do not produce α(2,3)-sialylated N-glycans (supplemental Fig. S2).

xCGE-LIF, as described above, is capable to decipher even minor changes in the N-glycosylation pattern between two samples, but is not a quantitative method allowing the detection of a general reduction of all N-glycans. As the latter case would be expected for cells with a mutation in the PMM2, we further developed the xCGE-LIF-based method in order to allow quantitative comparison between different samples (inter-sample quantification). For that purpose, asialofetuin was spiked into each sample in equal amounts as an internal standard. The asialofetuin derived N-glycans were proven to migrate at positions where they do not overlap with peaks of the stem cell glycans. All N-glycan fingerprints were then normalized to the height of one asialofetuin N-glycan peak, enabling a quantitative comparison of sample peaks. Using this method, we quantified 22 N-glycans. Peak intensities of the 6 most abundant high-mannose-type N-glycans (Man5 to Man9) were reduced by up to 40% in PMM2-iPSC-C3 compared with ES03 and CBiPSC2 (Fig. 5*B*). The xCGE-LIF-based quantitative comparison of hybrid- and complex-type N-glycans between ES03, CBiPSC2 and PMM2-iPSC-C3 did not show a common pattern (supplemental Fig. S3). However, it is likely that the exact quantification of these glycan species is hindered by their low appearance.

##### RNAi Knock-down of PMM2 in PMM2-iPSC-C3 Further Reduces Mannosylation

**Fig. 6.**
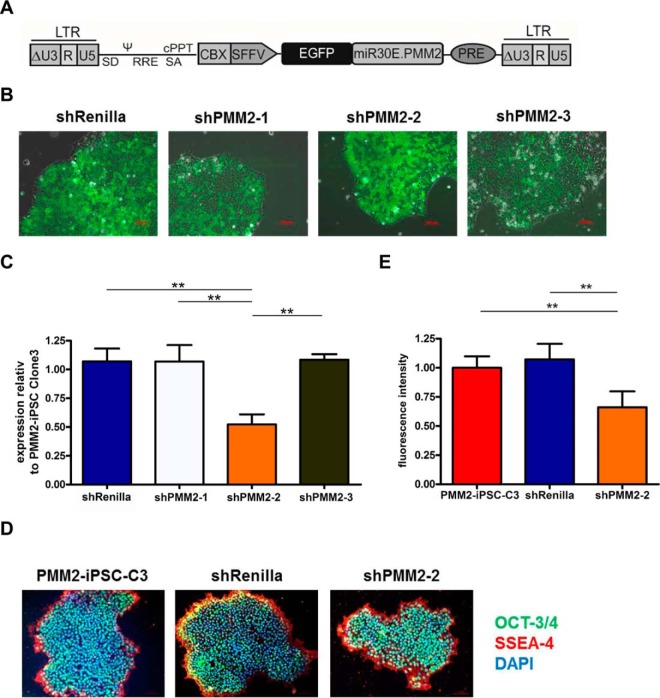
**Knock down of *PMM2* in PMM2-iPSC-C3 by RNAi.** *A*, Scheme of the lentiviral vector construct used for expression of shRNAs. *B*, transduction of shRNA constructs (shRenilla (negative control), shPMM2–1 to 3 (constructs targeting *PMM2*)) was monitored by eGFP fluorescence. *C*, qPCR-based quantification of *PMM2*-expression in *PMM2*-knock down cell lines relative to PMM2-iPSC-C3. Bar diagrams show mean ± S.E., *n* = 3. Statistical analysis was done with ANOVA and Bonferroni's multiple comparison post hoc test. *D*, immunocytochemistry for expression of pluripotency markers OCT3/4 (green) and SSEA-4 (red). Nuclei are counterstained with DAPI (blue). *E*, quantification of mannosylated cell surface glycoconjugates by lectin-flow cytometry with GNA-Texas Red for PMM2-iPSC-C3, PMM2-iPSC-C3 transduced with shRenilla and PMM2-iPSC-C3 transduced with shPMM2–2. Bar diagrams show mean ± S.E., *n* = 3. Statistical analysis was done with ANOVA and Bonferroni's multiple comparison post hoc test.

In order to study the consequences of a further reduction of PMM2 activity on glycosylation at the stem cell level, we performed a knock-down of *PMM2* in PMM2-iPSC-C3 by RNAi. We applied a lentiviral vector expressing shRNAs embedded in an optimized mir-30 backbone (21) driven by the SFFV promoter, which is coupled to the minimal ubiquitous chromatin opening element CBX3 (49) to prevent promoter silencing for long-term stable shRNA expression in iPSCs (Fig. 6*A*). PMM2-iPSC-C3 were transduced with different *PMM2*-specific shRNAs (shPMM2–1, shPMM2–2, shPMM2–3) and a negative control shRNA that was designed against the luciferase from *Renilla reniformis* (shRenilla) as a default target. All constructs harbor a GFP sequence 5′ of the shRNA, enabling monitoring shRNA expression (Fig. 6*B*). shPMM2–2 induced a significant *PMM2* knock-down of about 40–50% in GFP^+^-sorted cells as assessed by qPCR. shPMM2–1 and shPMM2–3 did not cause any knock-down of *PMM2* and were therefore excluded from the following analysis. As expected, shRenilla had no influence on *PMM2* expression levels (Fig. 6*C*). PMM2-iPSC-C3 transduced with shPMM2–2 and control vector (shRenilla) still expressed OCT3/4 and SSEA-4, suggesting that pluripotency was unaffected during further cultivation (Fig. 6*D*). Cell surface mannosylation was assessed by GNA-lectin flow cytometry as described above. PMM2-iPSC-C3 transduced with shPMM2–2 displayed a significant reduction of cell surface mannosylation of about 30% compared with shRenilla-transduced control cells and the parental PMM2-iPSC-C3 (Fig. 6*E*). These observations are in concordance with the quantitative xCGE-LIF analyses (Fig. 5*B*).

## DISCUSSION

PMM2-CDG, the most common type of the rare disease family of congenital disorders of glycosylation is a severe inherited congenital disorder affecting N-glycosylation, which is often fatal and generally associated with major developmental defects. The pathophysiology of PMM2-CDG is poorly understood (50–52) and with more than 50% of all human proteins being N-glycosylated (53), PMM2-CDG is expected to affect a tremendous complexity of protein functions.

Several models to study PMM2-CDG have been applied so far, including human fibroblasts (54, 55), mice (13), zebrafish (11) and *Xenopus laevis* (10). By using fibroblasts from PMM2-CDG patients the biochemical phenotypes of this disease on metabolites in the pathway of PMM2 were unraveled (9) but fibroblasts cannot model the complex relationships present in specific tissues, organs or even whole organisms. Animal models are ideal tools to study PMM2-CDG at the organismal level but their complexity impedes a molecular correlation between distinct glycosylation defects and pathological phenotypes.

An alternative toward a better understanding of PMM2-CDG would be a human cell culture model enabling to study the effects of PMM2-CDG during embryonic development. Since the invention of cellular reprogramming (15, 56) various genetic diseases including neurodegenerative and cardiac disorders, hematopoietic maladies and various metabolic diseases have been modeled with iPSCs (16, 17). The iPSC-technology is especially attractive for the study of rare diseases. For these studies iPSCs display a high potential for infinite expansion and molecular characterization whereas primary material is often limited. Therefore, we generated a human PMM2-CDG iPSC model by reprogramming of PMM2-CDG patient-derived fibroblasts and characterized glycosylation of these PMM2-iPSCs in depth.

The PMM2-CDG iPSC cell culture model presented here fills the gap between fibroblast and animal models. PMM2-iPSC-C3 display the features of completely reprogrammed “bona fide” iPSCs and therefore present a theoretically indefinite source of stem cells and differentiated progeny. Our model enables to study the disease pathology and underlying molecular mechanisms already on the stem cell level. Furthermore, PMM2-iPSC-C3 could be differentiated into cell types from all three embryonic germ layers providing the opportunity to study this specific glycosylation defect in a variety of more specialized cell types of the human body.

Interestingly, the observed reprogramming efficiency of 0.008% was rather low for PMM2-fibroblasts compared with studies with different genes being affected, where the authors reported efficiencies of about 0.1–1% with a similar 4in1 lentiviral vector construct (57). Reprogramming efficiencies are affected by diverse parameters including the age of the donor (58) or the quality and viability of primary fibroblasts in culture before and during reprogramming. But also loss-of-function mutations can restrict reprogramming efficiency as it has been shown for reprogramming of Fanconi anemia cells (59). In our study it cannot be excluded that the decreased reprogramming efficiency of PMM2-CDG fibroblasts is associated with the functional mutation of PMM2 affecting N-glycosylation. By two independent reprogramming approaches only three PMM2-iPSC clones were obtained, of which two had to be discarded because of phenotypic or genomic aberrations. In the latter case a deletion in the tumor suppressor gene *FHIT* was observed by Array-CGH, which could reflect a typical phenomenon during reprogramming caused by genomic instability (60) or could be the consequence of high selection pressure because of restricted reprogramming caused by PMM2 dysfunction. Functional correction by retroviral transfer of the *PMM2* gene could give an indication if the loss of enzymatic function directly decreases reprogramming efficiency. However, based on morphology, gene expression analysis and *in vitro* differentiation capacity, the selected clone PMM2-iPSC-C3 displayed all critical features of completely reprogrammed functional iPSCs (61).

Two highly conserved phosphomannomutases (PMM1 and PMM2) exist in humans and both can convert Man-6-P to Man-1-P *in vitro* (62). However, deletion of *Pmm1* in mice is neither associated with any obvious phenotype nor with hypoglycosylation (63), whereas disruption of the *Pmm2* gene causes early embryonic lethality (12). The latter observation and the severe disease phenotype of PMM2-CDG patients, in which PMM1 is unaffected apparently indicates that PMM1 cannot compensate for PMM2 activity. Residual phosphomannomutase activity in PMM2-iPSC-C3 was determined to be about 27% of nondiseased hPSCs. Deep-sequencing revealed that expression of *PMM2* was 5–10-fold higher than *PMM1* expression in hPSCs and we therefore argue that the observed phosphomannomutase *in vitro* activity in hPSCs is mainly because of PMM2. These findings led to the hypothesis that PMM2 might be the phosphomannomutase, which is essential during early embryonic development when high-mannose-type N-glycan structures are predominant (64). PMM1 was shown to be highly expressed perinatally in mice (63) and we hypothesize that later in development PMM1 together with defective PMM2 are capable to restore the hypoglycosylation phenotype. In adult PMM2-CDG patient's serum glycoproteins have been shown to be normally glycosylated (65, 66). However, malformations that were acquired during early phases of development cannot be reversed.

The observed residual PMM2 activity in PMM2-iPSC-C3 differs from the reported parental PMM2-CDG fibroblasts activity of about 10% (NIGMS Human Genetic Cell Repository data sheet), which is a typical activity value observed in severely affected PMM2-CDG patients (67). The method used in the present study calculates phosphomannomutase activity in a direct way as it measures the turnover of radio-labeled Man-6-P to Man-1,6-bisphosphate. In contrast, the method described by van Schaftlingen and Jaeken (8) determines phosphomannomutase activity in a coupled enzymatic assay where the activity is determined indirectly, as the conversion of NADP to NADPH by glucose-6-phosphate dehydrogenase. The differences between both methods could account for the observed discrepancy between phosphomannomutase activity of PMM2-fibroblasts and PMM2-iPSC-C3. Besides, the relatively high residual enzymatic activity presumably also prevented accumulation of shorted dolichol-linked oligosaccharides, which are a characteristic biochemical hallmark in PMM2-CDG, and which was observed for PMM2-iPSC-C3 transduced with an shRNA against PMM2 (shPMM2–2). Nevertheless, the total amount of the full-length oligosaccharide Glc3Man9GlcNAc2, the incorporation of radioactively labeled mannose into proteins and Dol-P-Man synthesis were clearly diminished in case of the PMM2-iPSC-C3 and further reduced in PMM2-iPSC-C3 transduced with shPMM2–2, clearly demonstrating the impact of the decreased PMM2 activity on the allocation of the LLOs.

Lectin blotting with GNA, which strongly binds to high-mannose-type N-glycans (42, 43) indicated hypoglycosylation of proteins in PMM2-iPSC-C3. We additionally performed flow cytometry with the fluorescently labeled lectins GNA and Con A. Con A also preferentially binds to high-mannose-type N-glycans (44) and is not known to bind to O-glycans (68). This analysis was performed on intact cells and showed reduced levels of cell surface exposed high-mannose-type N-glycans, which could be a consequence of membrane protein hypoglycosylation or reduced incorporation of glycoproteins into the plasma membrane, as recently shown for hypoglycosylated ICAM-1 (69). By spiking-in a defined reference glycoprotein (asialofetuin) to our samples, we here present the application of xCGE-LIF for quantitative comparison between different samples. Using protein normalized samples, this approach revealed a clear reduction of high-mannose-type N-glycans in the overall proteome of PMM2-iPSC-C3 in comparison to nondiseased hPSCs. Hybrid- and complex-type N-glycans were only present in low abundance on hPSCs impeding their quantification. These observations are in concordance with common knowledge that PMM2-CDG causes hypoglycosylation of serum proteins in PMM2-CDG patients (41) and in a previously published PMM2-iPSC model reduced glycosylation of PMM2-iPSCs was shown indirectly using an ER-resident N-glycosylation responsive GFP (70). In line with previous studies reporting reduced N-glycosylation site occupancy but unaffected distribution of different N-glycan species (71), our xCGE-LIF analyses (30, 45–48) showed that the N-glycosylation pattern is not affected in PMM2-iPSC-C3. We found high-mannose-type N-glycans (Man5 to Man9) to be the predominant glycan species accounting for about 70% of all N-glycans, which is concordant to previous findings for hESCs and hiPSCs (64, 72). Our xCGE-LIF analysis combined with exoglycosidase digests clearly revealed that sialic acids on N-glycans of hPSCs are exclusively α(2,6)-linked. This observation has been reported before (72, 73) and might affect the interaction of cell surface glycans with endogenous lectins, which is known to affect pluripotency of hPSCs (74). Overexpression of α(2,6)-linked sialic acid is observed in many types of human cancers (75) and might protect tumor cells from galectin-mediated apoptosis (76). The fact that sialylated complex-type N-glycans are present only in minor amounts on hPSCs together with the absence of α(2,3)-linked sialic explains the failure to identify differences by flow cytometry with the lectins MAA and SNA.

In PMM2-CDG patient derived fibroblasts reduced GDP-mannose levels have been rescued by adding mannose to the culture medium (54, 55, 77). In the present study it could be proven that mannose supplementation was sufficient to reverse hypoglycosylation in the embryonic state as seen in the PMM2-iPSC-C3. This observation is in line with recent findings that dietary mannose supplementation in mice during pregnancy is of therapeutic importance as it prevented manifestation of the PMM2-CDG disease phenotype in the offspring (13). Interestingly, supplementation of culture media with mannose increased the level of mannosylated cell surface glycoconjugates in all tested hPSCs suggesting that the glycome is a rather flexible system that responds to environmental conditions and could reflect the nutritional state of cells (78).

Diagnostics are based on serum transferrin isoelectric focusing, showing an isoform shift from predominant tetrasialotransferrin in healthy individuals to di- and asialotransferrin in PMM2-CDG patients (5). Hypoglycosylated variants of the liver derived serum protein alpha-1-antitrypsin (α1AT) serve as diagnostic marker for CDGs in the first three months of life when the standard marker transferrin is partially not yet fully matured (79). In our study, the molecular weight of secreted α1AT derived from hepatically differentiated H9 or PMM2-iPSC-C3 was slightly above 55 kDa indicating full glycosylation (80) and no variants of α1AT with lower molecular weight were detected as previously shown by de la Morena-Bario *et al.* in the plasma of PMM2-CDG patients (81). However, Dupre *et al.*, observed hypoglycosylation of α1AT in sera of PMM2-CDG patients but not in fibroblasts after a few passages in cell culture (82). The authors suggest that α1AT hypoglycosylaton is tissue specific. Obviously in a cell culture model the nutritional limitations that are present in the liver cannot be recapitulated. However, under physiological conditions the great majority of mannose in N-glycans is derived from glucose (83). Culture conditions, which are needed for maintenance of human pluripotent stem cells contain glucose above physiological levels, leading to increased levels of mannose available for N-glycosylation. Under these conditions the residual PMM2 activity of 27% is likely to be sufficient to prevent major limitations in N-glycosylation. Therefore, the observed unaltered glycosylation of α1AT in PMM2-iPSC-C3-derived hepatic cells could be caused by the high glucose culture conditions that are, however, needed to sustain pluripotent stem cells and for the differentiation into hepatic cells. The nutritional conditions in cell culture would also explain why the observed hypoglycosylation in PMM2-iPSC-C3 was only moderate and did not affect gene expression or pluripotency including differentiation capacity.

Thus we additionally performed an RNAi mediated *PMM2* knock-down in PMM2-iPSC-C3 in order to improve the model character of the generated PMM2-iPSC-C3 under the rich nutritional stem cell culture conditions. Additionally, to prove the postulated correlation of reduced PMM2 activity and hypoglycosylation we intended to compare PMM2-iPSC-C3 with their knock-down counterparts having an isogenic background. The shRNAs used in this study are designed in a way that they are processed by the cell specific miRNA biogenesis pathways and therefore are also known as “shRNAmirs.” “ShRNAmirs” are able to mediate potent knock-down and have the advantage that they are expressed by an RNA polymerase II promoter and can be cloned in the 3′UTR of a reporter, like GFP, to monitor shRNA expression. Furthermore, these artificial microRNAs show a lower cellular toxicity as conventional shRNAs when interfering with the miRNA machinery (21).

*PMM2* knock-down was successfully performed in PMM2-iPSC-C3 and led to biochemical characteristics known from PMM2-CDG fibroblasts, as severely reduced PMM2 activity, accumulations of shortened dolichol-linked oligosaccharides and a decreased level of Dol-P-Man. Additionally a further reduction of cell surface mannosylation in comparison to PMM2-iPSC-C3 was detected, which could be observed even at cell passage 40 (data not shown), indicating the stability of the shRNA expression during prolonged cell culture. Therefore, the generated iPSC knock-down cell line can serve as a further PMM2-CDG specific model to study molecular mechanisms of PMM2-CDG on the stem cell level and their differentiated progeny.

**Fig. 7.**
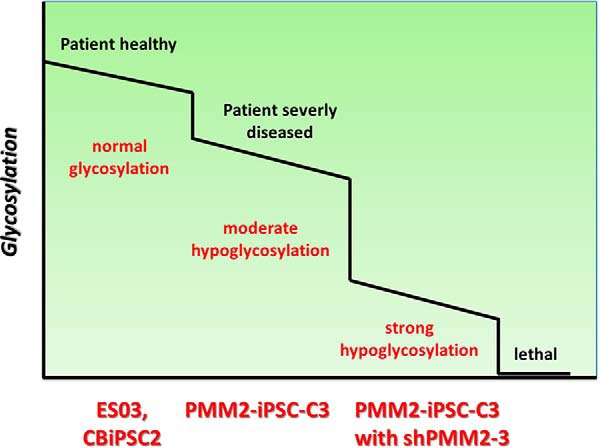
**Scheme for N-glycosylation in PMM2-IPSC models.** N-glycosylation is gradually reduced in PMM2-iPSC and PMM2-iPSC with *PMM2* knock-down compared with control iPSCs. These model cell lines enable studying the effect of different levels of N-glycosylation on embryonic development in a human cell culture system.

Taken together in this study we describe the generation of PMM2-iPSC and PMM2-iPSC knock-down models (Fig. 7). Reduced PMM2 activity causes hypoglycosylation already in human pluripotent stem cells that can be rescued by mannose feeding. These models have proved suitable to further study the effect of reduced glycosylation during early embryonic development *in vitro* in order to improve our understanding of PMM2-CDG disease pathophysiology.

## Supplementary Material

Supplemental Data

## Footnotes

^1^ The abbreviations used are:

CDG: congenital disorder of glycosylation
Array-CGH: array comparative genomic hybridization
bFGF: basic fibroblast growth factor
CBiPSC2: human cord blood derived induced pluripotent stem cell clone 2
EGF: epidermal growth factor
ES03: human embryonic stem cell line ES03 (HES-3)
GFP: green fluorescent protein
Glu-1,6-P: glucose-1,6-bisphosphate
H9: human embryonic stem cell line WA09
HCM: hepatocyte culture medium
HD2-iPSC: healthy donor derived induced pluripotent stem cell clone 2
hESC: human embryonic stem cell
(h)iPSC: (human) induced pluripotent stem cell
(h)PSC: (human) pluripotent stem cell
huEFs: human embryonic fibroblasts
IF: immunofluorescence
LLO: lipid-linked oligosaccharide
Man-1-P: mannose-1-phosphate
Man-1,6-P: mannose-1,6-bisphosphate
Man-6-P: mannose-6-phosphate
MEF: murine embryonic fibroblast
MEF-CM: murine embryonic fibroblast-conditioned medium
NPC: neural progenitor cell
PMM2: phosphomannomutase 2
PMM2-iPSCs: induced pluripotent stem cells reprogrammed from fibroblasts of a PMM2-CDG patient
RF: reprogramming factor
RI: Rho-associated coiled coil kinase inhibitor Y27632
xCGE-LIF: multiplexed capillary gel electrophoresis coupled to laser induced fluorescence detection.
