# Supplementary material for: Glycomic Characterization of Induced Pluripotent Stem Cells Derived from a Patient Suffering from Phosphomannomutase 2 Congenital Disorder of Glycosylation (PMM2-CDG)
Source: Mol Cell Proteomics. 2016 Jan 19;15(4):1435–52. doi: 10.1074/mcp.M115.054122 (PMC4824866; doi:10.1074/mcp.M115.054122)
Supplement: Supplemental Data [file supp_15_4_1435__index.html]

Glycomic characterization of induced pluripotent stem cells derived from a patient suffering from phosphomannomutase 2 congenital disorder of glycosylation — Glycomic characterization of induced pluripotent stem cells derived from a patient suffering from phosphomannomutase 2 congenital disorder of glycosylation — Glycomic Characterization of Induced Pluripotent Stem Cells Derived from a Patient Suffering from Phosphomannomutase 2 Congenital Disorder of Glycosylation (PMM2-CDG) — Disease Modeling of PMM2-CDG with iPSCs — Supplemental Data 

# Glycomic Characterization of Induced Pluripotent Stem Cells Derived from a Patient Suffering from Phosphomannomutase 2 Congenital Disorder of Glycosylation (PMM2-CDG)

## Supplemental Data

- Supplemental Material (.pdf, 8.8 MB) - Supplemental Figures S1 to S3 Supplemental Tables S1 to S7
- Supplemental Table S6 (.xlsx, 4.4 MB) - SUPPL. TABLE S6 related to Fig. 2D: Deep-sequencing-based transcriptomics for comparison of gene expression of ES03, CBiPSC2 and PMM2-iPSC-C3.
- Supplemental Movie S1 (.wmv, 22.9 MB) - SUPPL. MOVIE S1 related to Fig. 2: Differentiation of PMM2-iPSC-C3 into cardiomycytes
