# Supplementary material for: Glycomic Characterization of Induced Pluripotent Stem Cells Derived from a Patient Suffering from Phosphomannomutase 2 Congenital Disorder of Glycosylation (PMM2-CDG)
Source: Mol Cell Proteomics. 2016 Jan 19;15(4):1435–52. doi: 10.1074/mcp.M115.054122 (PMC4824866; doi:10.1074/mcp.M115.054122)
Supplement: Supplemental Data [file 10.1074_M115.054122_mcp.M115.054122-1.pdf]

SUPPLEMENTAL MATERIAL

Glycomic characterization of induced pluripotent stem cells derived from a patient suffering from phosphomannomutase 2 congenital disorder of glycosylation

Christina T. Thiesler[1,2,§], Samanta Cajic[3,§], Dirk Hoffmann[1,4], Christian Thiel[5], Laura van Diepen[6], René Hennig[3,7], Malte Sgodda[1,8], Robert Weißmann[6], Udo Reichl[3], Doris Steinemann[1,9], Ulf Diekmann[10], Nicolas M. B. Huber[1,2], Astrid Oberbeck[1,2], Tobias Cantz[1,8], Andreas W. Kuss[6], Christian Körner[5], Axel Schambach[1,4], Erdmann Rapp[3,7] and Falk F. R. Buettner[1,2,*]

[1]REBIRTH-Cluster of Excellence, Hannover Medical School, 30625 Hannover, Germany

[2]Institute for Cellular Chemistry, Hannover Medical School, 30625 Hannover, Germany

[3]Max Planck Institute for Dynamics of Complex Technical Systems, 39106 Magdeburg, Germany

[4]Institute of Experimental Hematology, Hannover Medical School, 30625 Hannover, Germany

[5]Center for Child and Adolescent Medicine, Department Kinderheilkunde I, 69120 Heidelberg, Germany

[6]Department for Human Genetics, University Medicine Greifswald and Interfaculty Institute for Genetics and Functional Genomics, Ernst-Moritz-Arndt University, 17475 Greifswald, Germany

[7]glyXera GmbH, 39120 Magdeburg, Germany

[8]Translational Hepatology and Stem Cell Biology, Dept. of Gastroenterology, Hepatology, and Endocrinology, Hannover Medical School, 30625 Hannover, Germany

[9]Institute of Cell and Molecular Pathology, Hannover Medical School, 30625 Hannover, Germany

[10]Institute of Clinical Biochemistry, Hannover Medical School, Hannover, Germany

*To whom correspondence should be addressed: Institute for Cellular Chemistry, Hannover Medical School, Carl-Neuberg-Strasse 1, 30625 Hannover, Germany. Tel.: +49(0)0511/532-8245; Fax: +49(0)511/532-8801; E-mail: buettner.falk@mh-hannover.de

[§]These authors contributed equally to this study

Supplemental Figures

Figure S1

A

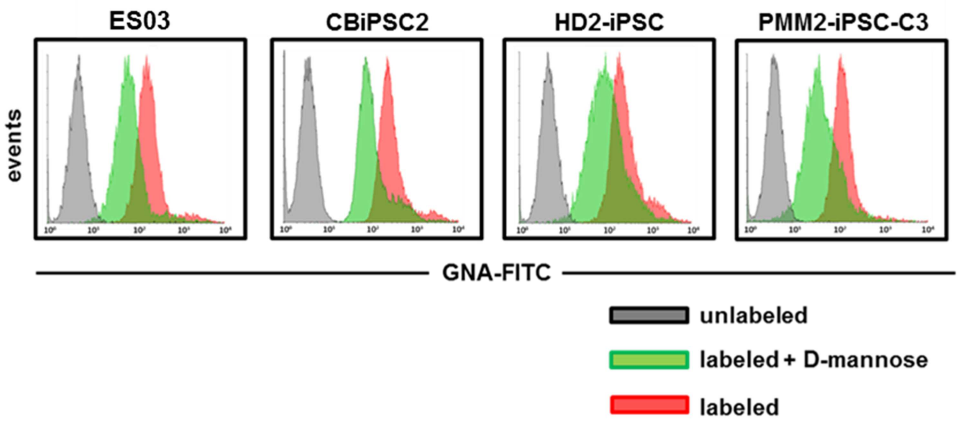

B

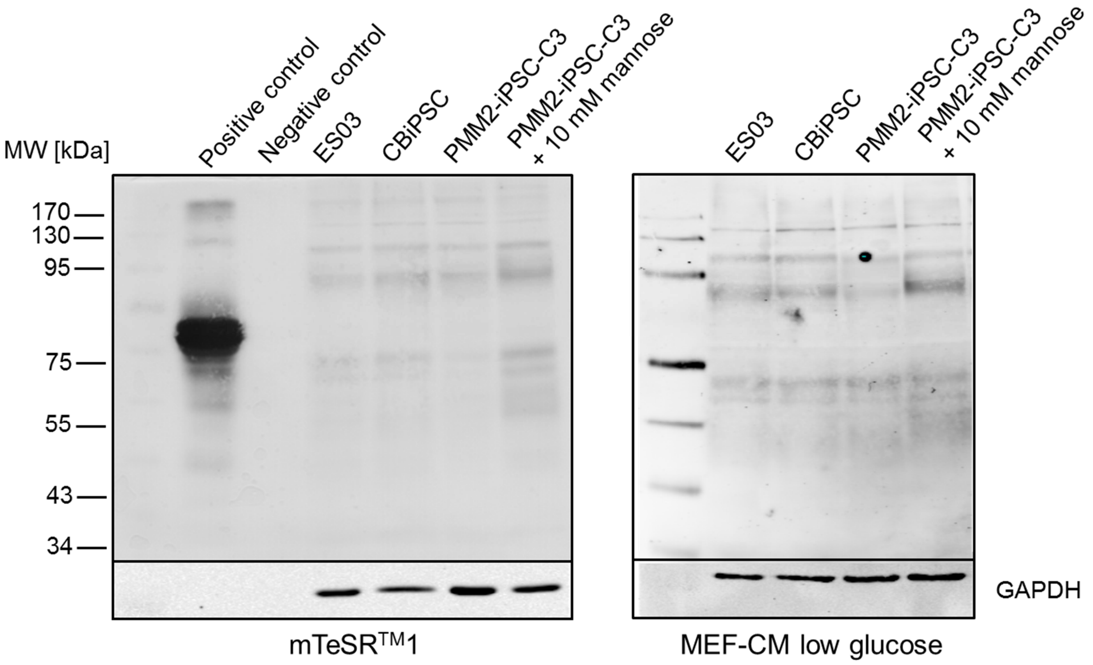

SUPPL. FIG. S1, related to Fig. 4. **Lectin analyses**. *A*, validation of GNA-FITC specificity for mannosylated glycans during flow cytometry. Histograms show representative data for ES03, CBiPSC2, HD2-iPSC, and PMM2-iPSC-C3 of flow cytometry for unlabeled (no lectin, grey), labelled (with GNA-FITC, red) and control (GNA-FITC plus 1M D-(+)-mannose during staining procedure, green). *B*, ES03, CBiPSC2, PMM2-iPSC-C3 and PMM2-iPSC-C3 +10 mM mannose (grown for three days in the presence of 10 mM mannose) were cultured on

Matrigel^{TM} in mTeSR^{TM}1 (left) or on Matrigel^{TM} in low glucose MEF-CM (right). Upon precipitation of proteins from whole cell lysates, proteins were separated by SDS-PAGE, blotted onto nitrocellulose, probed with the DIG-labeled lectin *Galantus nivalis* agglutinin (GNA) and developed with the DIG Glycan Differentiation Kit (Roche). CPDY: carboxypeptidase carboxypeptidase Y (positive control), BSA: bovine bovine serum albumin (negative control). Representative example of three independent repeats.

Sialidase S = α(2-3) Sialidase Figure S2

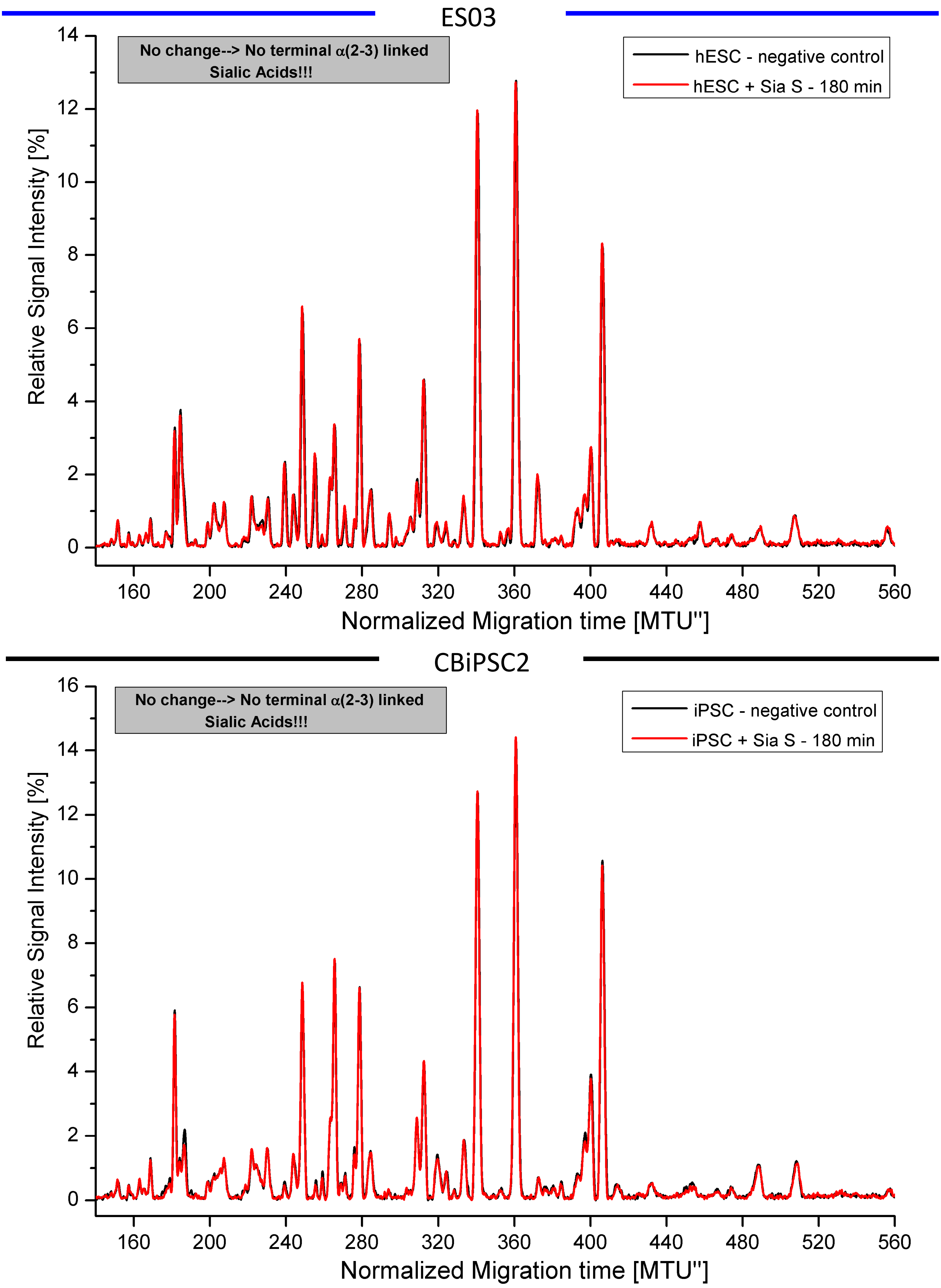

PMM2-iPSC-C3

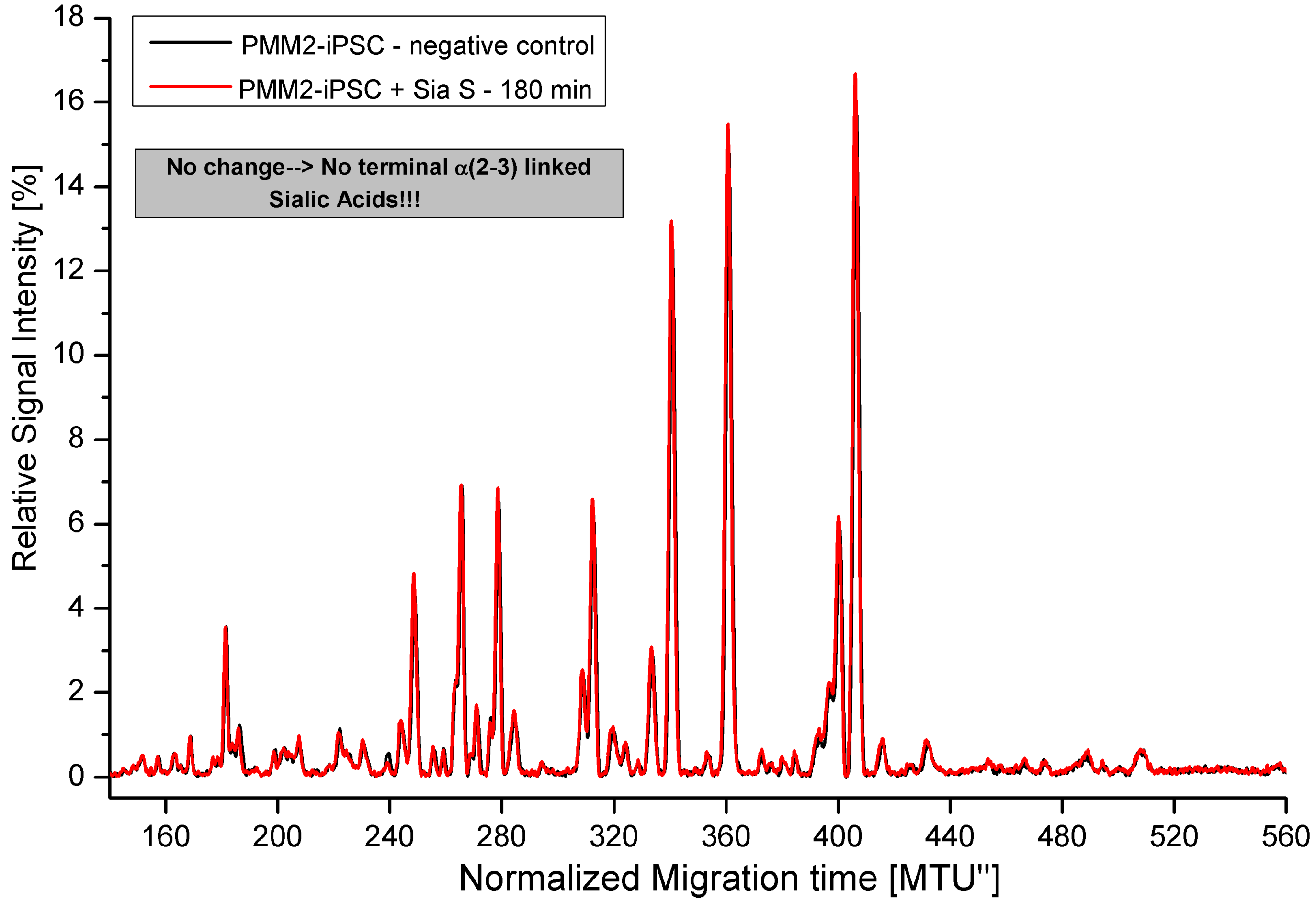

Positive Control

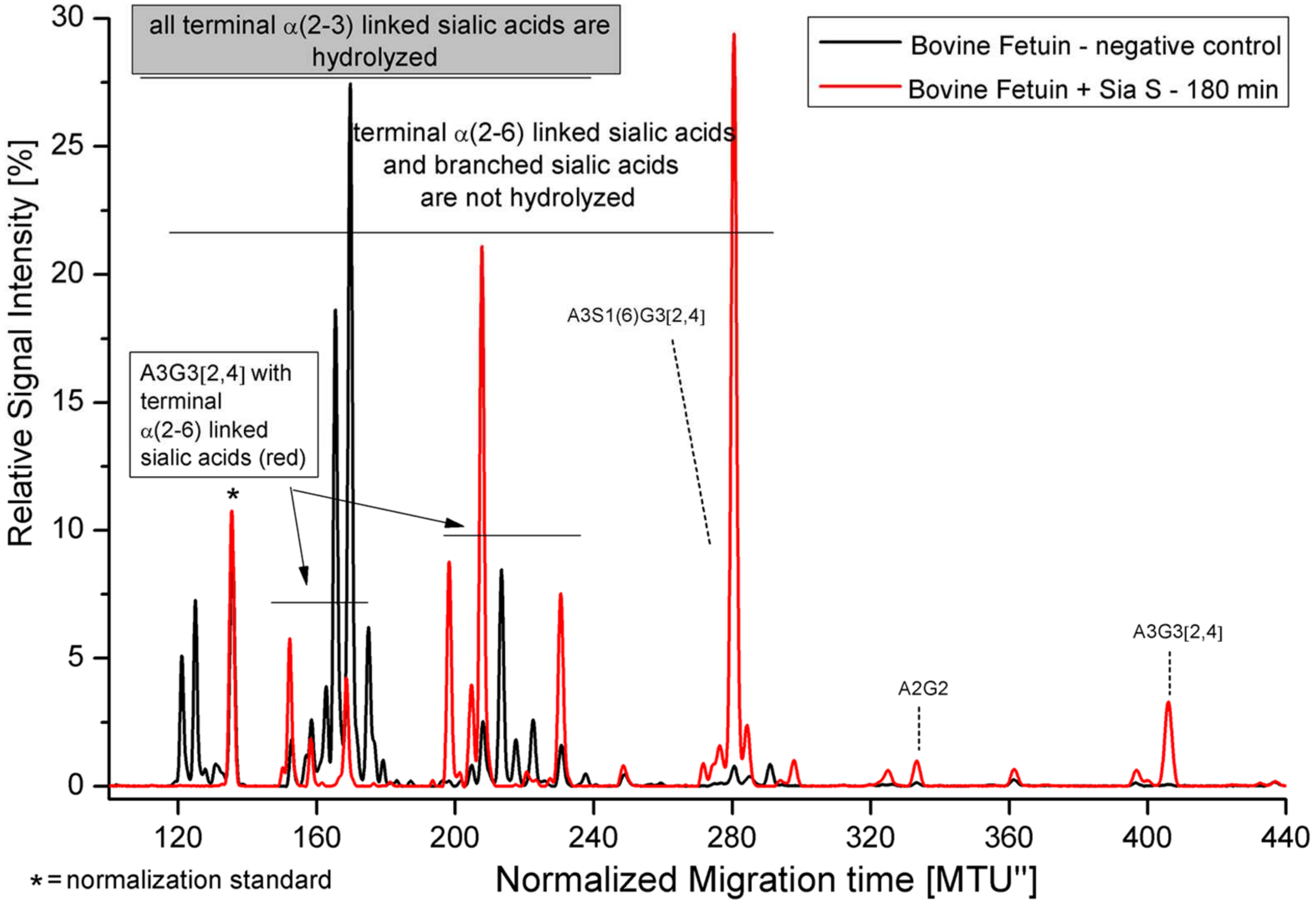

Sialidase A = α(2-3,6,8) Sialidase

ES03

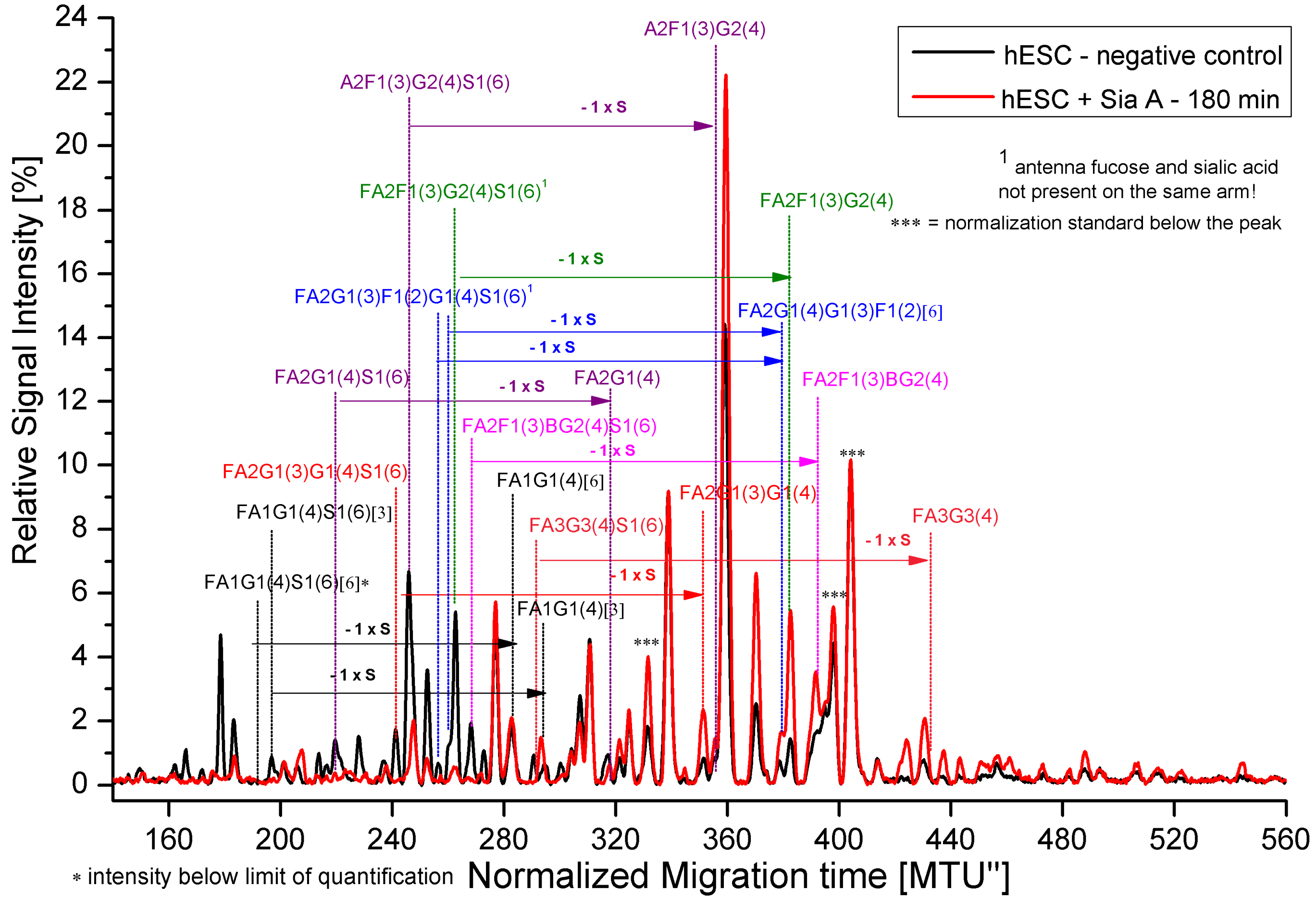

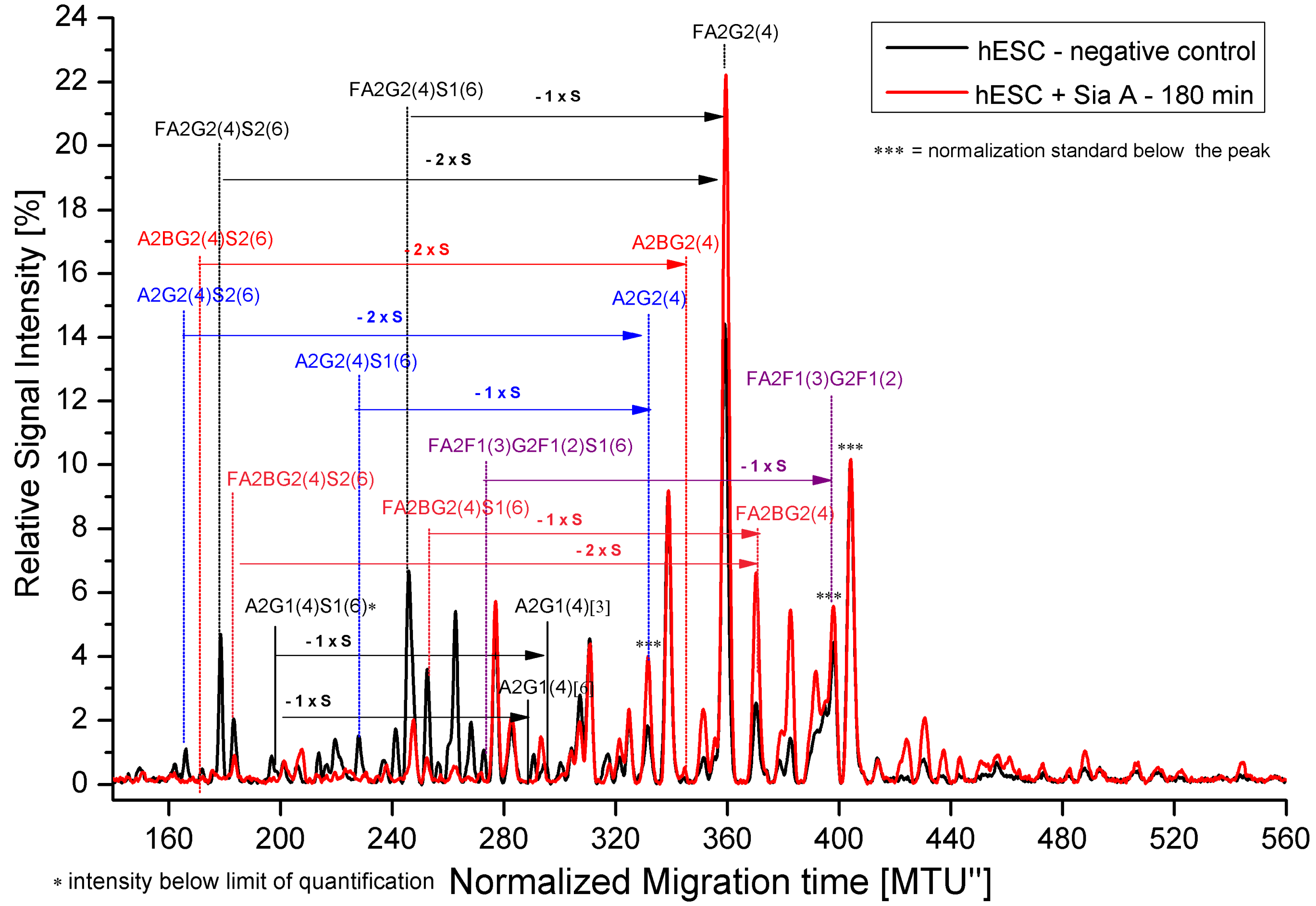

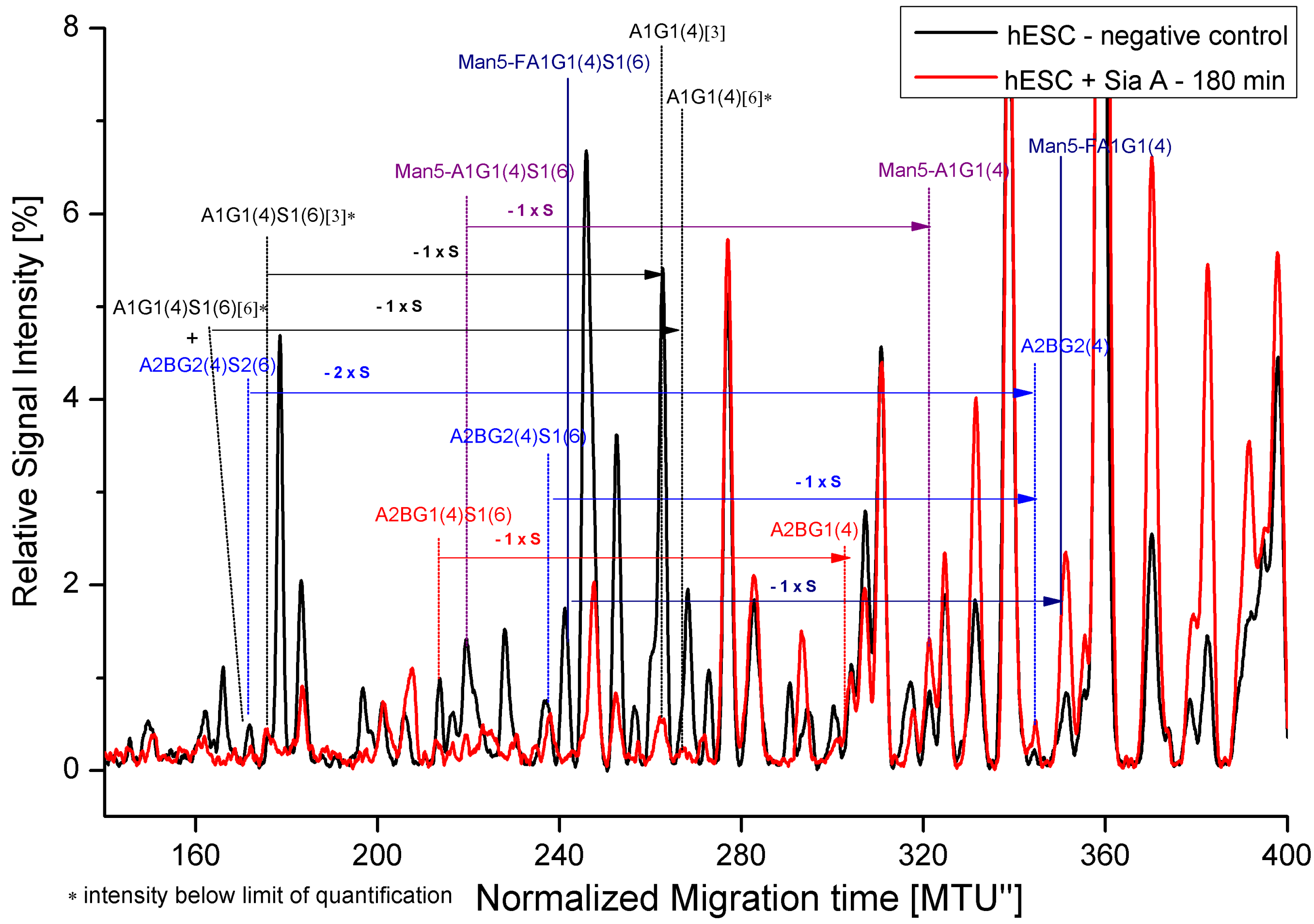
hESC - negative control
hESC + Sia A - 180 min
Relative Signal Intensity [%]
Normalized Migration time [MTU'']
* intensity below limit of quantification
A1G1(4)[3]
Man5-FA1G1(4)S1(6)
A1G1(4)[6]*
Man5-A1G1(4)S1(6)
Man5-A1G1(4)
Man5-FA1G1(4)
A1G1(4)S1(6)[3]*
A1G1(4)S1(6)[6]*
+
A2BG2(4)S2(6)
A2BG2(4)
A2BG2(4)S1(6)
A2BG1(4)S1(6)
A2BG1(4)
- 1 x S
- 2 x S

CBiPSC2

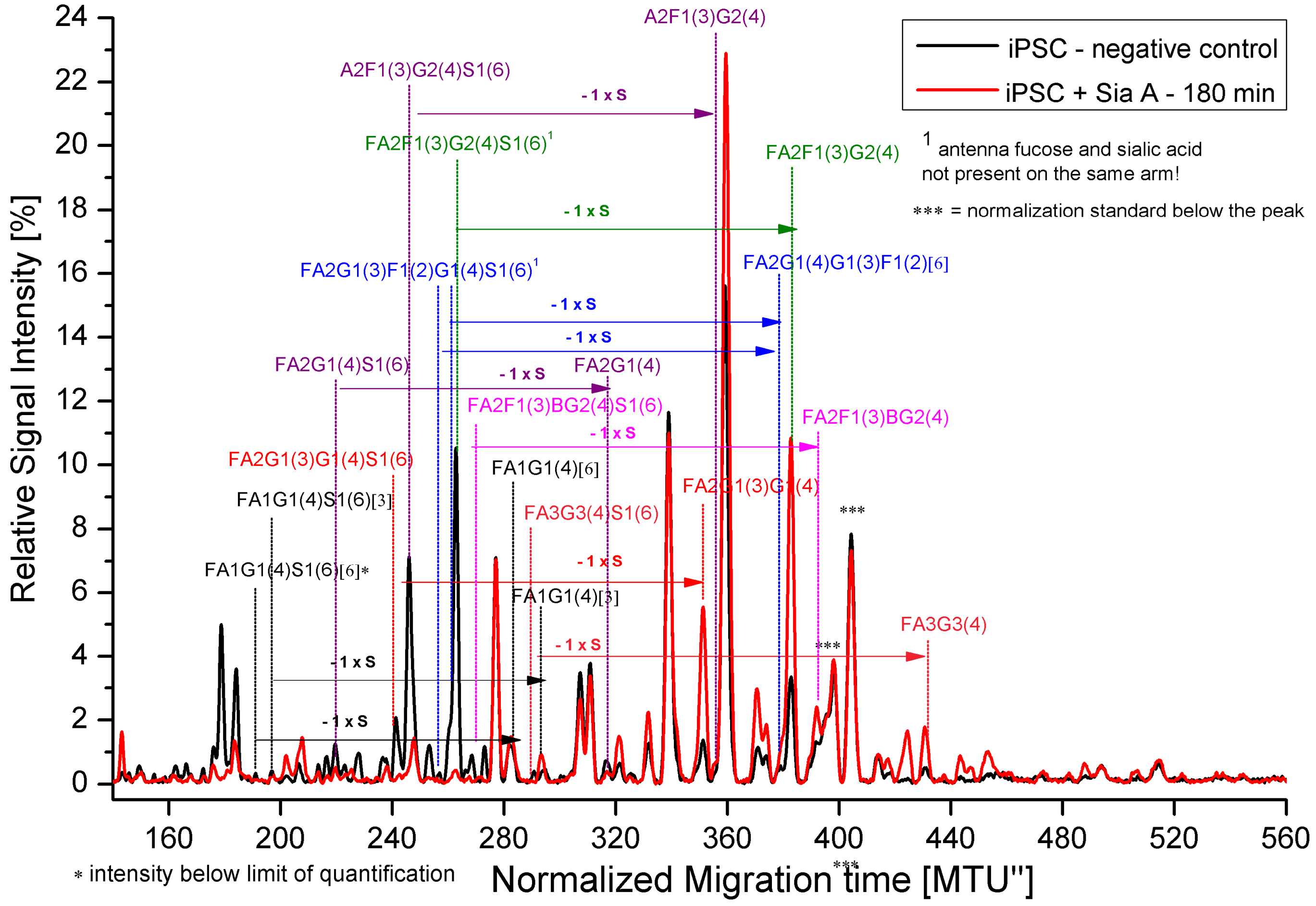
iPSC - negative control
iPSC + Sia A - 180 min
1 antenna fucose and sialic acid not present on the same arm!
*** = normalization standard below the peak
Relative Signal Intensity [%]
Normalized Migration time [MTU'']
* intensity below limit of quantification
A2F1(3)G2(4)
A2F1(3)G2(4)S1(6)
FA2F1(3)G2(4)S1(6)1
FA2F1(3)G2(4)
FA2G1(3)F1(2)G1(4)S1(6)1
FA2G1(4)G1(3)F1(2)[6]
FA2G1(4)S1(6)
FA2G1(4)
FA2F1(3)BG2(4)S1(6)
FA2F1(3)BG2(4)
FA2G1(3)G1(4)S1(6)
FA2G1(3)G1(4)
FA1G1(4)[6]
FA1G1(4)S1(6)[3]
FA3G3(4)S1(6)
FA3G3(4)
FA1G1(4)S1(6)[6]*
FA1G1(4)[3]
- 1 x S
***

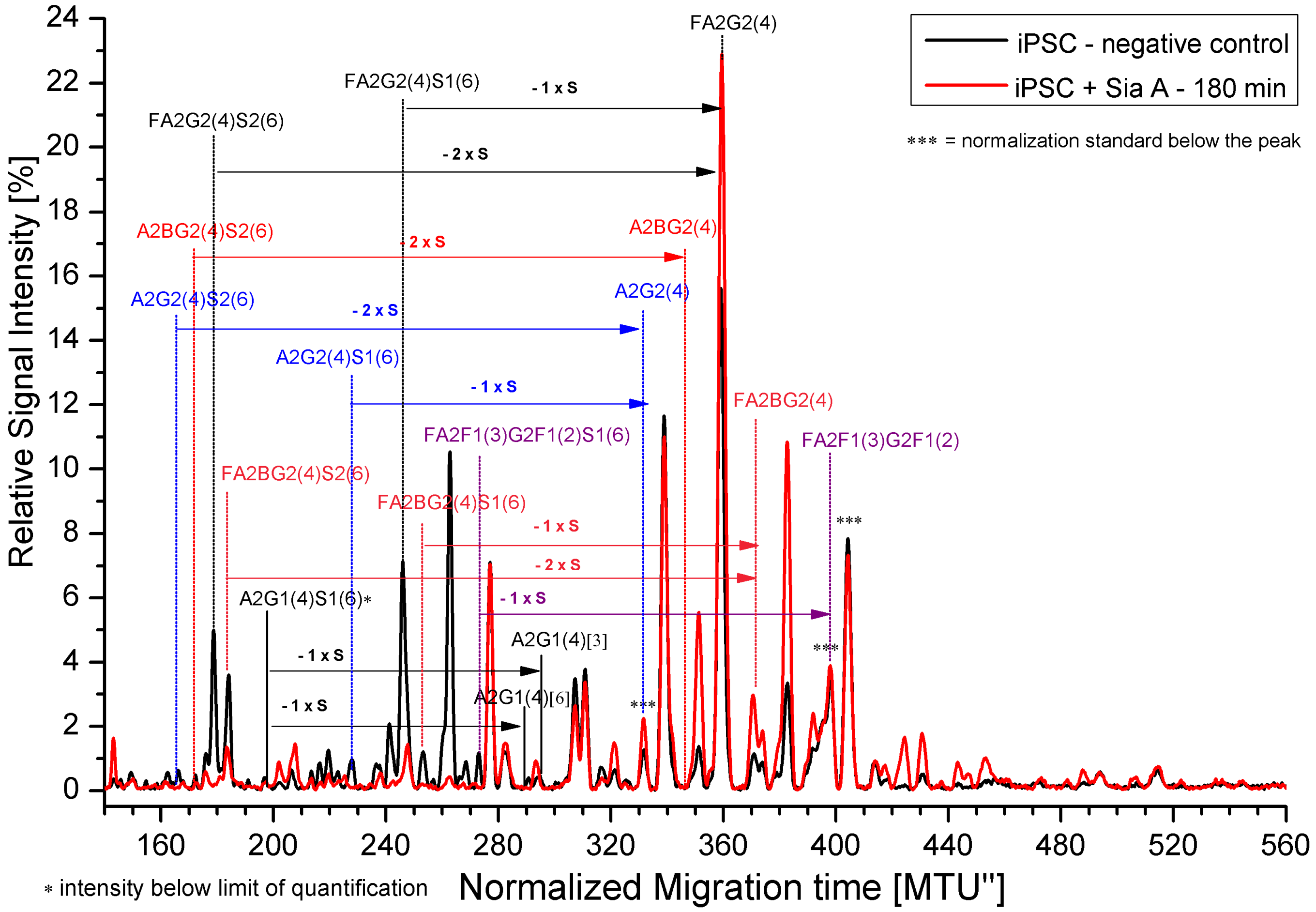

iPSC - negative control
iPSC + Sia A - 180 min
*** = normalization standard below the peak
FA2G2(4)
FA2G2(4)S1(6)
FA2G2(4)S2(6)
A2BG2(4)S2(6)
A2BG2(4)
A2G2(4)S2(6)
A2G2(4)
A2G2(4)S1(6)
FA2BG2(4)
FA2F1(3)G2F1(2)S1(6)
FA2F1(3)G2F1(2)
FA2BG2(4)S2(6)
FA2BG2(4)S1(6)
A2G1(4)S1(6)*
A2G1(4)[3]
A2G1(4)[6]
- 1 x S
- 2 x S
***
Relative Signal Intensity [%]
Normalized Migration time [MTU'']
* intensity below limit of quantification

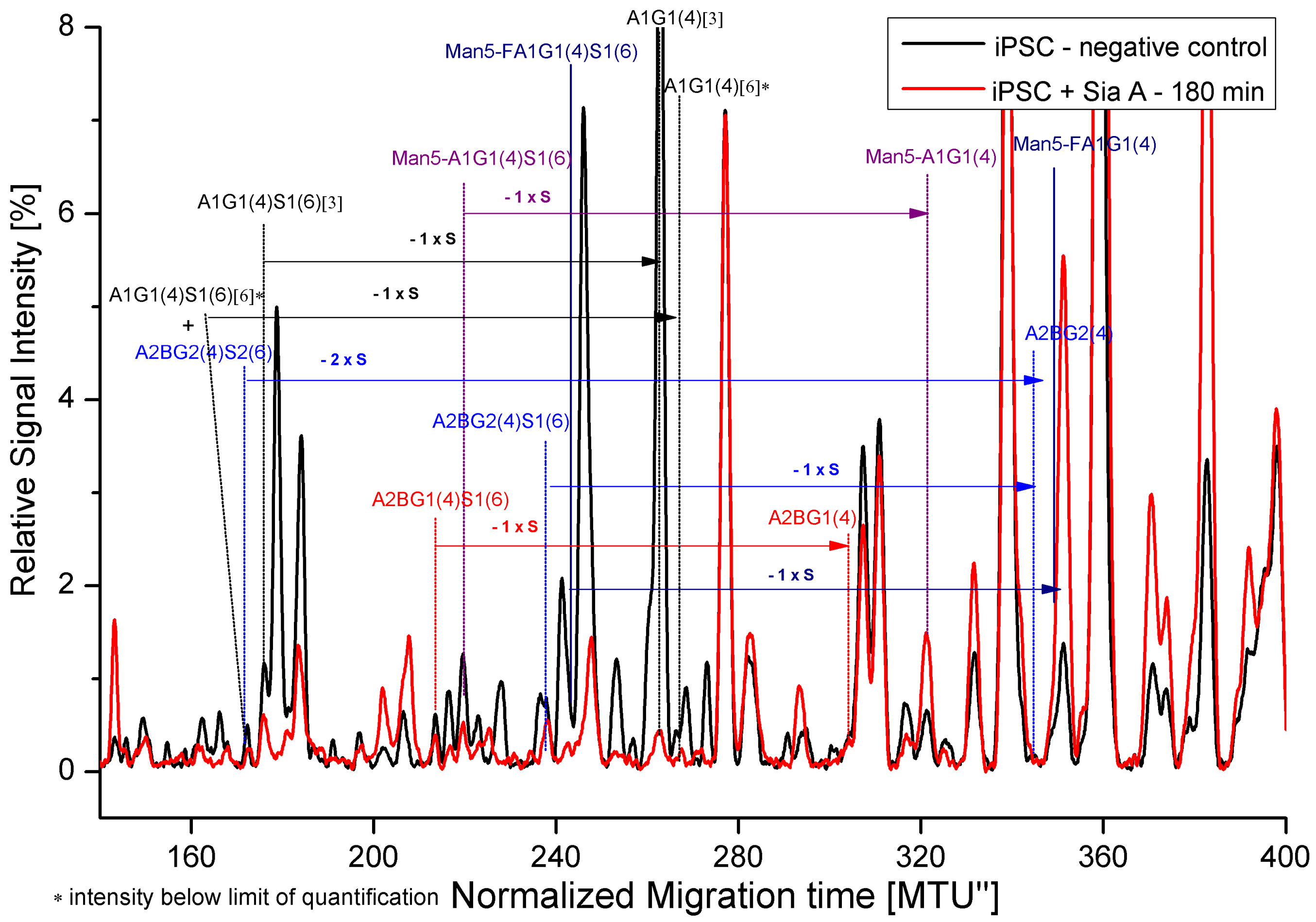

iPSC - negative control
iPSC + Sia A - 180 min
A1G1(4)[3]
Man5-FA1G1(4)S1(6)
A1G1(4)[6]*
Man5-A1G1(4)S1(6)
Man5-A1G1(4)
Man5-FA1G1(4)
A1G1(4)S1(6)[3]
A1G1(4)S1(6)[6]*
+
A2BG2(4)S2(6)
A2BG2(4)
A2BG2(4)S1(6)
A2BG1(4)S1(6)
A2BG1(4)
- 1 x S
- 2 x S
Relative Signal Intensity [%]
Normalized Migration time [MTU'']
* intensity below limit of quantification

PMM2-iPSC-C3

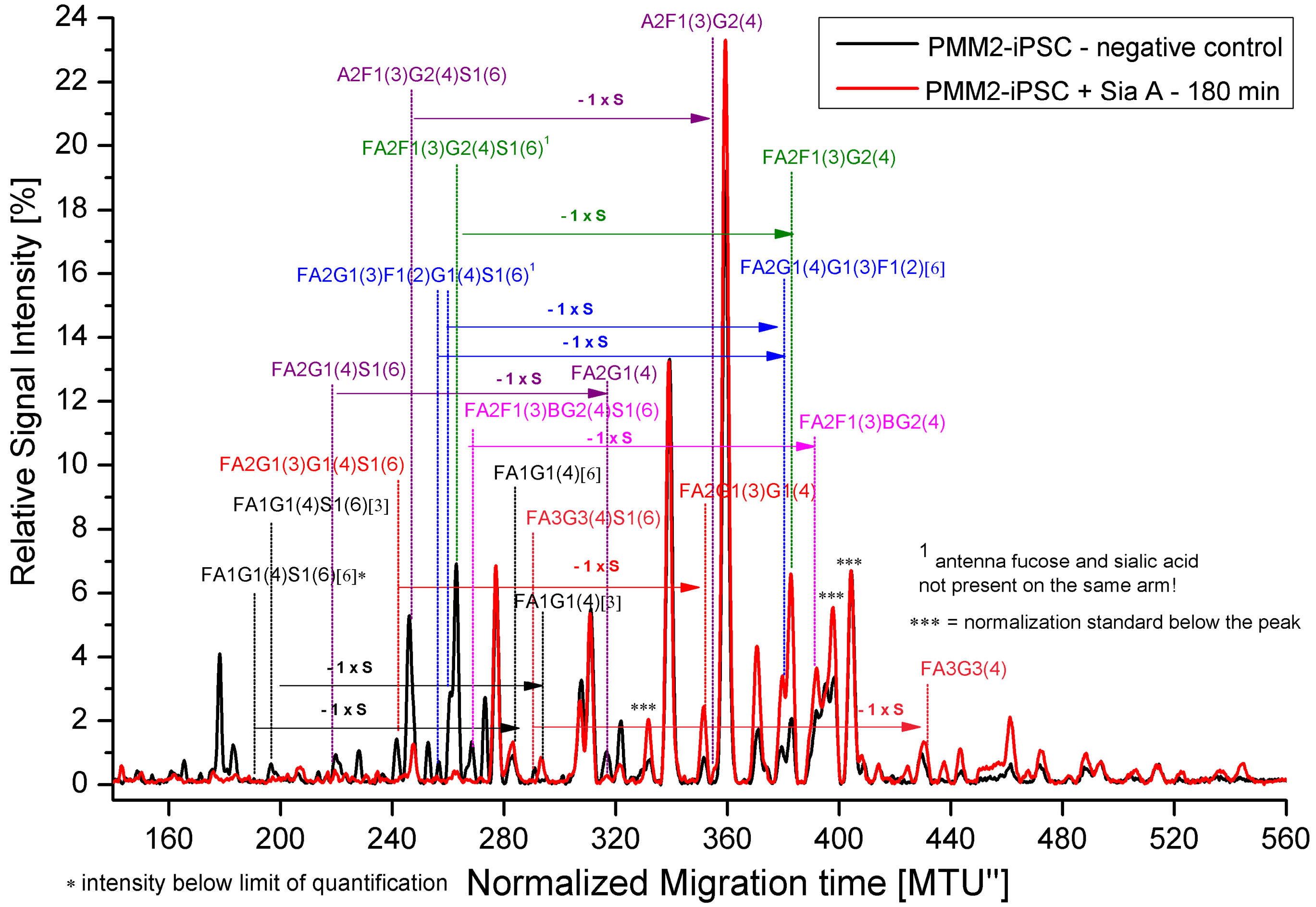

PMM2-iPSC - negative control
PMM2-iPSC + Sia A - 180 min
A2F1(3)G2(4)
A2F1(3)G2(4)S1(6)
FA2F1(3)G2(4)S1(6)1
FA2F1(3)G2(4)
FA2G1(3)F1(2)G1(4)S1(6)1
FA2G1(4)G1(3)F1(2)[6]
FA2G1(4)S1(6)
FA2G1(4)
FA2F1(3)BG2(4)S1(6)
FA2F1(3)BG2(4)
FA2G1(3)G1(4)S1(6)
FA1G1(4)[6]
FA1G1(4)S1(6)[3]
FA3G3(4)S1(6)
FA2G1(3)G1(4)
FA1G1(4)S1(6)[6]*
FA1G1(4)[3]
FA3G3(4)
- 1 x S
1 antenna fucose and sialic acid not present on the same arm!
*** = normalization standard below the peak
***
Relative Signal Intensity [%]
Normalized Migration time [MTU'']
* intensity below limit of quantification

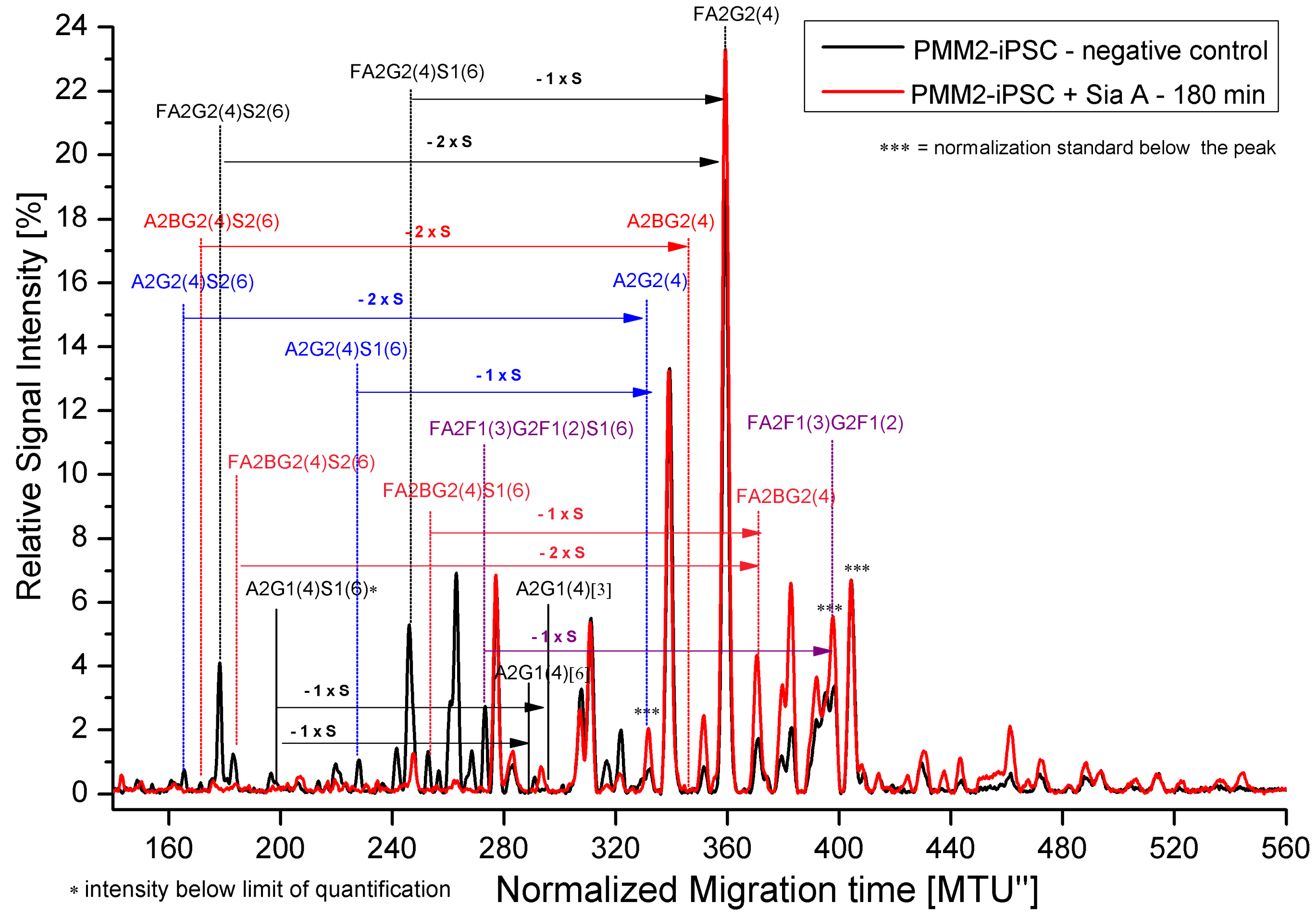

PMM2-iPSC - negative control
PMM2-iPSC + Sia A - 180 min
*** = normalization standard below the peak
FA2G2(4)
FA2G2(4)S1(6)
FA2G2(4)S2(6)
A2BG2(4)S2(6)
A2BG2(4)
A2G2(4)S2(6)
A2G2(4)
A2G2(4)S1(6)
FA2F1(3)G2F1(2)S1(6)
FA2F1(3)G2F1(2)
FA2BG2(4)S2(6)
FA2BG2(4)S1(6)
FA2BG2(4)
A2G1(4)S1(6)*
A2G1(4)[3]
A2G1(4)[6]
- 1 x S
- 2 x S
***
Relative Signal Intensity [%]
Normalized Migration time [MTU'']
* intensity below limit of quantification

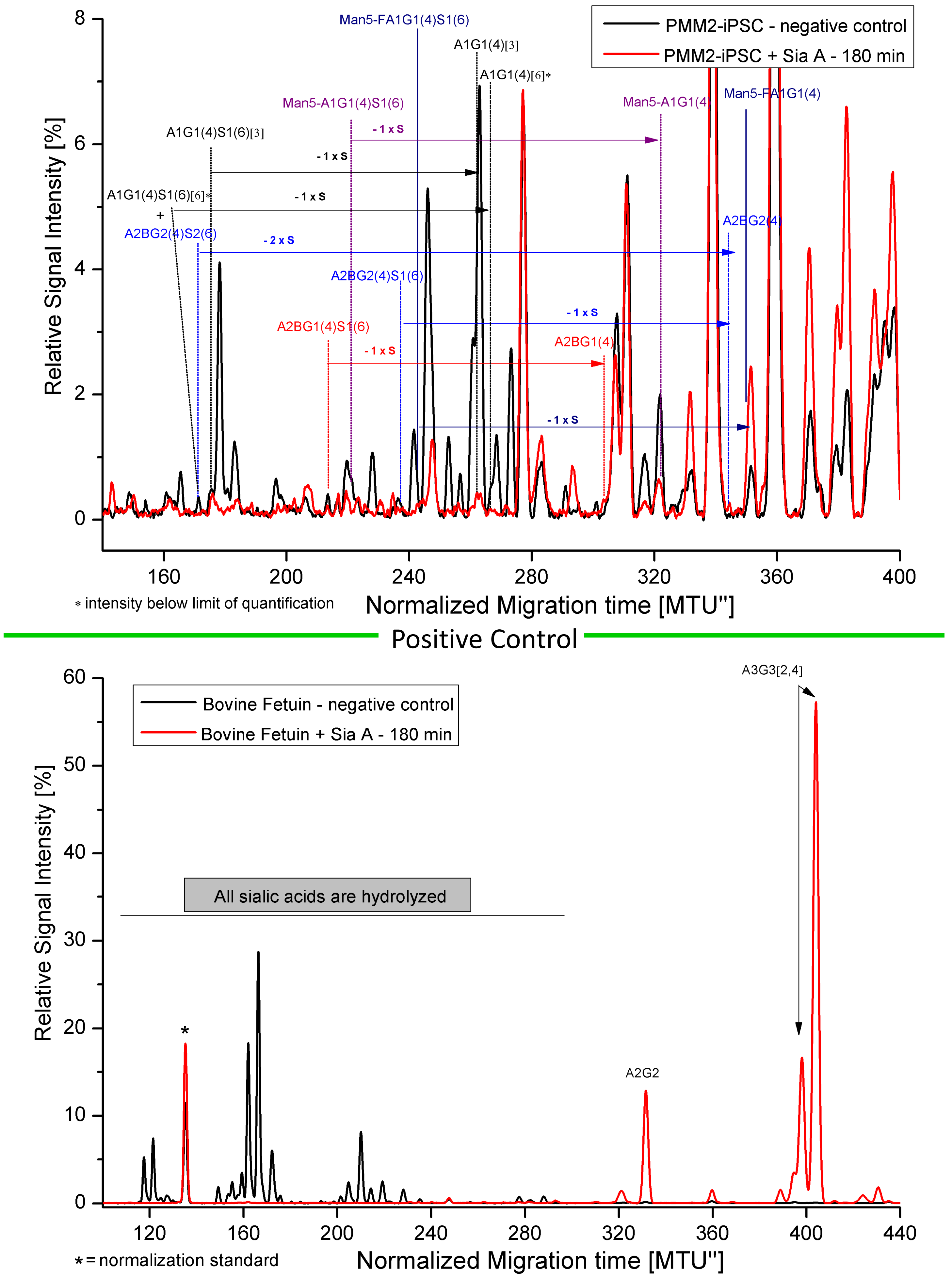
PMM2-iPSC - negative control
PMM2-iPSC + Sia A - 180 min
Relative Signal Intensity [%]
Normalized Migration time [MTU'']
* intensity below limit of quantification
Man5-FA1G1(4)S1(6)
A1G1(4)[3]
A1G1(4)[6]*
Man5-A1G1(4)S1(6)
Man5-A1G1(4)
Man5-FA1G1(4)
A1G1(4)S1(6)[3]
A1G1(4)S1(6)[6]*
+
A2BG2(4)S2(6)
A2BG2(4)
A2BG2(4)S1(6)
A2BG1(4)S1(6)
A2BG1(4)
- 1 x S
- 2 x S
Positive Control
Bovine Fetuin - negative control
Bovine Fetuin + Sia A - 180 min
All sialic acids are hydrolyzed
A3G3[2,4]
A2G2
*
* = normalization standard

α(1-3,4) Fucosidase

ES03+CBiPSC2+PMM2-iPSC-C3

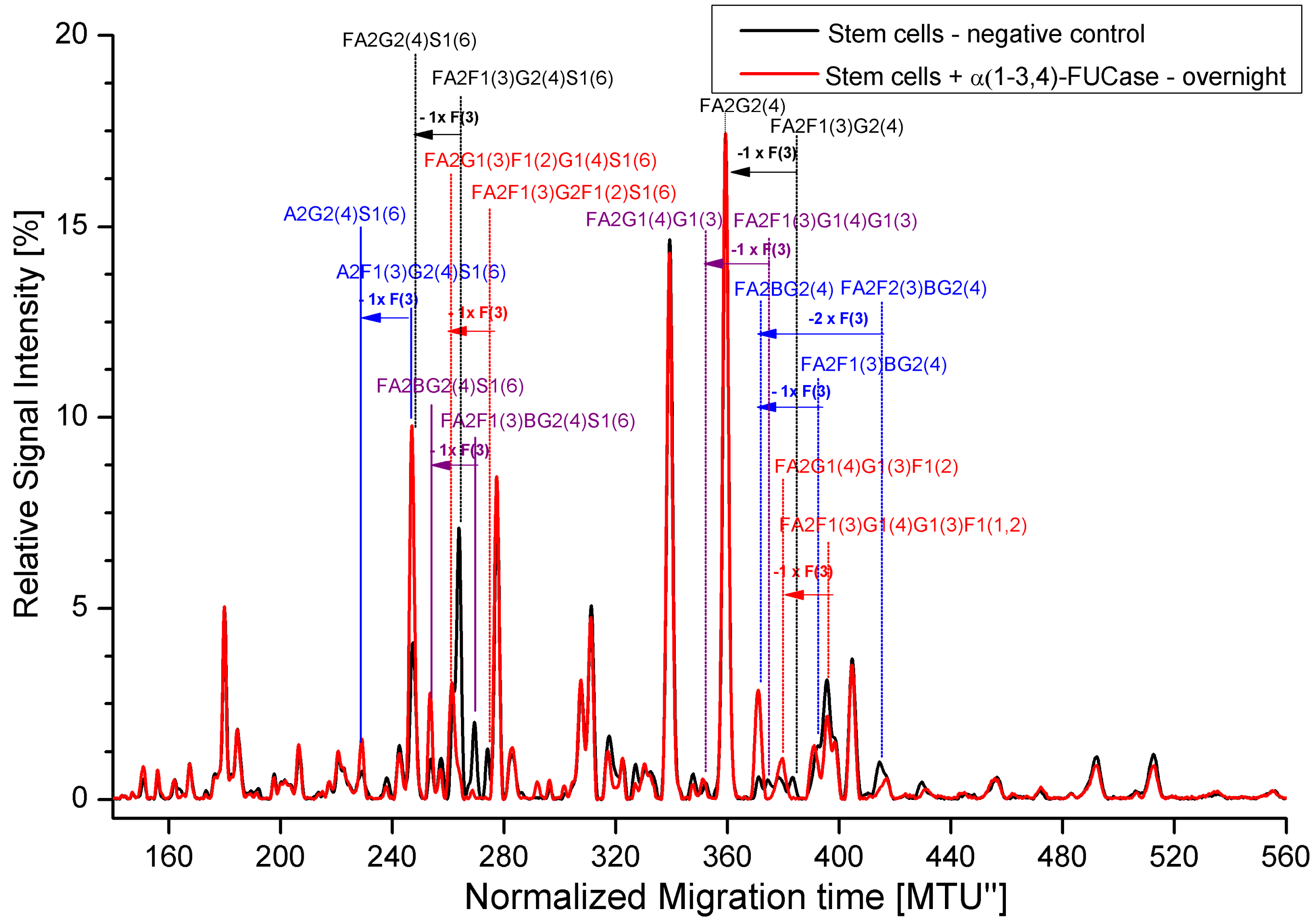

Positive Control

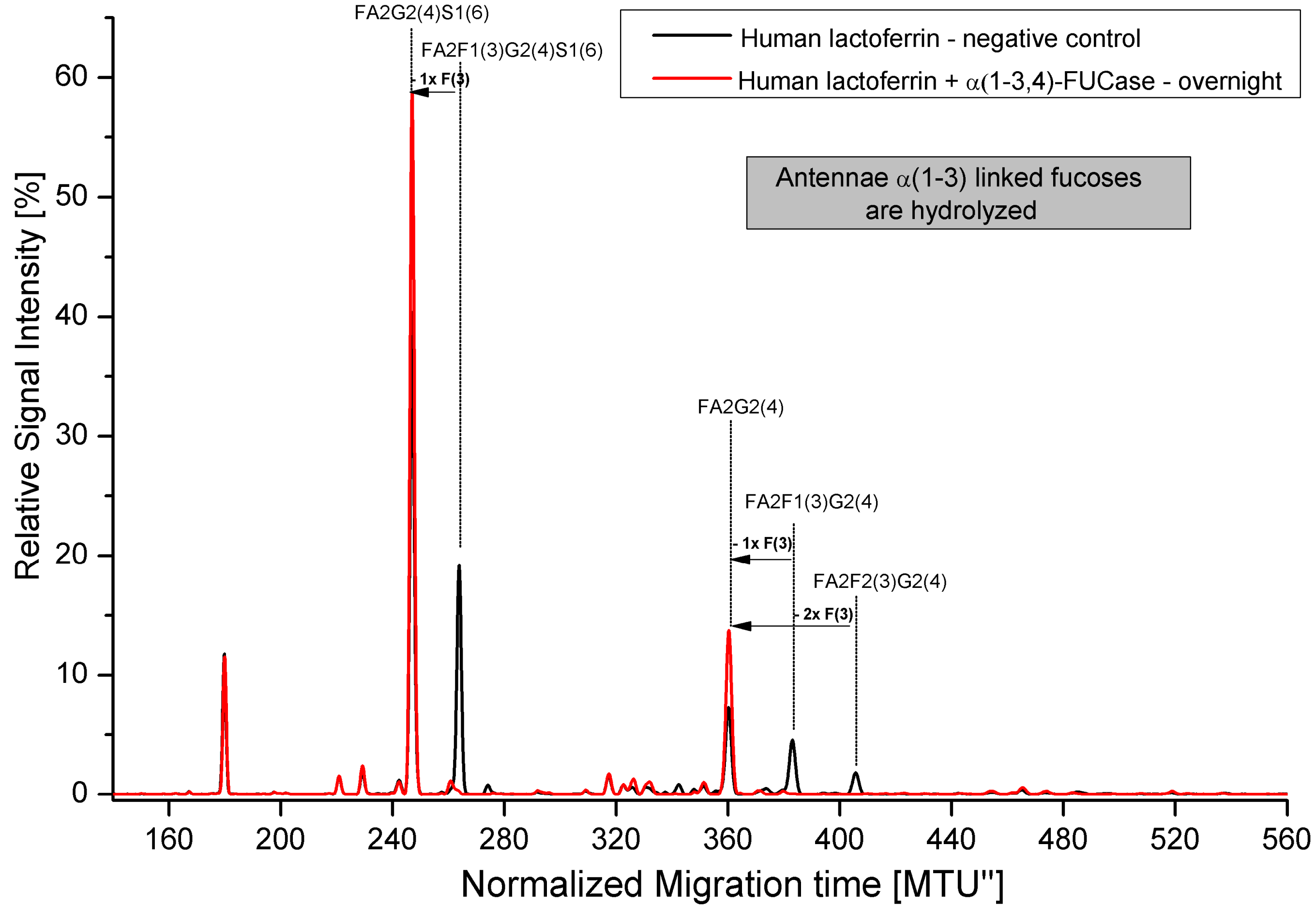

α(1-2) Fucosidase

ES03+CBiPSC2+PMM2-iPSC-C3

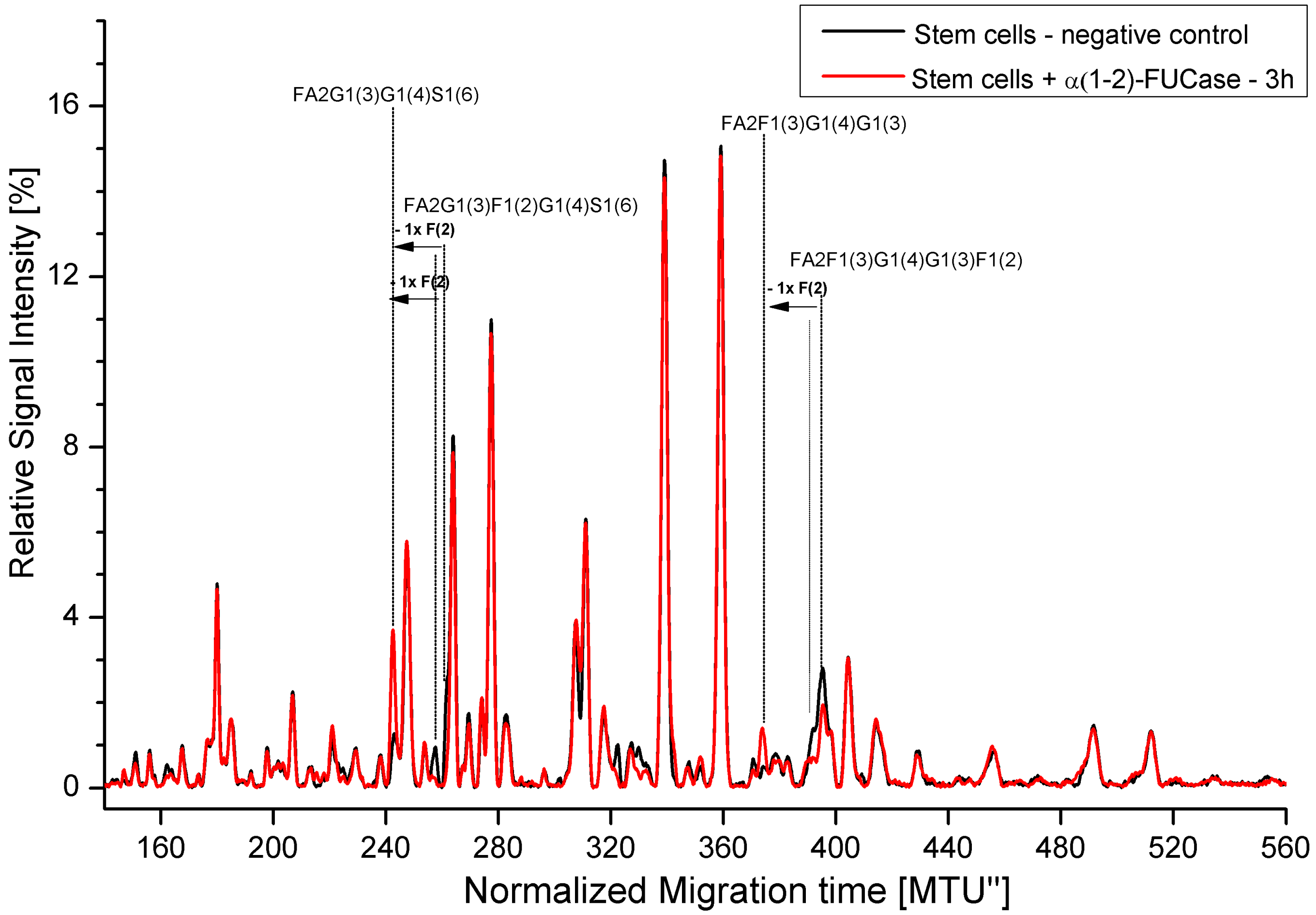

Positive Control

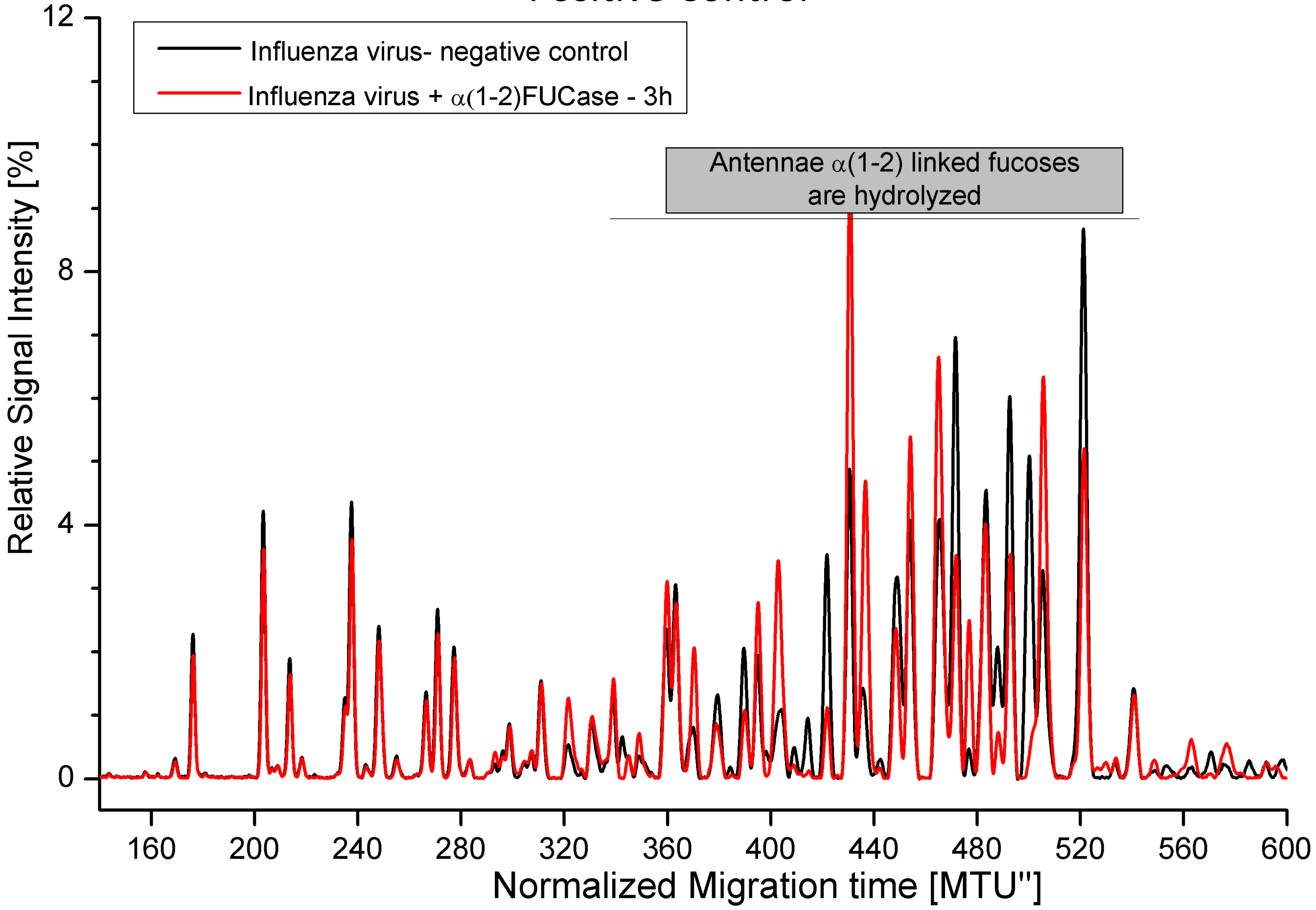

α(1-2,3,4,6) Fucosidase

ES03

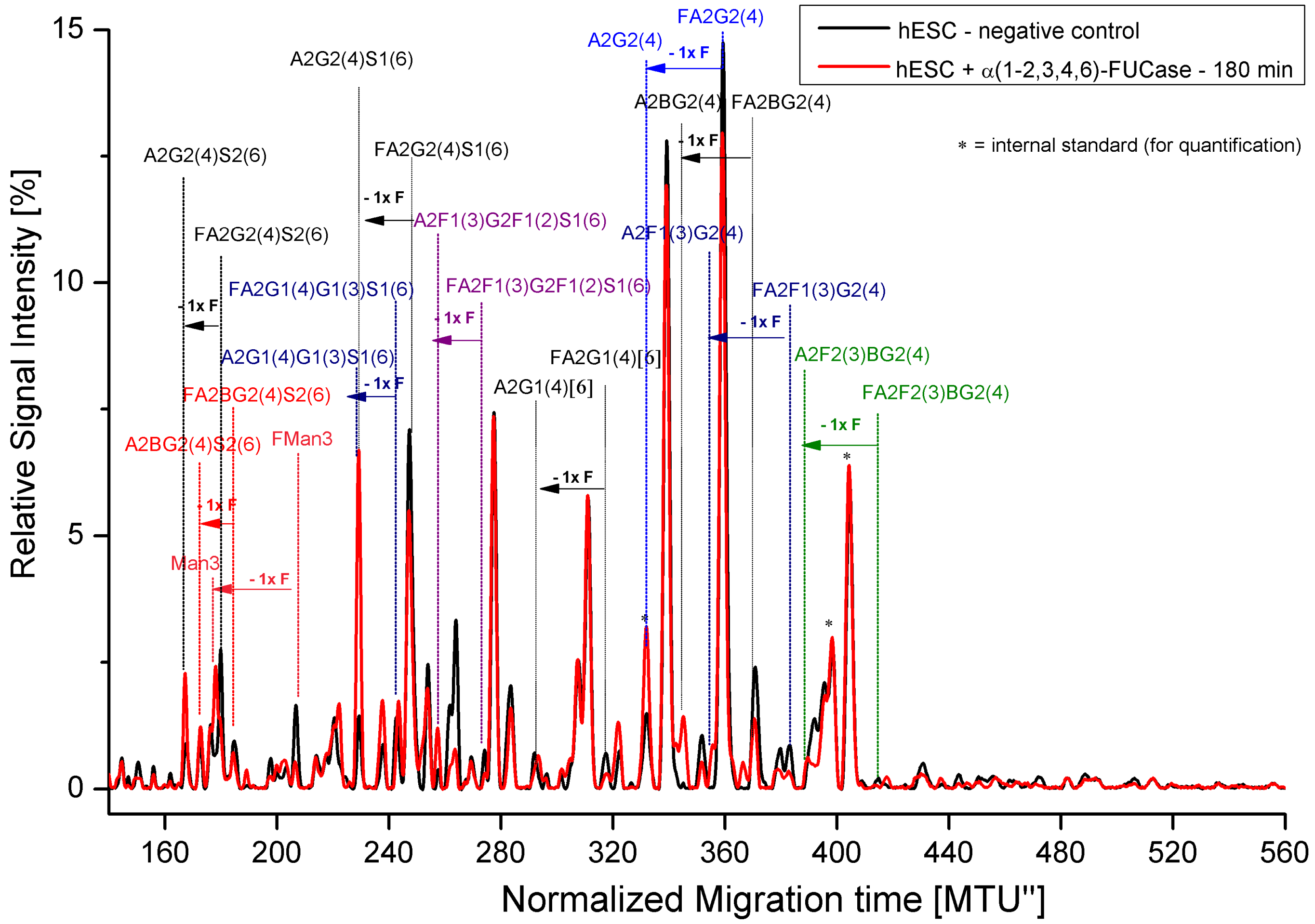

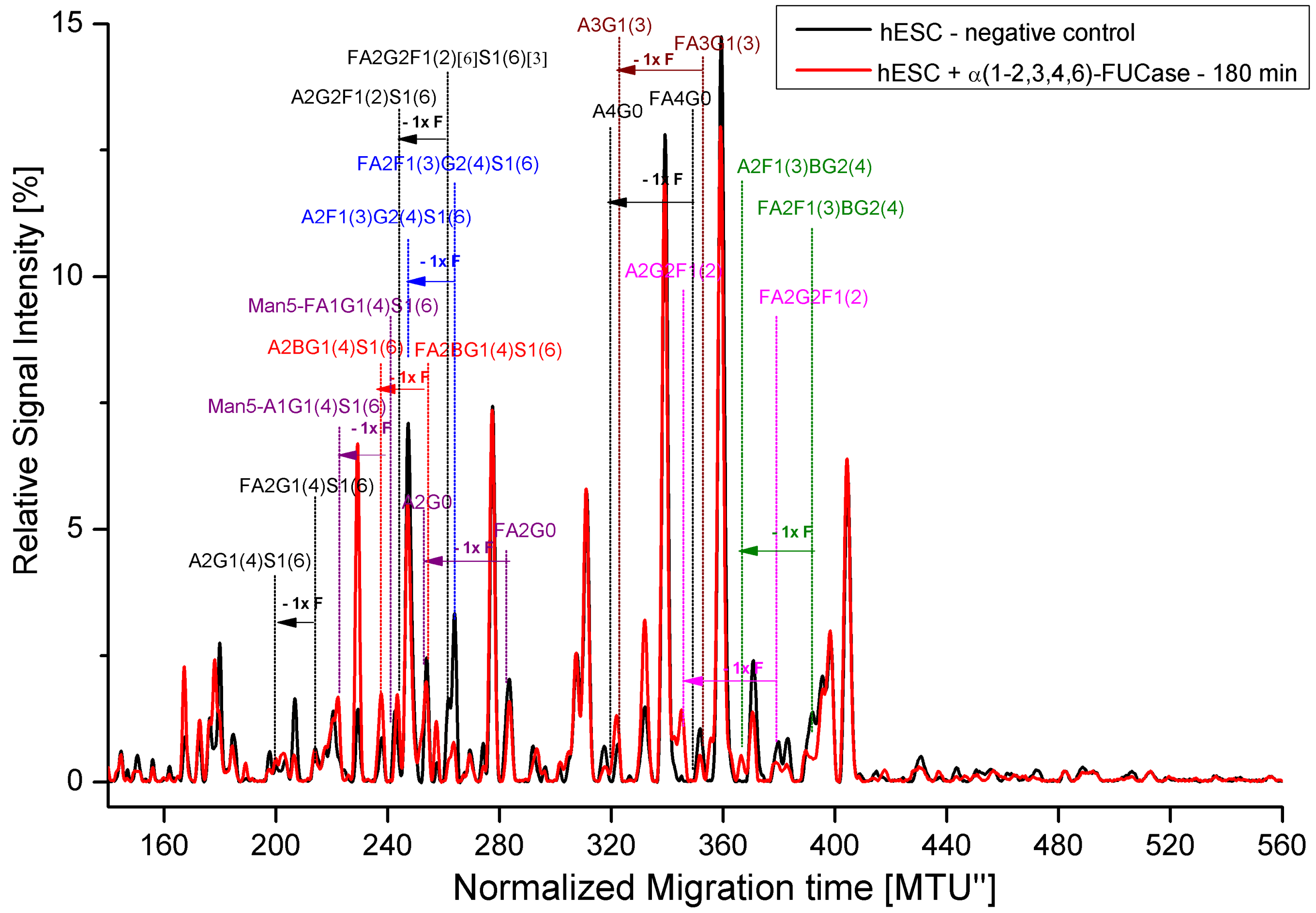

CBiPSC2

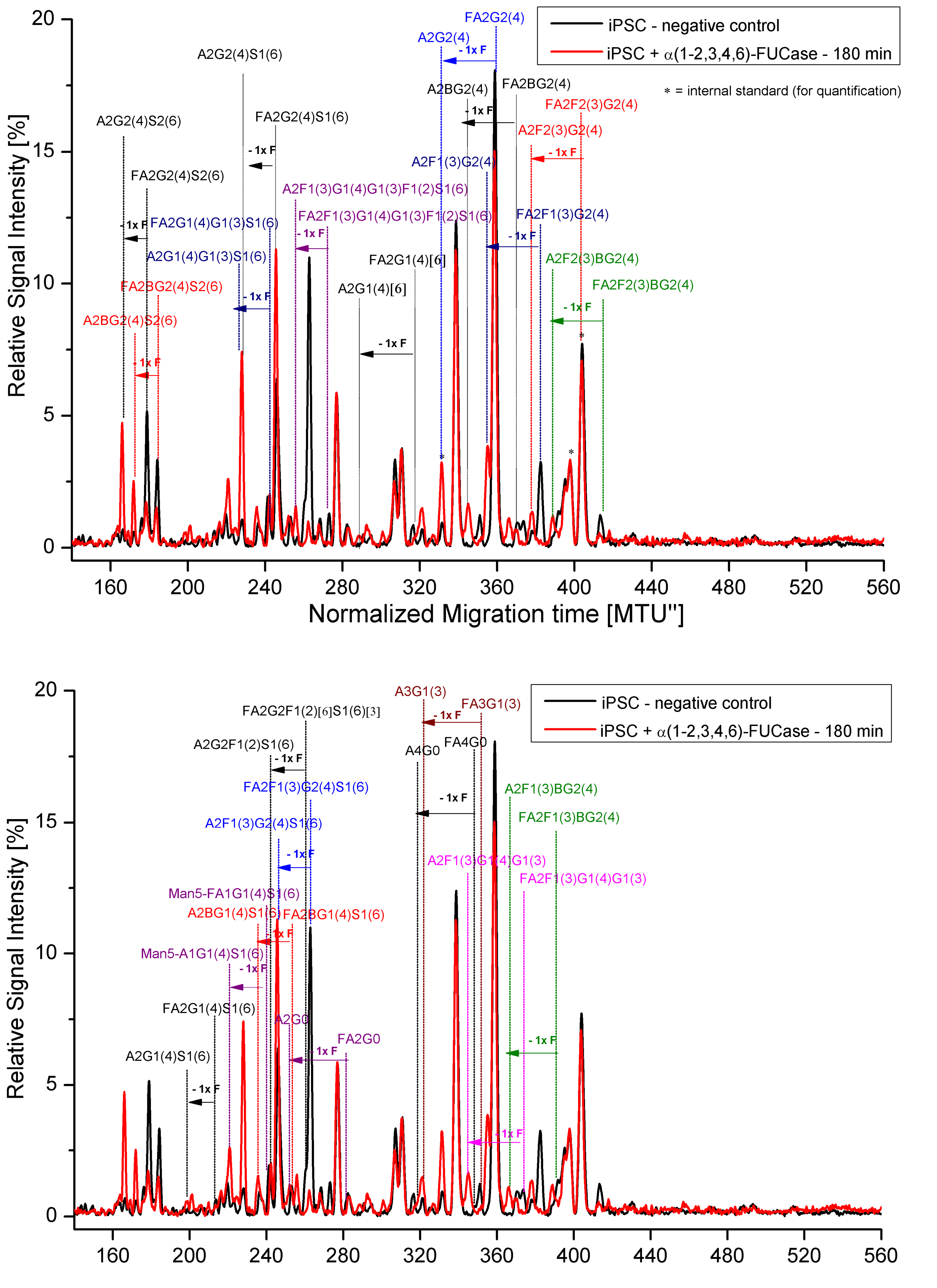

iPSC - negative control
iPSC + α(1-2,3,4,6)-FUCase - 180 min
* = internal standard (for quantification)
Relative Signal Intensity [%]
Normalized Migration time [MTU'']
A2G2(4)S2(6)
FA2G2(4)S2(6)
A2BG2(4)S2(6)
FA2BG2(4)S2(6)
A2G2(4)S1(6)
FA2G2(4)S1(6)
FA2G1(4)G1(3)S1(6)
A2G1(4)G1(3)S1(6)
A2F1(3)G1(4)G1(3)F1(2)S1(6)
FA2F1(3)G1(4)G1(3)F1(2)S1(6)
FA2G1(4)[6]
A2G1(4)[6]
FA2G2(4)
A2G2(4)
A2BG2(4)
FA2BG2(4)
FA2F2(3)G2(4)
A2F2(3)G2(4)
A2F1(3)G2(4)
FA2F1(3)G2(4)
A2F2(3)BG2(4)
FA2F2(3)BG2(4)
- 1x F
FA2G2F1(2)[6]S1(6)[3]
A2G2F1(2)S1(6)
FA2F1(3)G2(4)S1(6)
A2F1(3)G2(4)S1(6)
Man5-FA1G1(4)S1(6)
A2BG1(4)S1(6)
FA2BG1(4)S1(6)
Man5-A1G1(4)S1(6)
FA2G1(4)S1(6)
A2G1(4)S1(6)
A2G0
FA2G0
A3G1(3)
FA3G1(3)
A4G0
FA4G0
A2F1(3)BG2(4)
FA2F1(3)BG2(4)
A2F1(3)G1(4)G1(3)
FA2F1(3)G1(4)G1(3)

PMM2-iPSC-C3

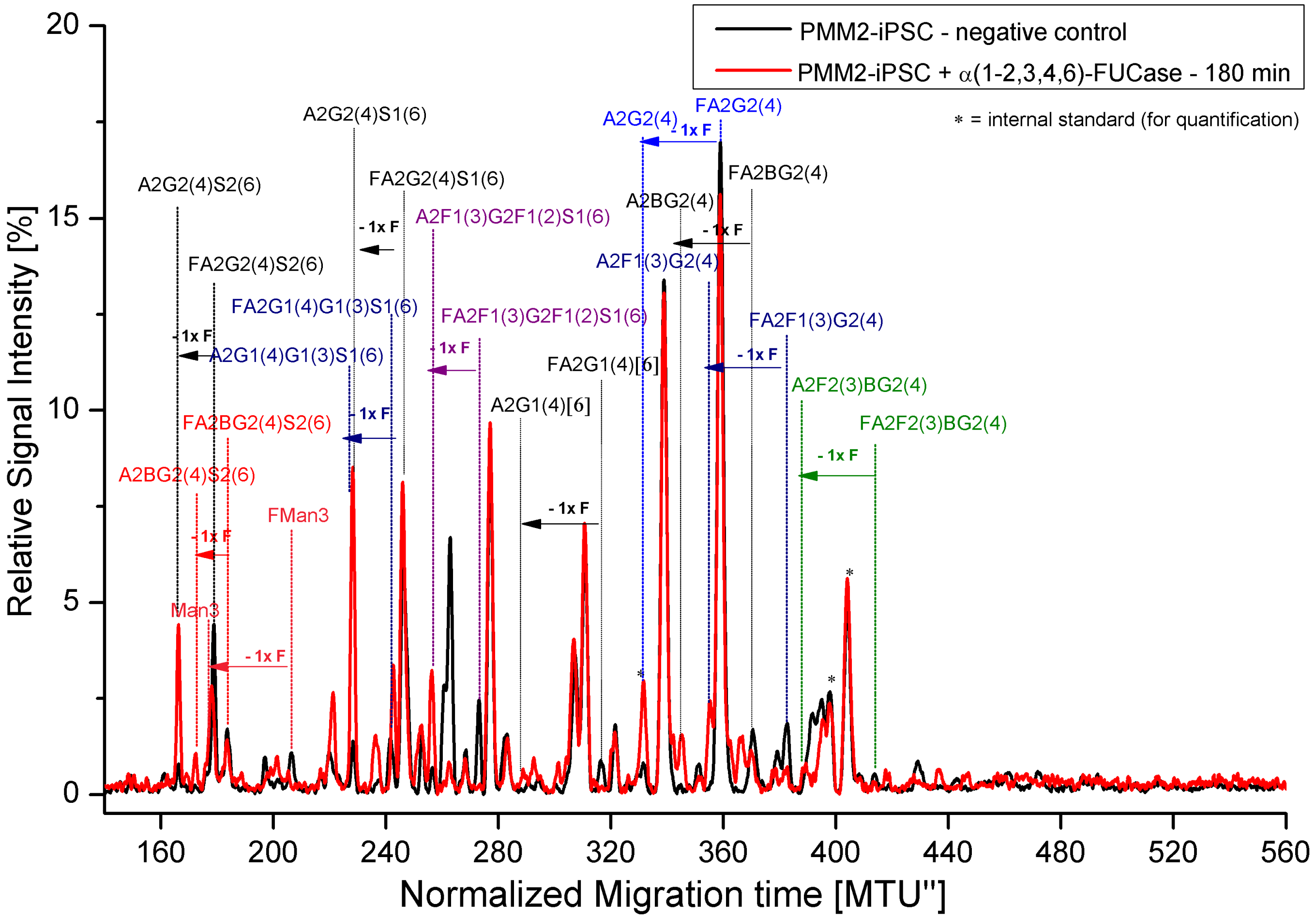

PMM2-iPSC - negative control
PMM2-iPSC + α(1-2,3,4,6)-FUCase - 180 min
* = internal standard (for quantification)
A2G2(4)S2(6)
FA2G2(4)S2(6)
A2G2(4)S1(6)
FA2G2(4)S1(6)
FA2G1(4)G1(3)S1(6)
A2G1(4)G1(3)S1(6)
A2F1(3)G2F1(2)S1(6)
FA2F1(3)G2F1(2)S1(6)
FA2BG2(4)S2(6)
A2BG2(4)S2(6)
FMan3
Man3
A2G2(4)
FA2G2(4)
A2BG2(4)
FA2BG2(4)
A2F1(3)G2(4)
FA2F1(3)G2(4)
FA2G1(4)[6]
A2G1(4)[6]
A2F2(3)BG2(4)
FA2F2(3)BG2(4)
- 1x F
Relative Signal Intensity [%]
Normalized Migration time [MTU'']

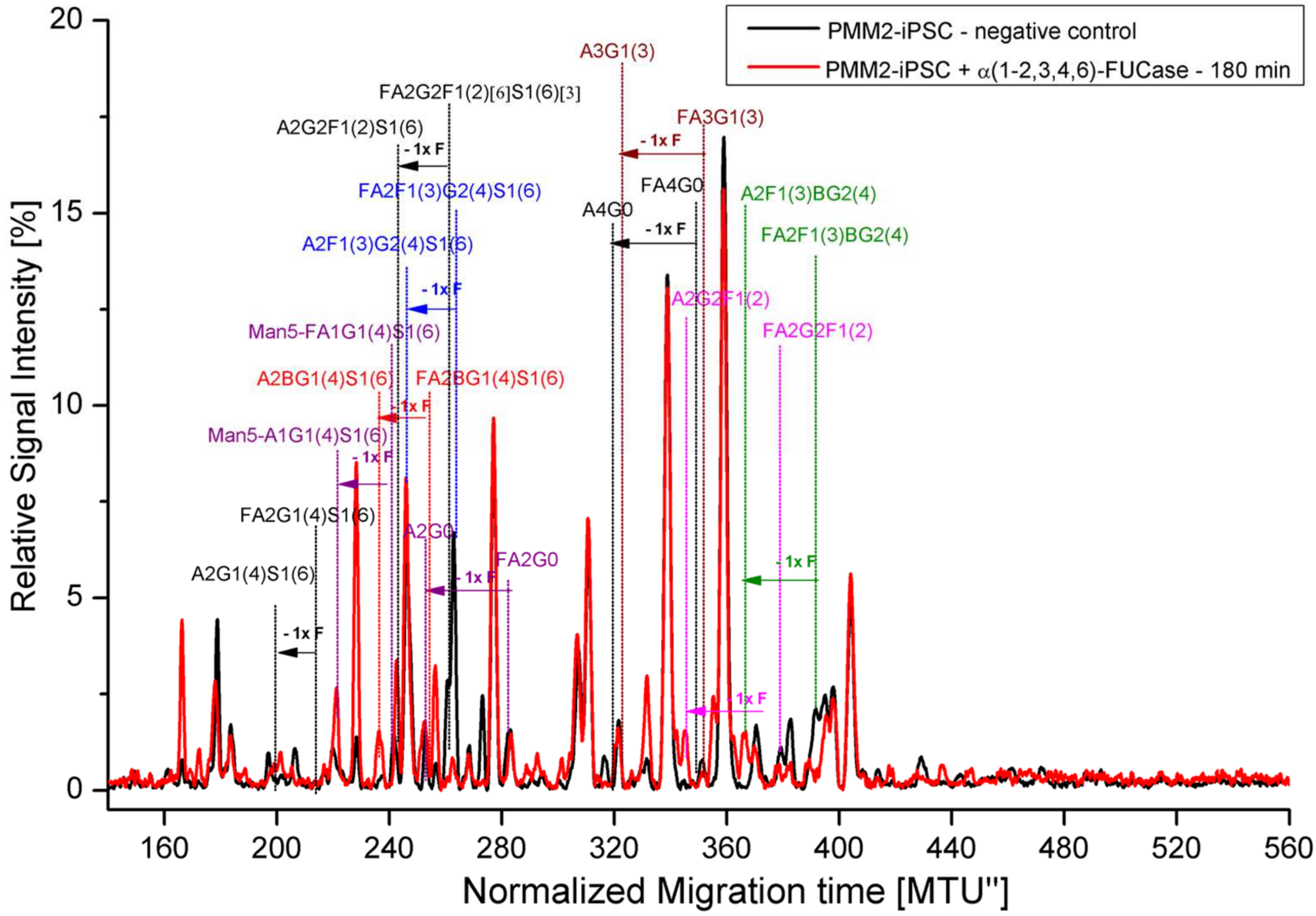

PMM2-iPSC - negative control
PMM2-iPSC + α(1-2,3,4,6)-FUCase - 180 min
A3G1(3)
FA3G1(3)
FA2G2F1(2)[6]S1(6)[3]
A2G2F1(2)S1(6)
FA2F1(3)G2(4)S1(6)
A2F1(3)G2(4)S1(6)
A4G0
FA4G0
A2F1(3)BG2(4)
FA2F1(3)BG2(4)
A2G2F1(2)
FA2G2F1(2)
Man5-FA1G1(4)S1(6)
A2BG1(4)S1(6)
FA2BG1(4)S1(6)
Man5-A1G1(4)S1(6)
FA2G1(4)S1(6)
A2G1(4)S1(6)
A2G0
FA2G0
- 1x F
Relative Signal Intensity [%]
Normalized Migration time [MTU'']

Positive Control

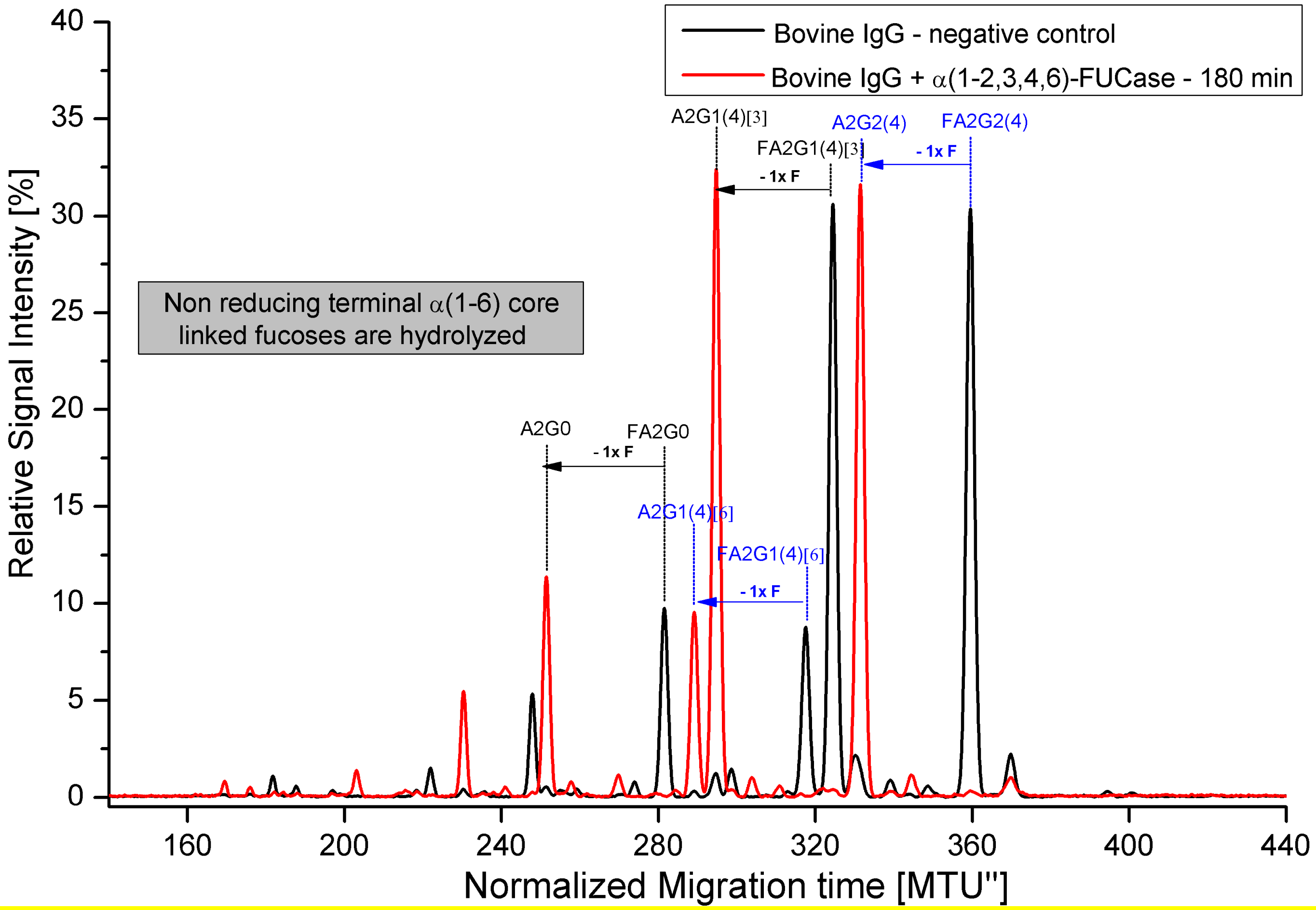

β(1-3) Galactosidase

ES03+CBiPSC2+PMM2-iPSC-C3

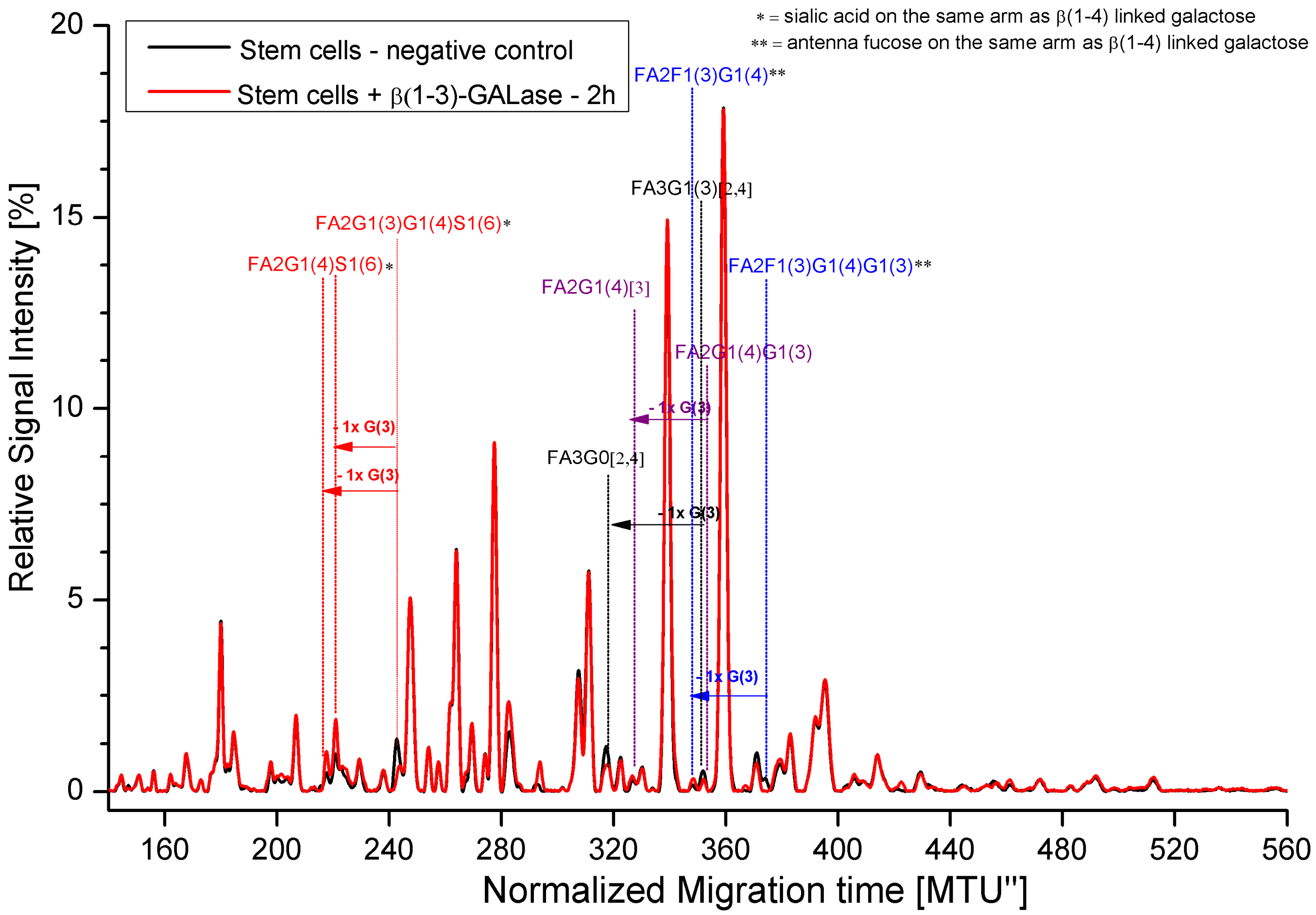

Positive Control

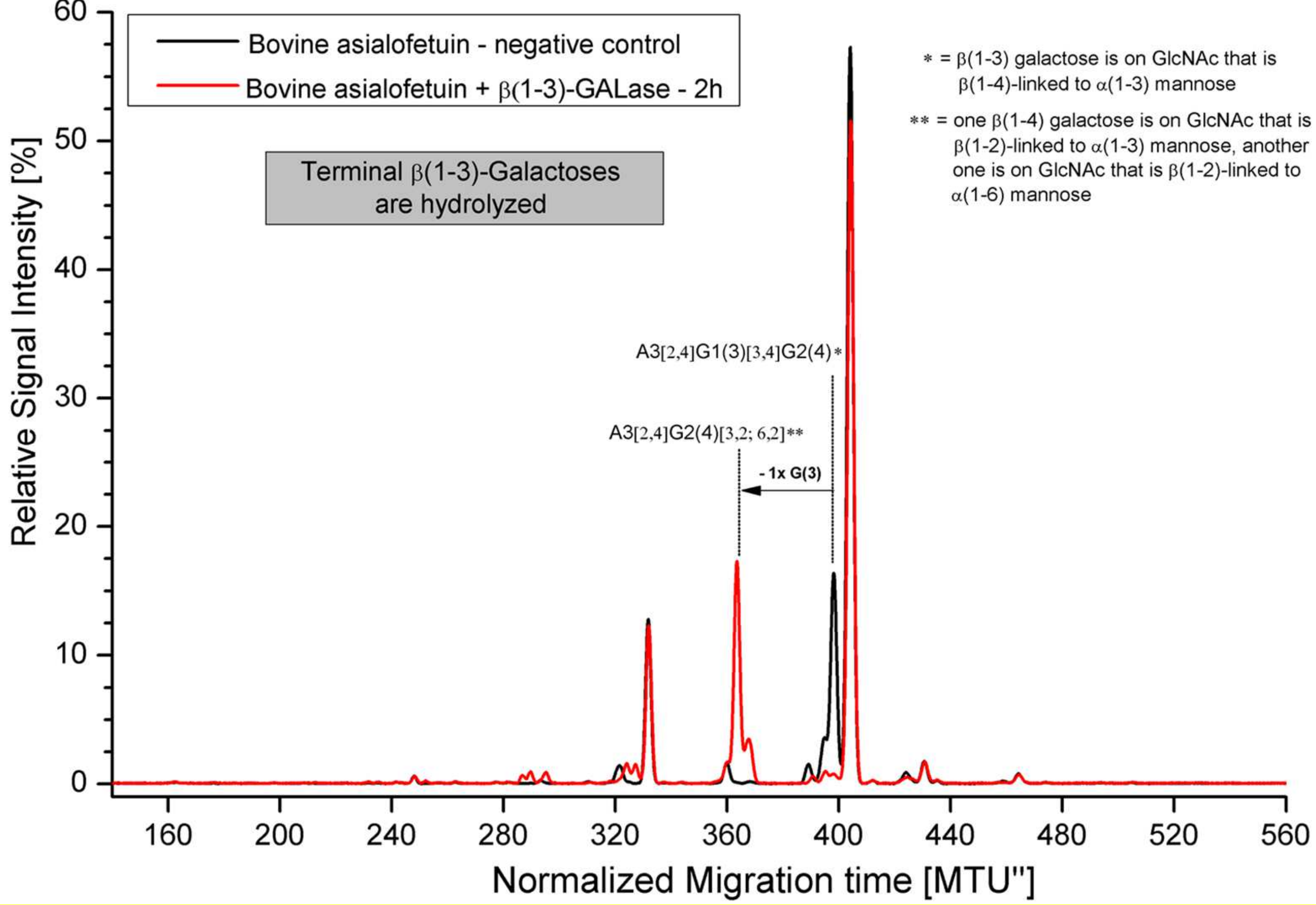

β(1-4) Galactosidase

ES03+CBiPSC2+PMM2-iPSC-C3

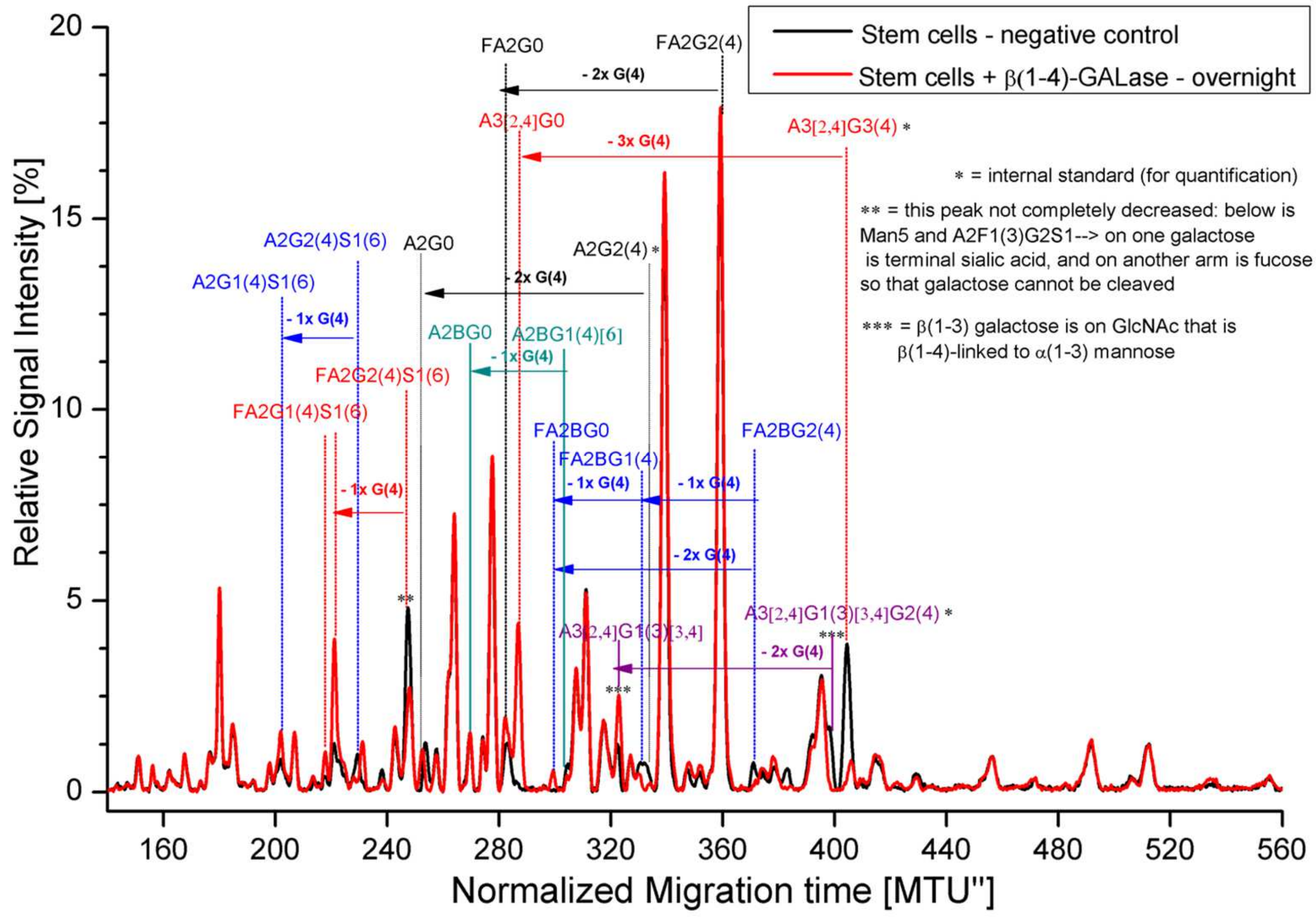

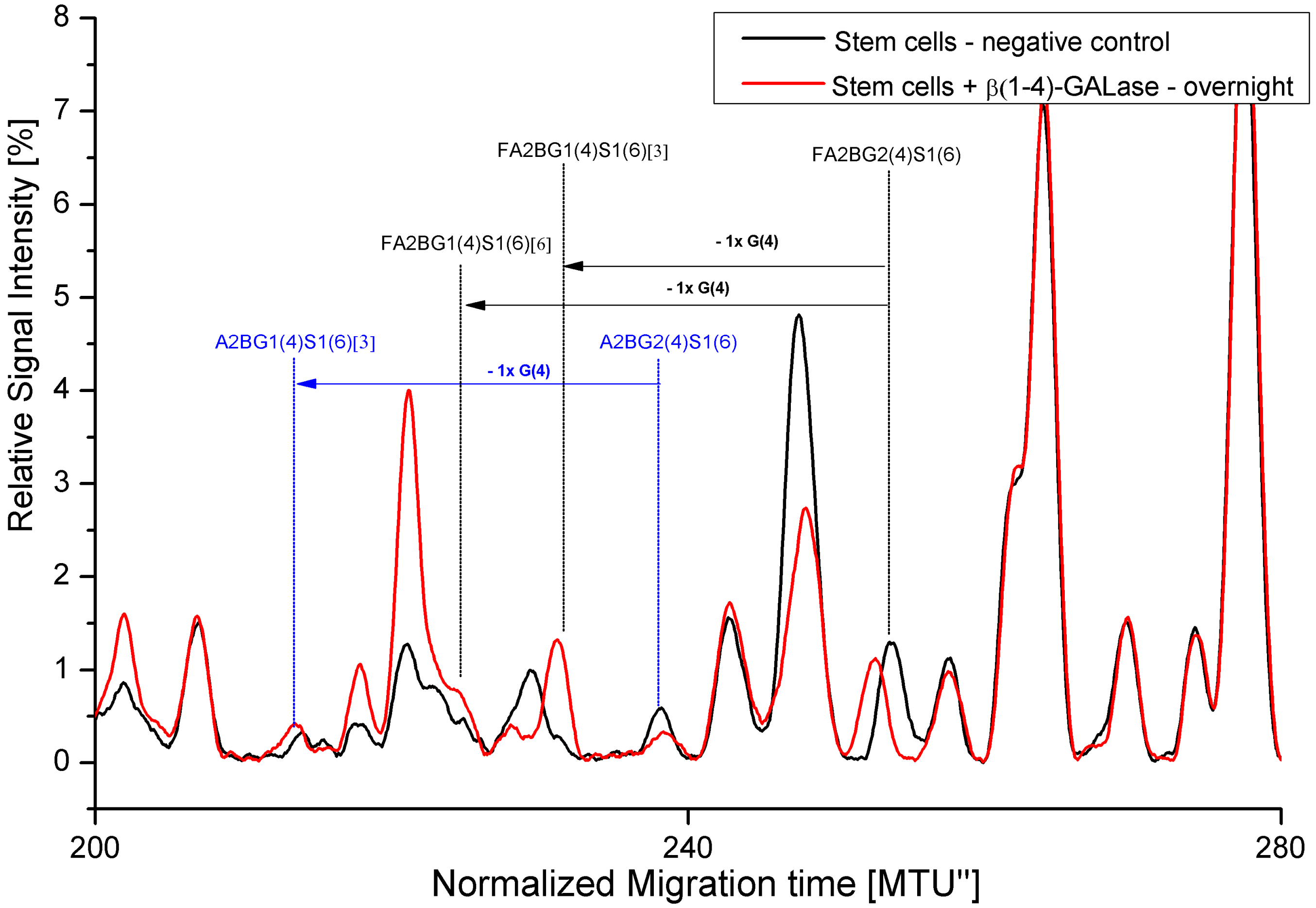
Stem cells - negative control
Stem cells + β(1-4)-GALase - overnight
FA2BG1(4)S1(6)[3]
FA2BG2(4)S1(6)
FA2BG1(4)S1(6)[6]
- 1x G(4)
- 1x G(4)
A2BG1(4)S1(6)[3]
A2BG2(4)S1(6)
- 1x G(4)
Relative Signal Intensity [%]
Normalized Migration time [MTU'']

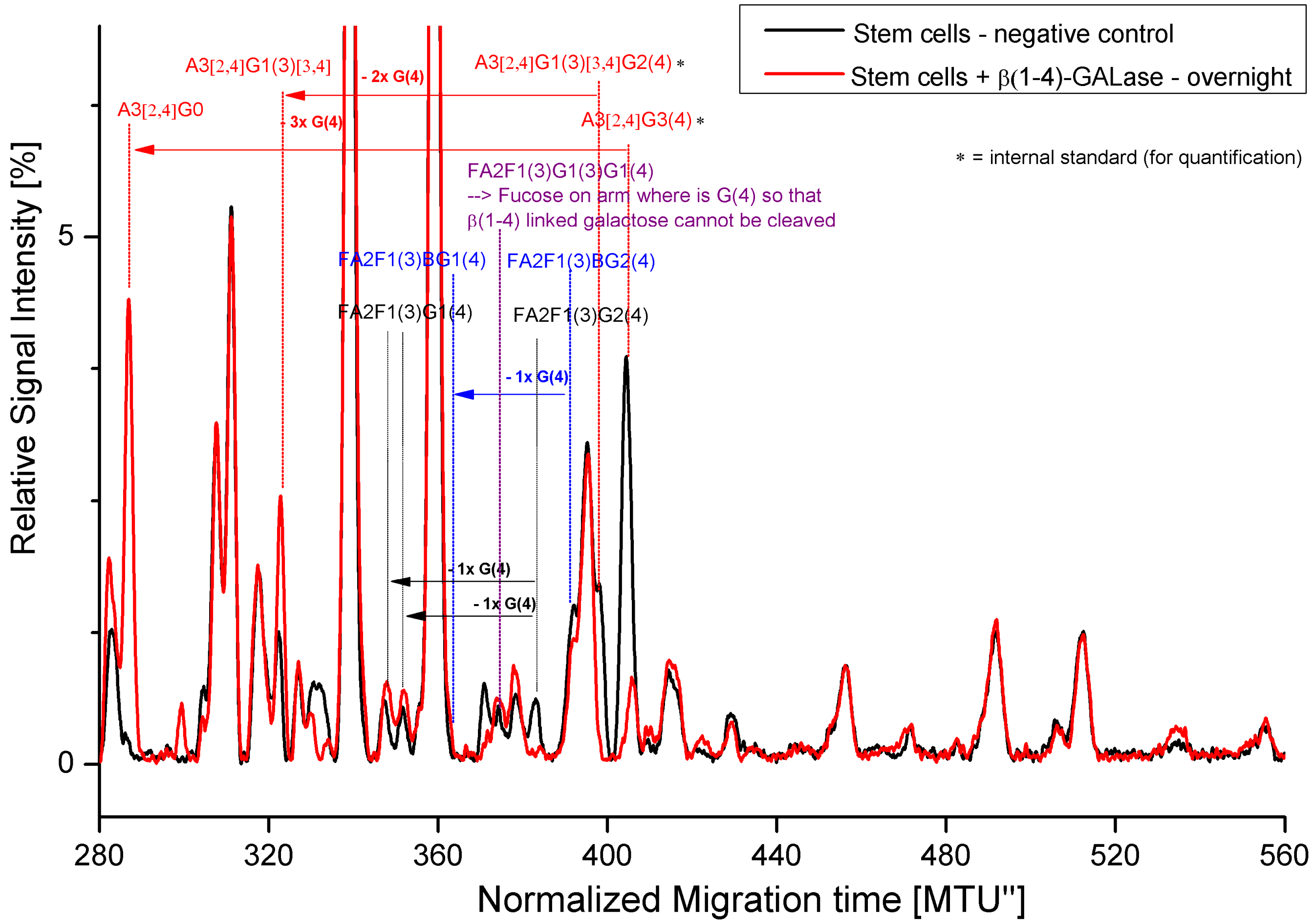
Stem cells - negative control
Stem cells + β(1-4)-GALase - overnight
A3[2,4]G1(3)[3,4]
- 2x G(4)
A3[2,4]G1(3)[3,4]G2(4) *
A3[2,4]G0
- 3x G(4)
A3[2,4]G3(4) *
* = internal standard (for quantification)
FA2F1(3)G1(3)G1(4)
--> Fucose on arm where is G(4) so that
β(1-4) linked galactose cannot be cleaved
FA2F1(3)BG1(4)
FA2F1(3)BG2(4)
FA2F1(3)G1(4)
FA2F1(3)G2(4)
- 1x G(4)
- 1x G(4)
- 1x G(4)
Relative Signal Intensity [%]
Normalized Migration time [MTU'']

Positive Control

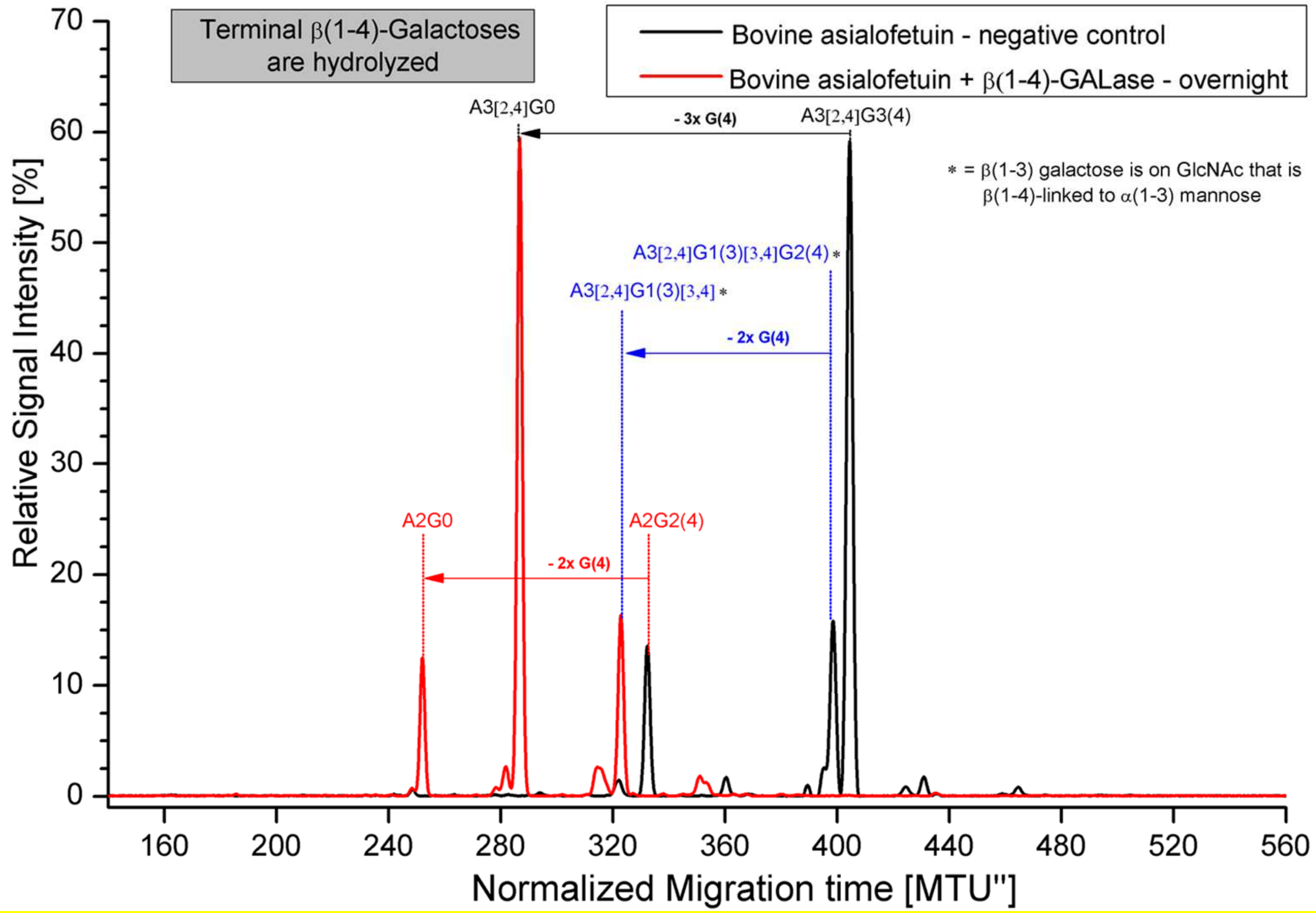

β(1-4,6) Galactosidase

ES03

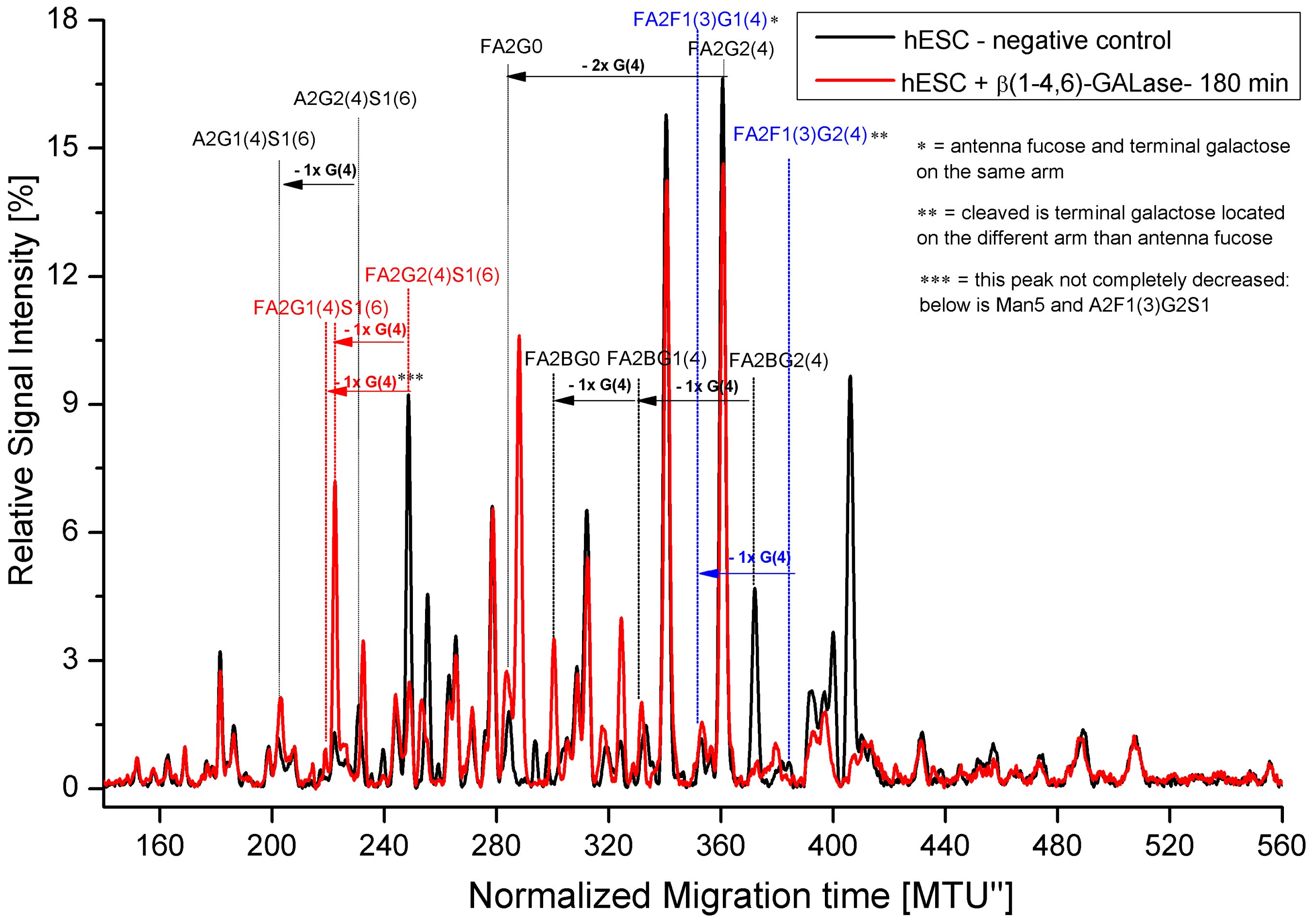

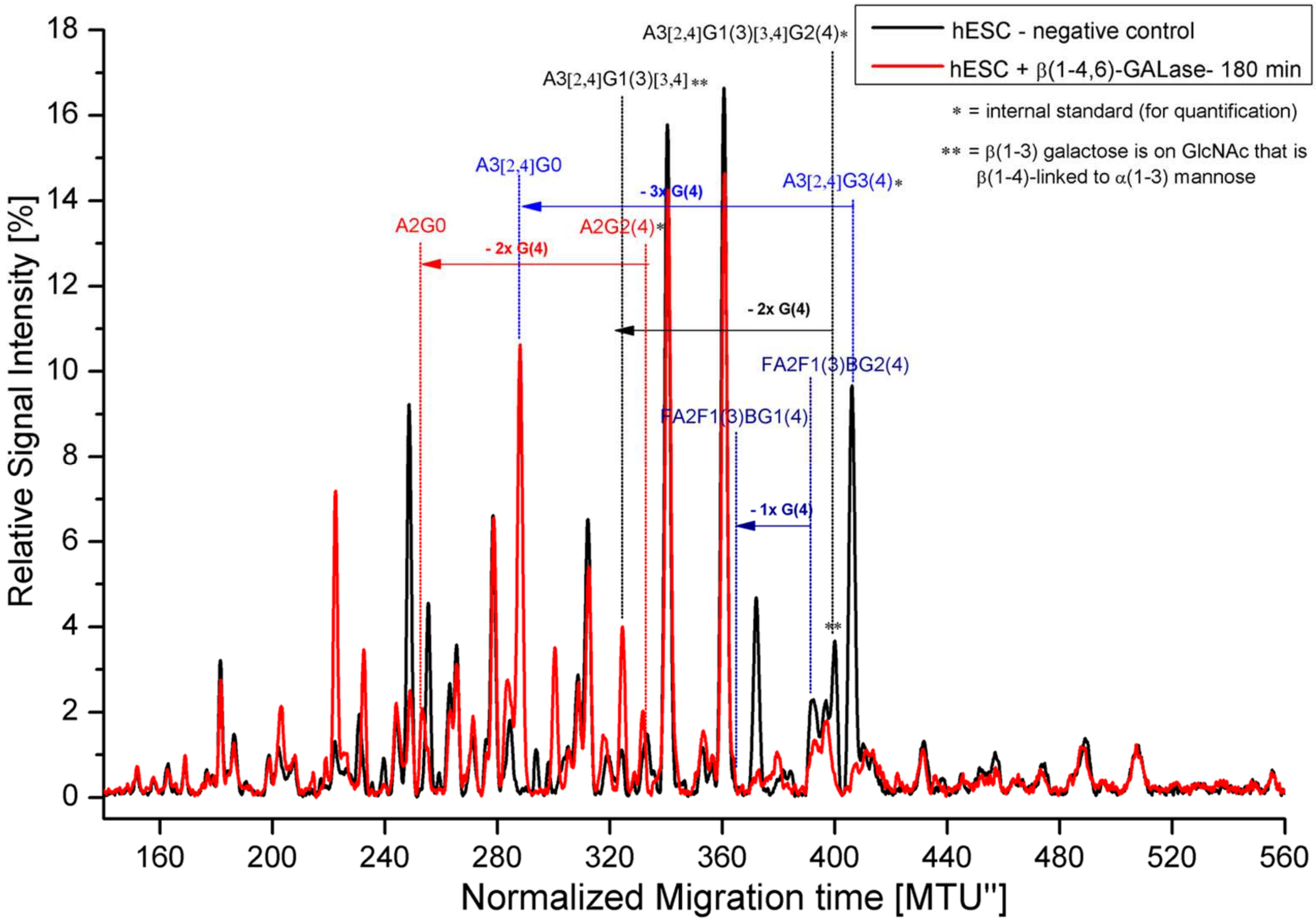
hESC - negative control
hESC + β(1-4,6)-GALase- 180 min
* = internal standard (for quantification)
** = β(1-3) galactose is on GlcNAc that is β(1-4)-linked to α(1-3) mannose
A3[2,4]G1(3)[3,4]G2(4)*
A3[2,4]G1(3)[3,4]**
A3[2,4]G0
- 3x G(4)
A3[2,4]G3(4)*
A2G0
- 2x G(4)
A2G2(4)*
- 2x G(4)
FA2F1(3)BG2(4)
FA2F1(3)BG1(4)
- 1x G(4)
**
Relative Signal Intensity [%]
Normalized Migration time [MTU'']

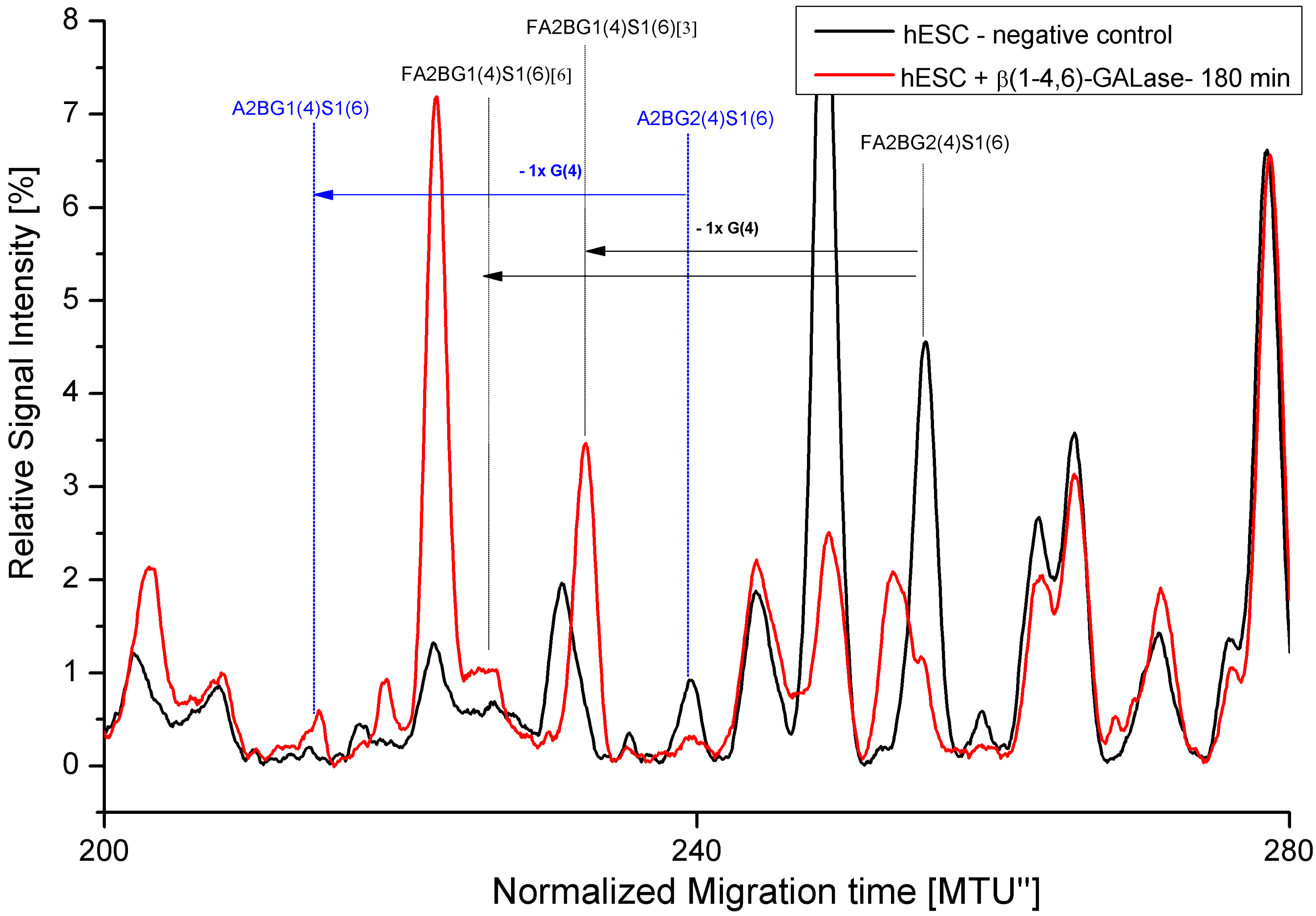
hESC - negative control
hESC + β(1-4,6)-GALase- 180 min
FA2BG1(4)S1(6)[3]
FA2BG1(4)S1(6)[6]
A2BG1(4)S1(6)
A2BG2(4)S1(6)
FA2BG2(4)S1(6)
- 1x G(4)
- 1x G(4)
Relative Signal Intensity [%]
Normalized Migration time [MTU'']

CBiPSC2

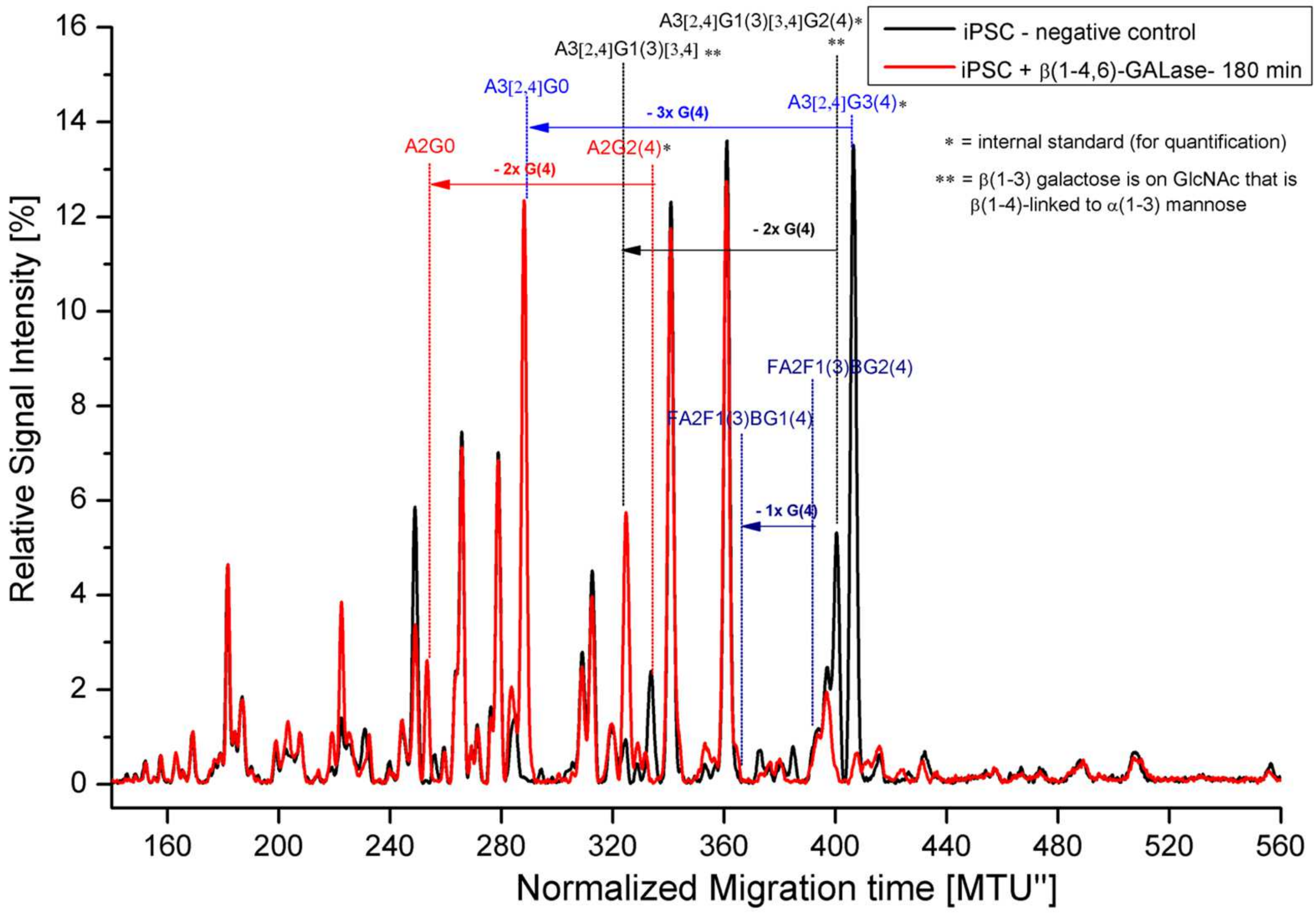

Relative Signal Intensity [%]
Normalized Migration time [MTU'']
iPSC - negative control
iPSC + β(1-4,6)-GALase- 180 min
A3[2,4]G1(3)[3,4]G2(4)*
**
A3[2,4]G1(3)[3,4] **
A3[2,4]G0
A3[2,4]G3(4)*
- 3x G(4)
A2G0
A2G2(4)*
- 2x G(4)
- 2x G(4)
* = internal standard (for quantification)
** = β(1-3) galactose is on GlcNAc that is β(1-4)-linked to α(1-3) mannose
FA2F1(3)BG2(4)
FA2F1(3)BG1(4)
- 1x G(4)

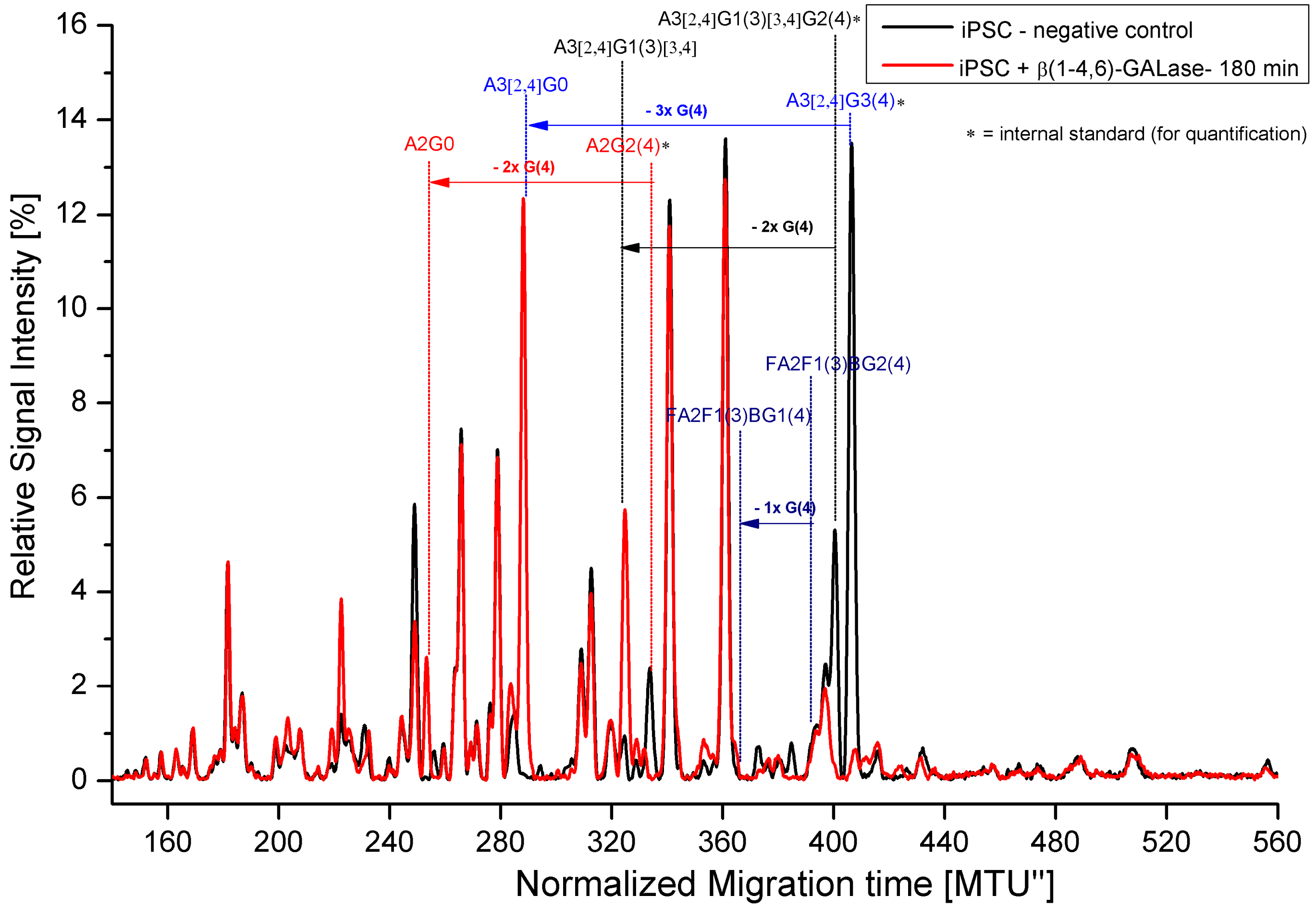

Relative Signal Intensity [%]
Normalized Migration time [MTU'']
iPSC - negative control
iPSC + β(1-4,6)-GALase- 180 min
A3[2,4]G1(3)[3,4]G2(4)*
A3[2,4]G1(3)[3,4]
A3[2,4]G0
A3[2,4]G3(4)*
- 3x G(4)
A2G0
A2G2(4)*
- 2x G(4)
- 2x G(4)
* = internal standard (for quantification)
FA2F1(3)BG2(4)
FA2F1(3)BG1(4)
- 1x G(4)

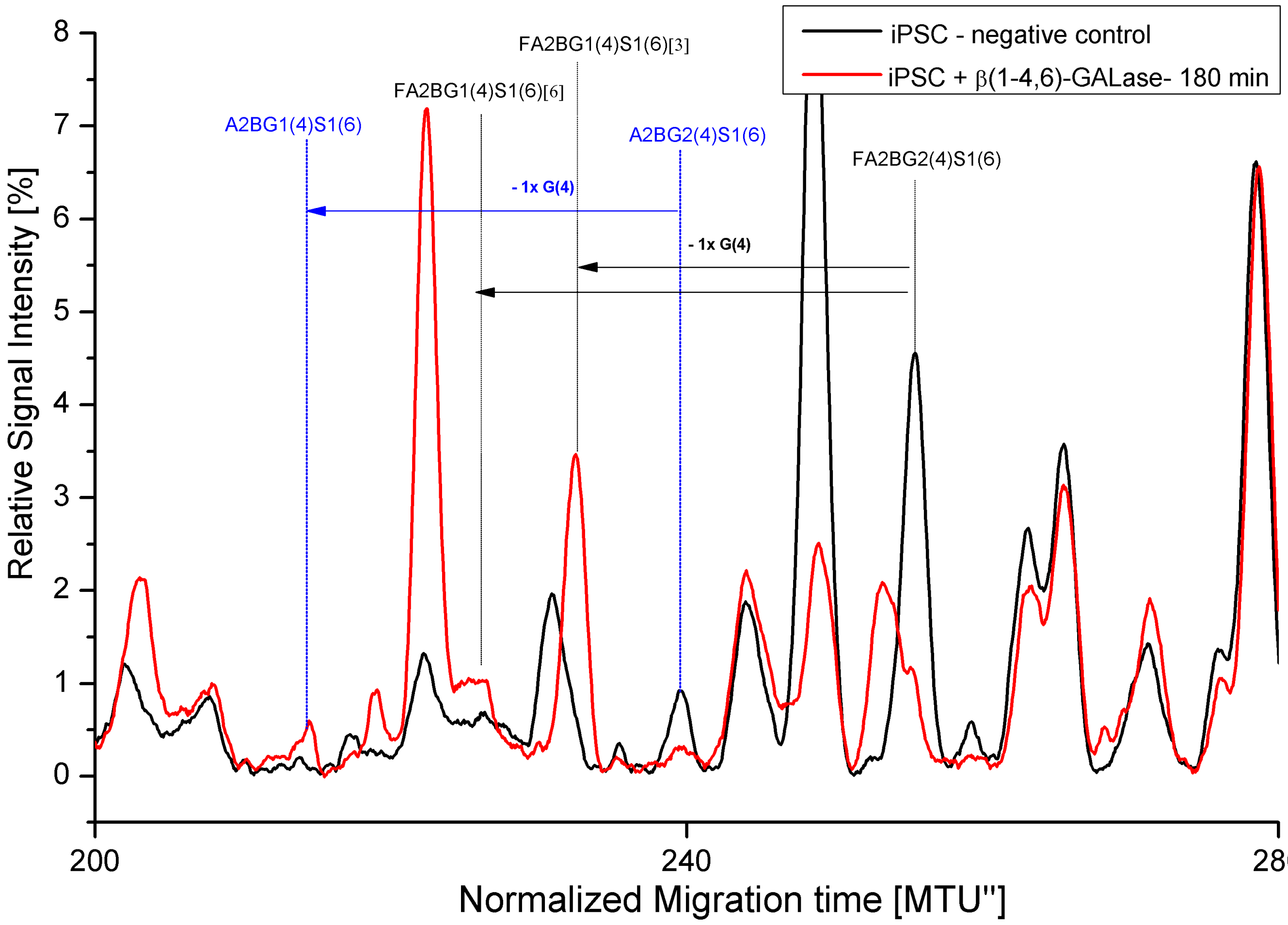

iPSC - negative control
iPSC + β(1-4,6)-GALase- 180 min
FA2BG1(4)S1(6)[3]
FA2BG1(4)S1(6)[6]
A2BG1(4)S1(6)
A2BG2(4)S1(6)
FA2BG2(4)S1(6)
- 1x G(4)
- 1x G(4)
Relative Signal Intensity [%]
Normalized Migration time [MTU"]

PMM2-iPSC-C3

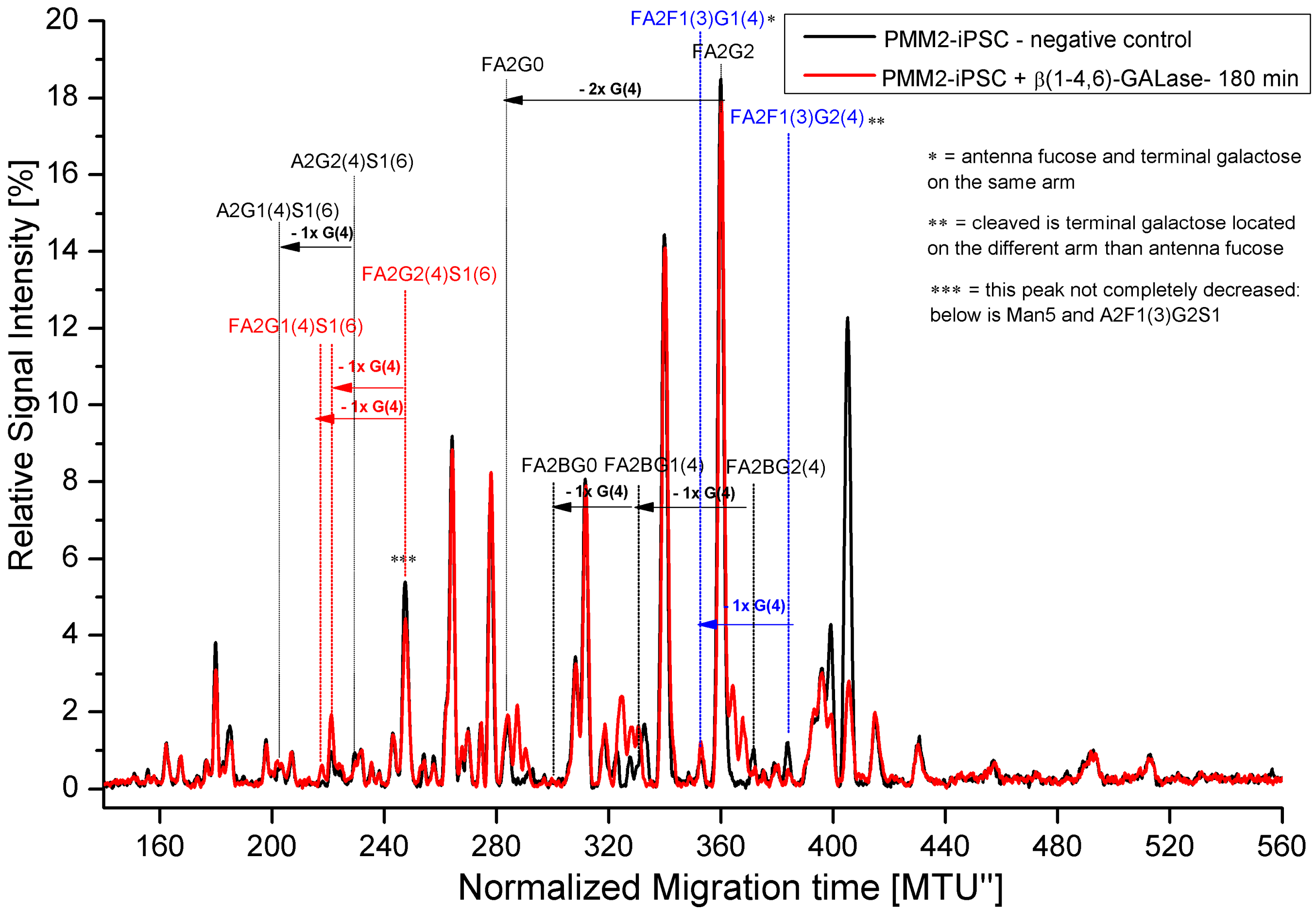

FA2F1(3)G1(4)*
PMM2-iPSC - negative control
PMM2-iPSC + β(1-4,6)-GALase- 180 min
FA2G2
FA2G0
- 2x G(4)
FA2F1(3)G2(4)**
A2G2(4)S1(6)
A2G1(4)S1(6)
- 1x G(4)
FA2G2(4)S1(6)
FA2G1(4)S1(6)
- 1x G(4)
- 1x G(4)
* = antenna fucose and terminal galactose on the same arm
** = cleaved is terminal galactose located on the different arm than antenna fucose
*** = this peak not completely decreased: below is Man5 and A2F1(3)G2S1
FA2BG0 FA2BG1(4) FA2BG2(4)
- 1x G(4)
- 1x G(4)
***
- 1x G(4)
Relative Signal Intensity [%]
Normalized Migration time [MTU"]

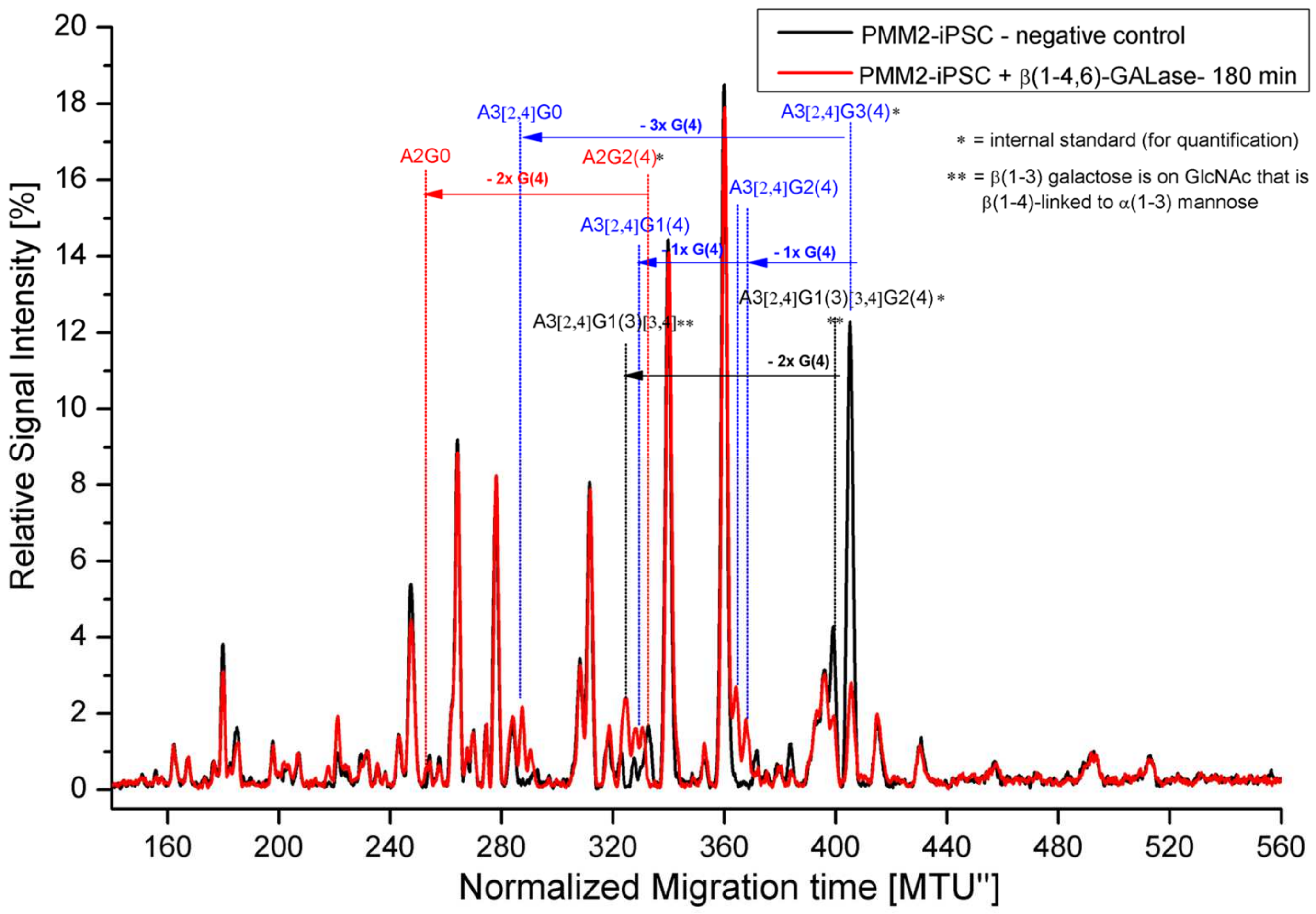

PMM2-iPSC - negative control
PMM2-iPSC + β(1-4,6)-GALase- 180 min
A3[2,4]G0
- 3x G(4)
A3[2,4]G3(4)*
* = internal standard (for quantification)
A2G0
- 2x G(4)
A2G2(4)*
** = β(1-3) galactose is on GlcNAc that is β(1-4)-linked to α(1-3) mannose
A3[2,4]G2(4)
A3[2,4]G1(4)
-1x G(4)
- 1x G(4)
A3[2,4]G1(3)[3,4]G2(4)*
A3[2,4]G1(3)[3,4]**
- 2x G(4)
Relative Signal Intensity [%]
Normalized Migration time [MTU'']

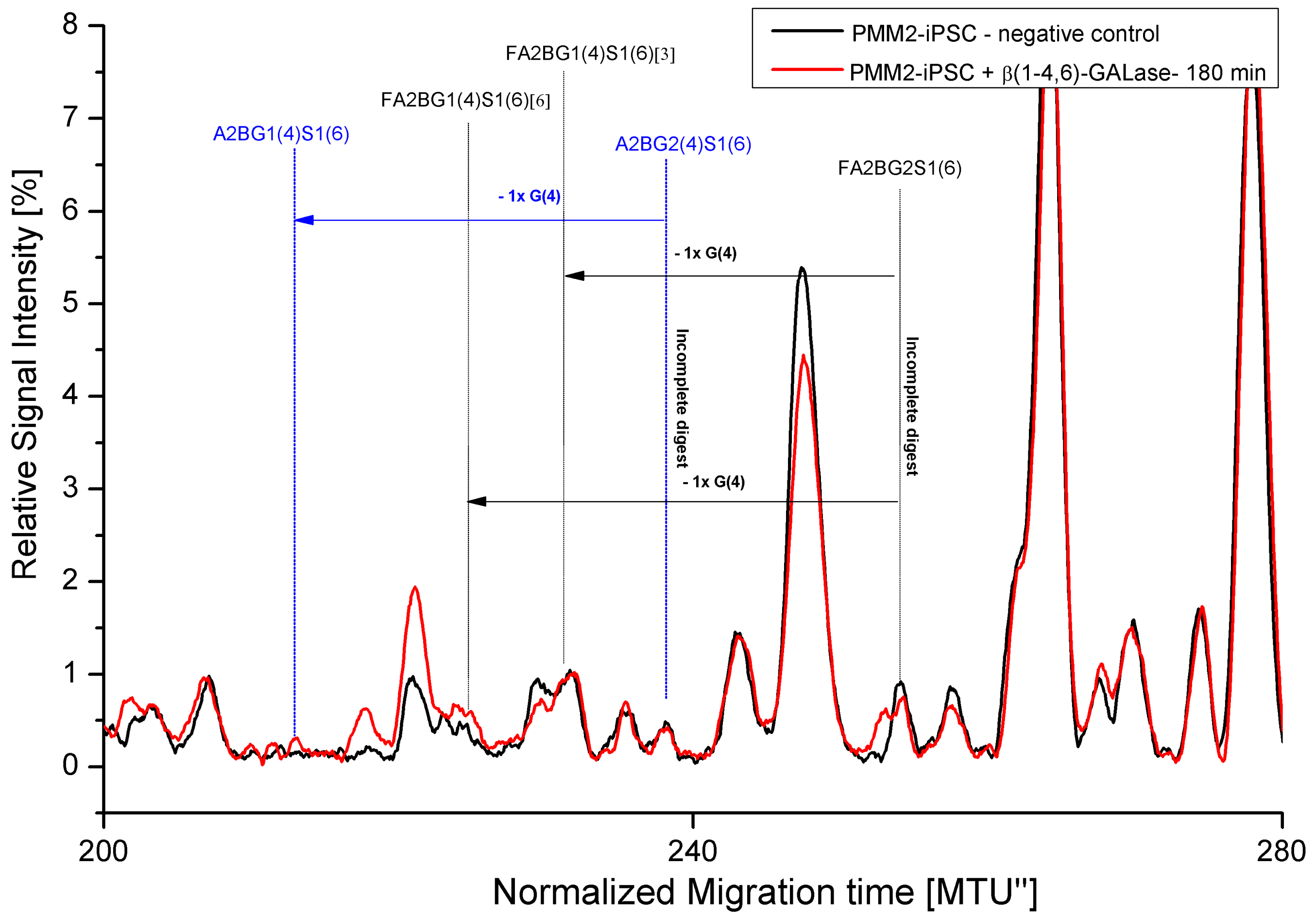

PMM2-iPSC - negative control
PMM2-iPSC + β(1-4,6)-GALase- 180 min
FA2BG1(4)S1(6)[3]
FA2BG1(4)S1(6)[6]
A2BG1(4)S1(6)
A2BG2(4)S1(6)
FA2BG2S1(6)
- 1x G(4)
- 1x G(4)
Incomplete digest
Incomplete digest
- 1x G(4)
Relative Signal Intensity [%]
Normalized Migration time [MTU'']

Positive Control

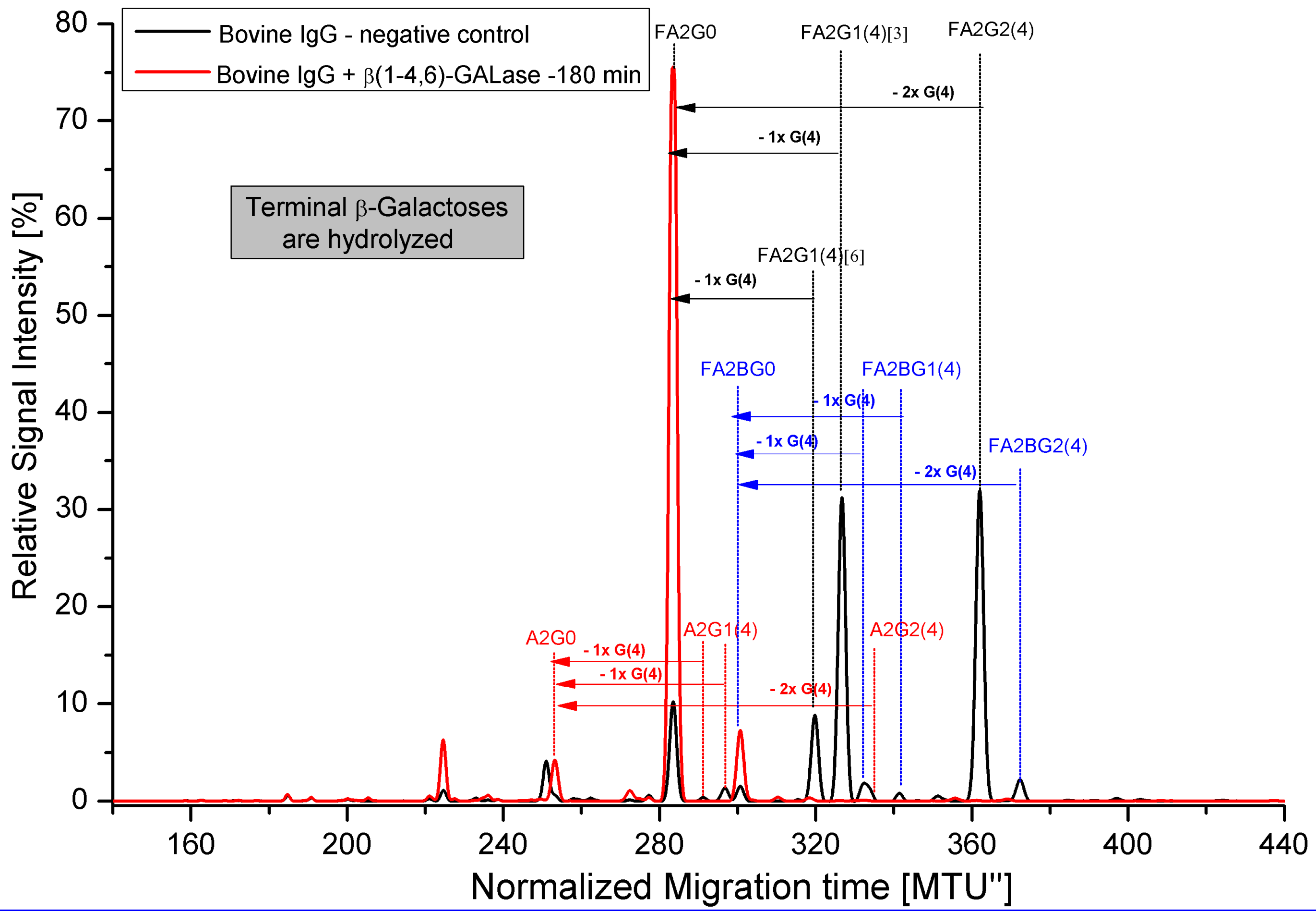

β(1-2,3,4,6) *N*-Acetylglucosaminidase

Positive Control

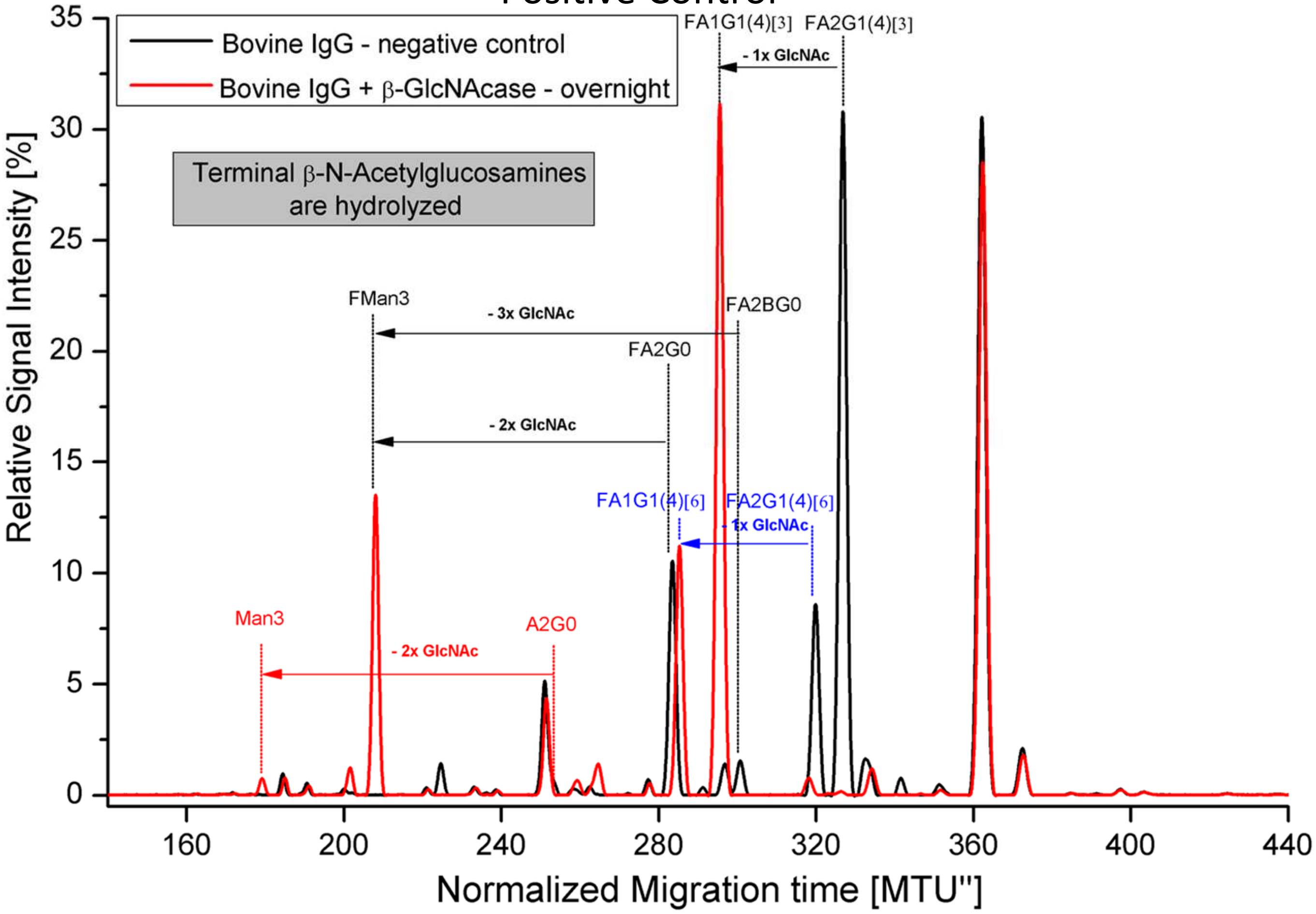

ES03

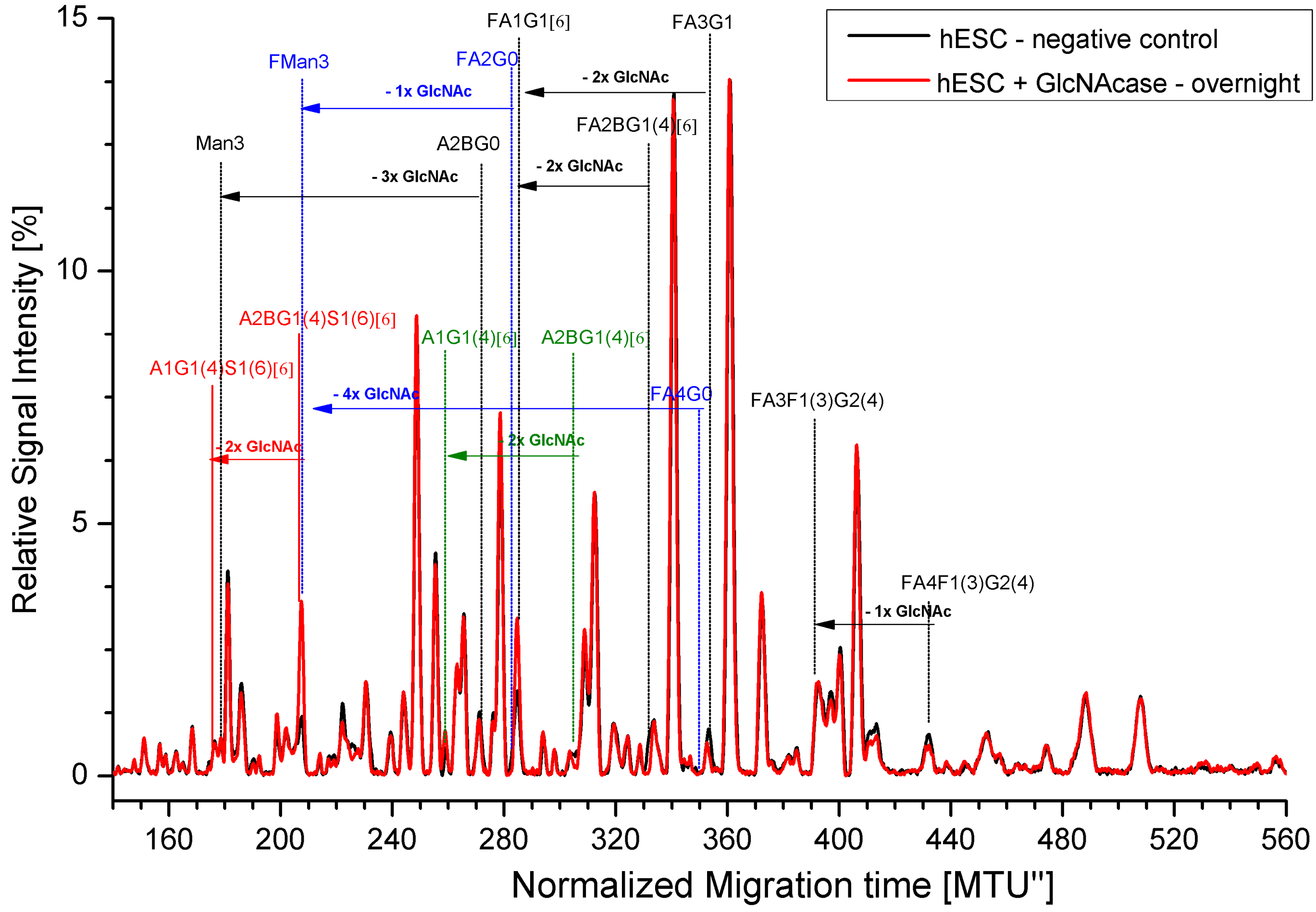

hESC - negative control
hESC + GlcNAcase - overnight
FA1G1[6]
FA3G1
FMan3
FA2G0
- 1x GlcNAc
- 2x GlcNAc
Man3
A2BG0
FA2BG1(4)[6]
- 3x GlcNAc
- 2x GlcNAc
A2BG1(4)S1(6)[6]
A1G1(4)[6]
A2BG1(4)[6]
A1G1(4)S1(6)[6]
- 4x GlcNAc
FA4G0
FA3F1(3)G2(4)
- 2x GlcNAc
- 2x GlcNAc
FA4F1(3)G2(4)
- 1x GlcNAc
Relative Signal Intensity [%]
Normalized Migration time [MTU'']

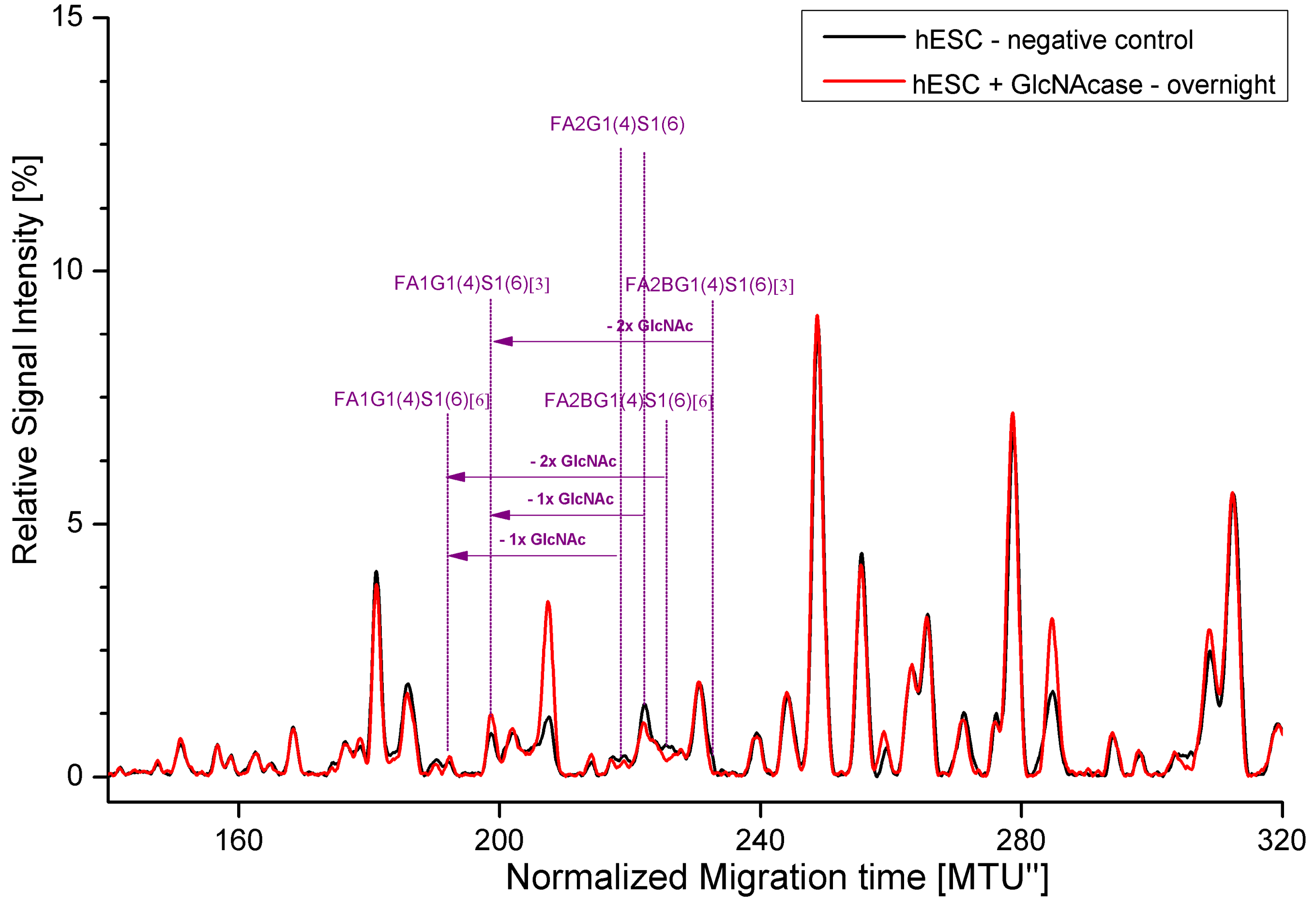

hESC - negative control
hESC + GlcNAcase - overnight
FA2G1(4)S1(6)
FA1G1(4)S1(6)[3]
FA2BG1(4)S1(6)[3]
- 2x GlcNAc
FA1G1(4)S1(6)[6]
FA2BG1(4)S1(6)[6]
- 2x GlcNAc
- 1x GlcNAc
- 1x GlcNAc
Relative Signal Intensity [%]
Normalized Migration time [MTU'']

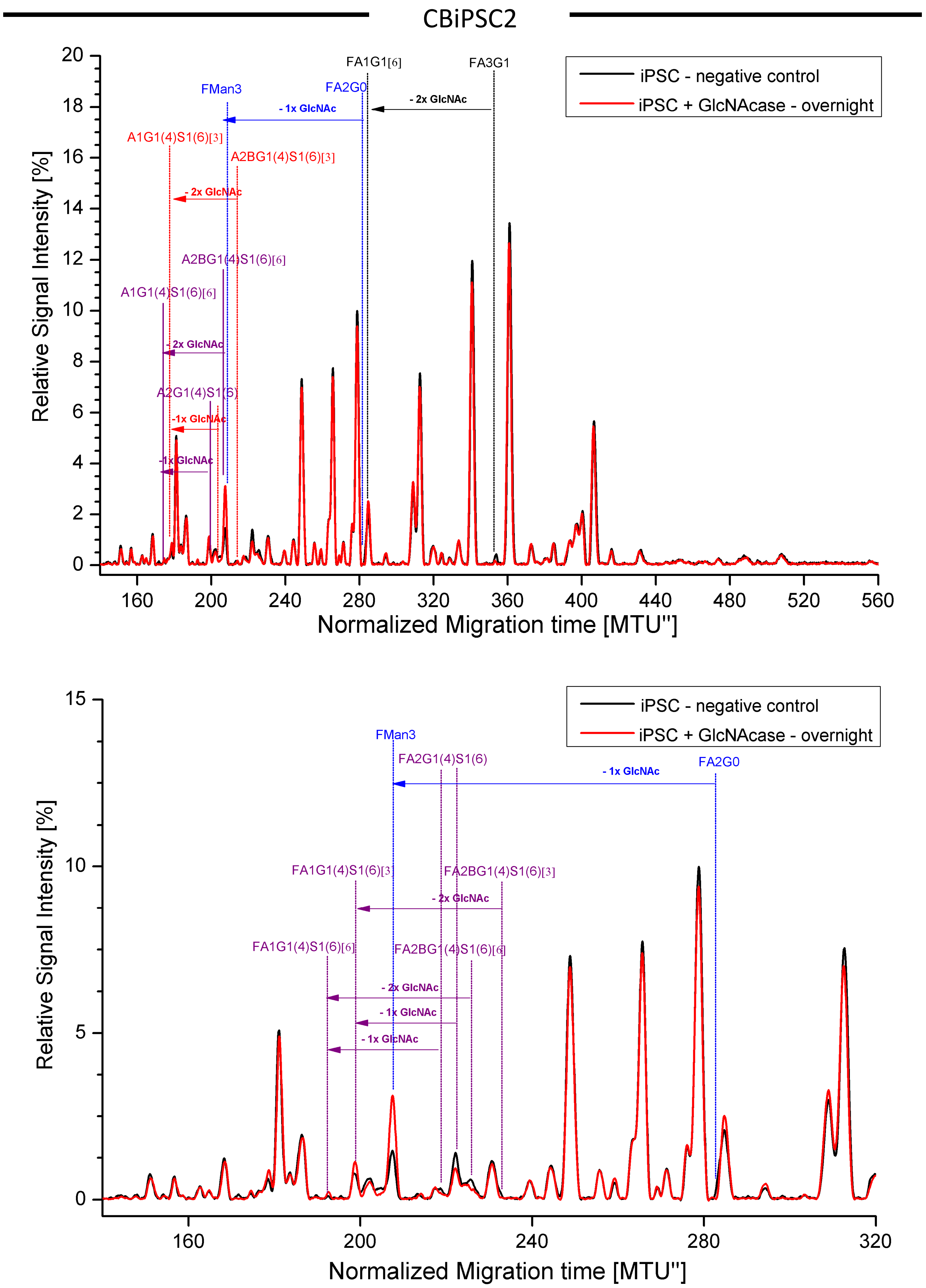
CBiPSC2
iPSC - negative control
iPSC + GlcNAcase - overnight
FA1G1[6]
FA3G1
FMan3
FA2G0
- 1x GlcNAc
- 2x GlcNAc
A1G1(4)S1(6)[3]
A2BG1(4)S1(6)[3]
A2BG1(4)S1(6)[6]
A1G1(4)S1(6)[6]
A2G1(4)S1(6)
-1x GlcNAc
Relative Signal Intensity [%]
Normalized Migration time [MTU'']
FA2G1(4)S1(6)
FA1G1(4)S1(6)[3]
FA2BG1(4)S1(6)[3]
FA1G1(4)S1(6)[6]
FA2BG1(4)S1(6)[6]

PMM2-iPSC-C3

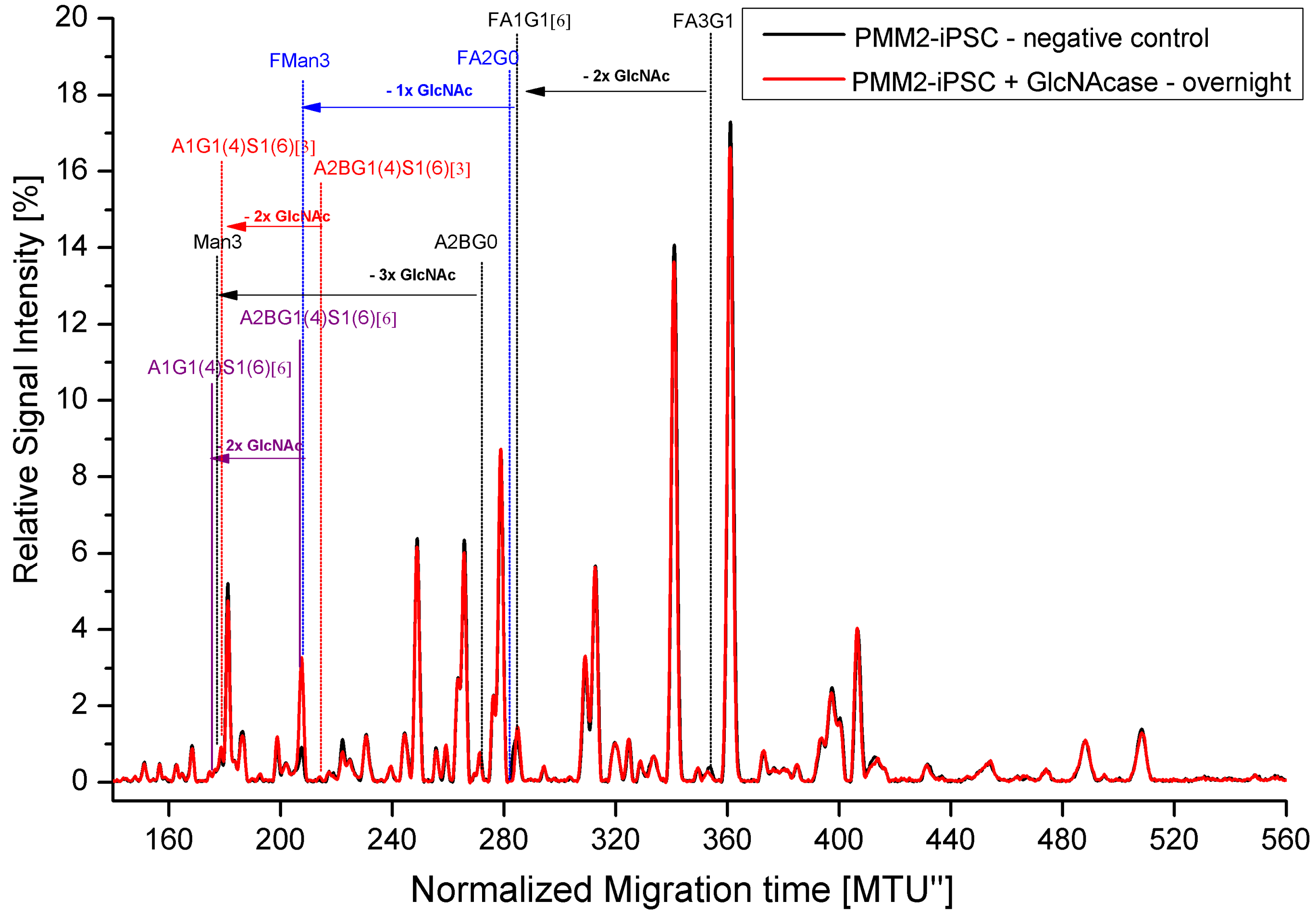

PMM2-iPSC - negative control
PMM2-iPSC + GlcNAcase - overnight
FA1G1[6]
FA3G1
FMan3
FA2G0
- 1x GlcNAc
- 2x GlcNAc
A1G1(4)S1(6)[3]
A2BG1(4)S1(6)[3]
- 2x GlcNAc
Man3
A2BG0
- 3x GlcNAc
A2BG1(4)S1(6)[6]
A1G1(4)S1(6)[6]
- 2x GlcNAc
Relative Signal Intensity [%]
Normalized Migration time [MTU'']

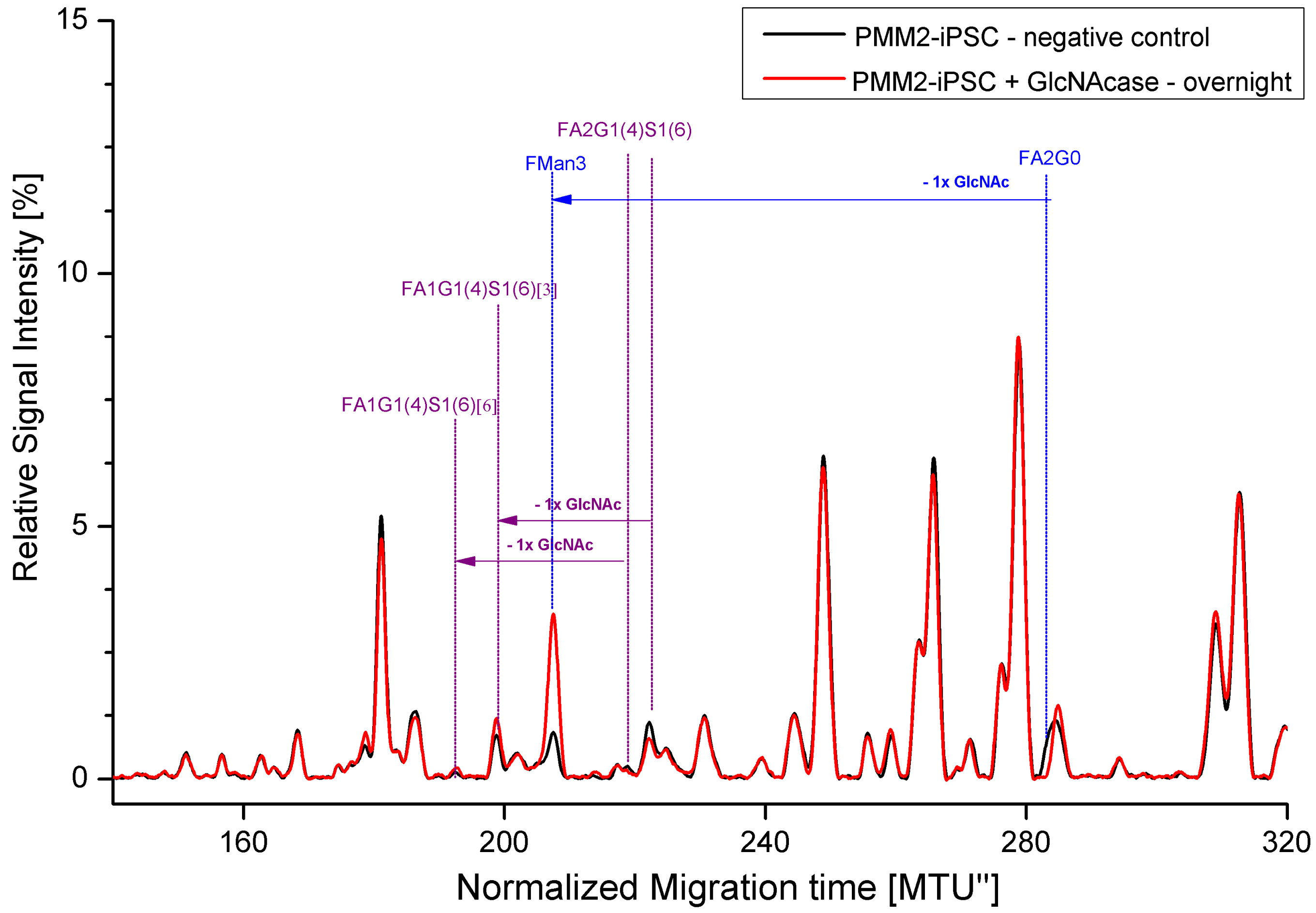

PMM2-iPSC - negative control
PMM2-iPSC + GlcNAcase - overnight
FA2G1(4)S1(6)
FMan3
FA2G0
- 1x GlcNAc
FA1G1(4)S1(6)[3]
FA1G1(4)S1(6)[6]
- 1x GlcNAc
- 1x GlcNAc
Relative Signal Intensity [%]
Normalized Migration time [MTU'']

α(1-2,3,6) Mannosidase

ES03

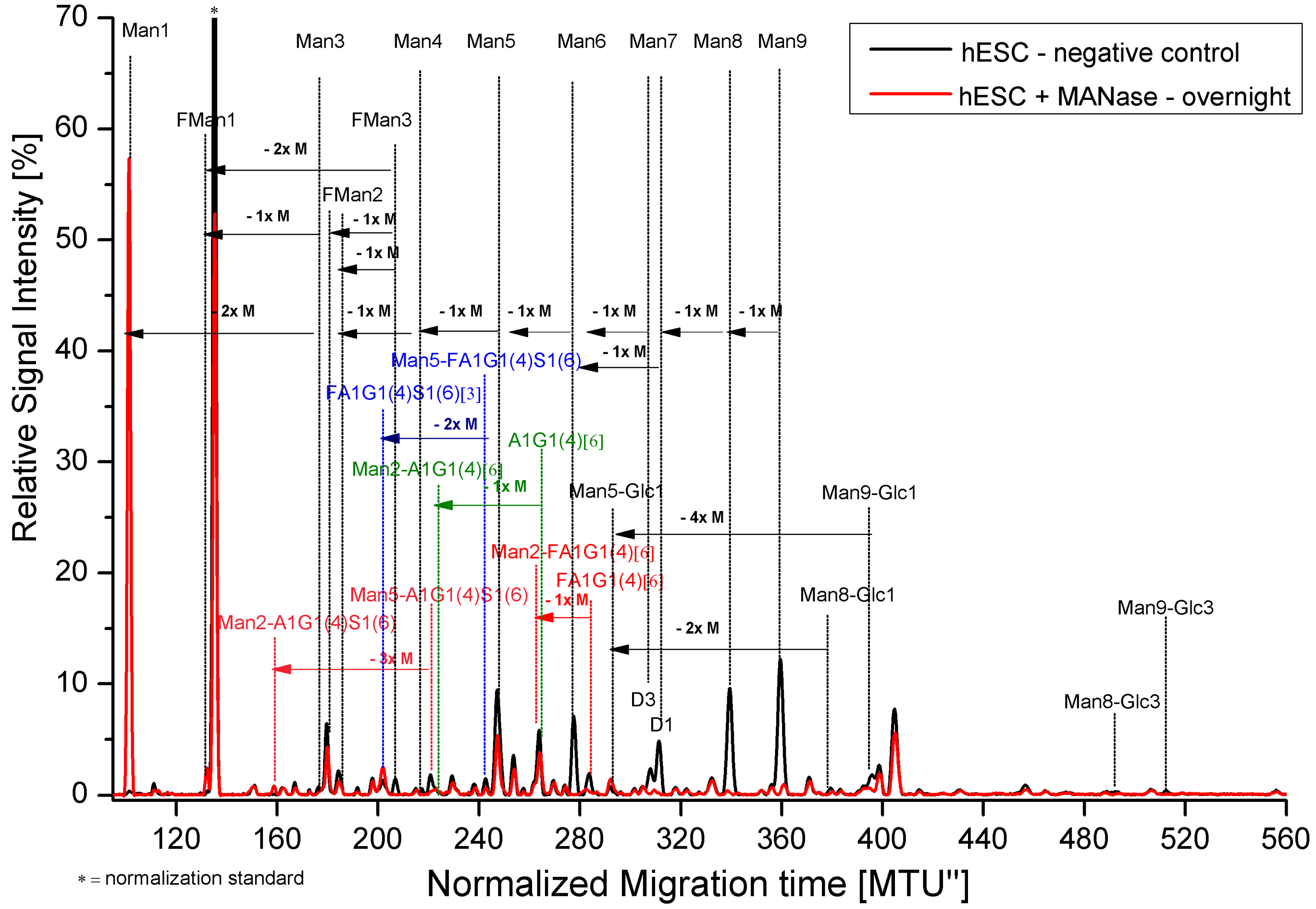

CBiPSC2

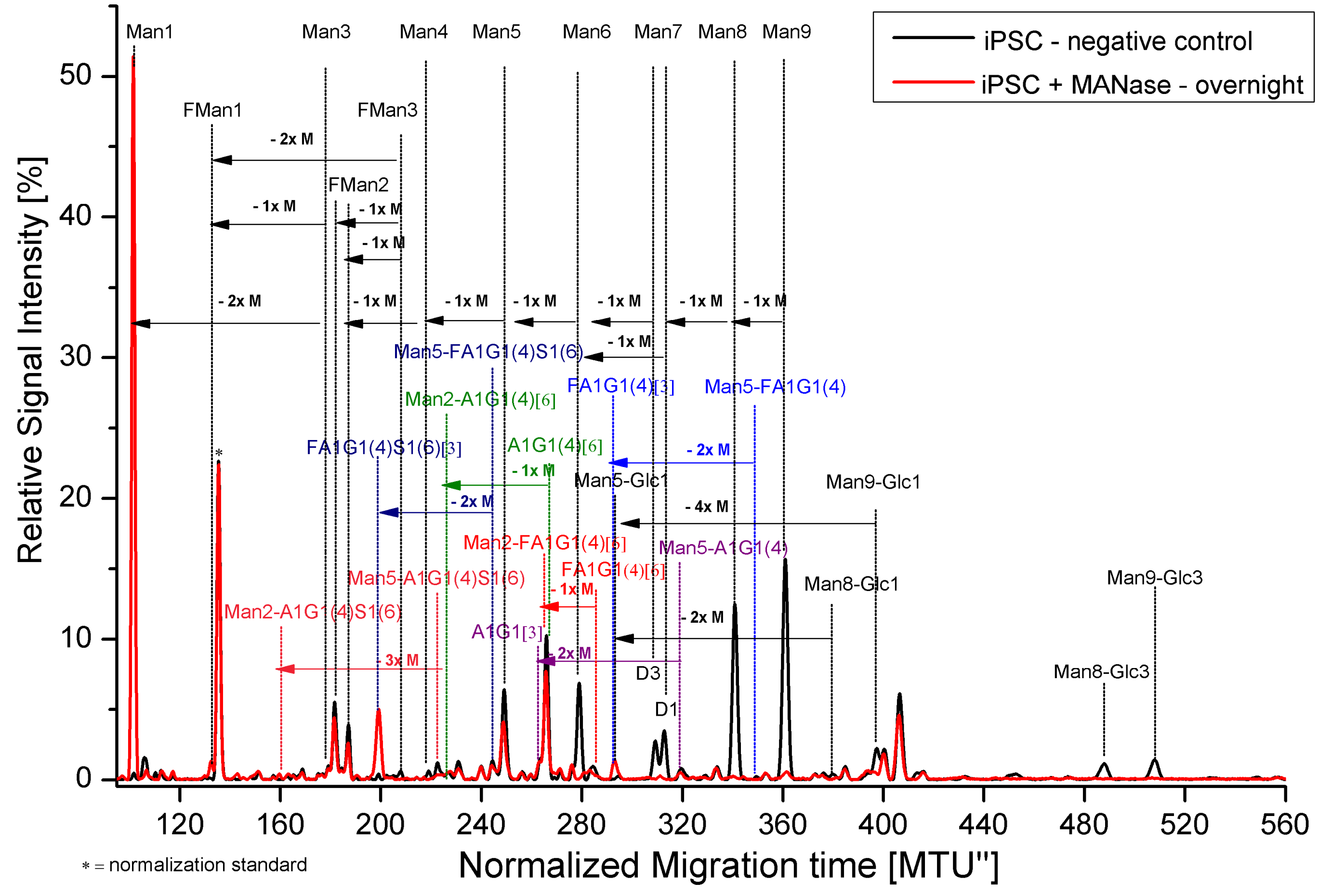

PMM2-iPSC-C3

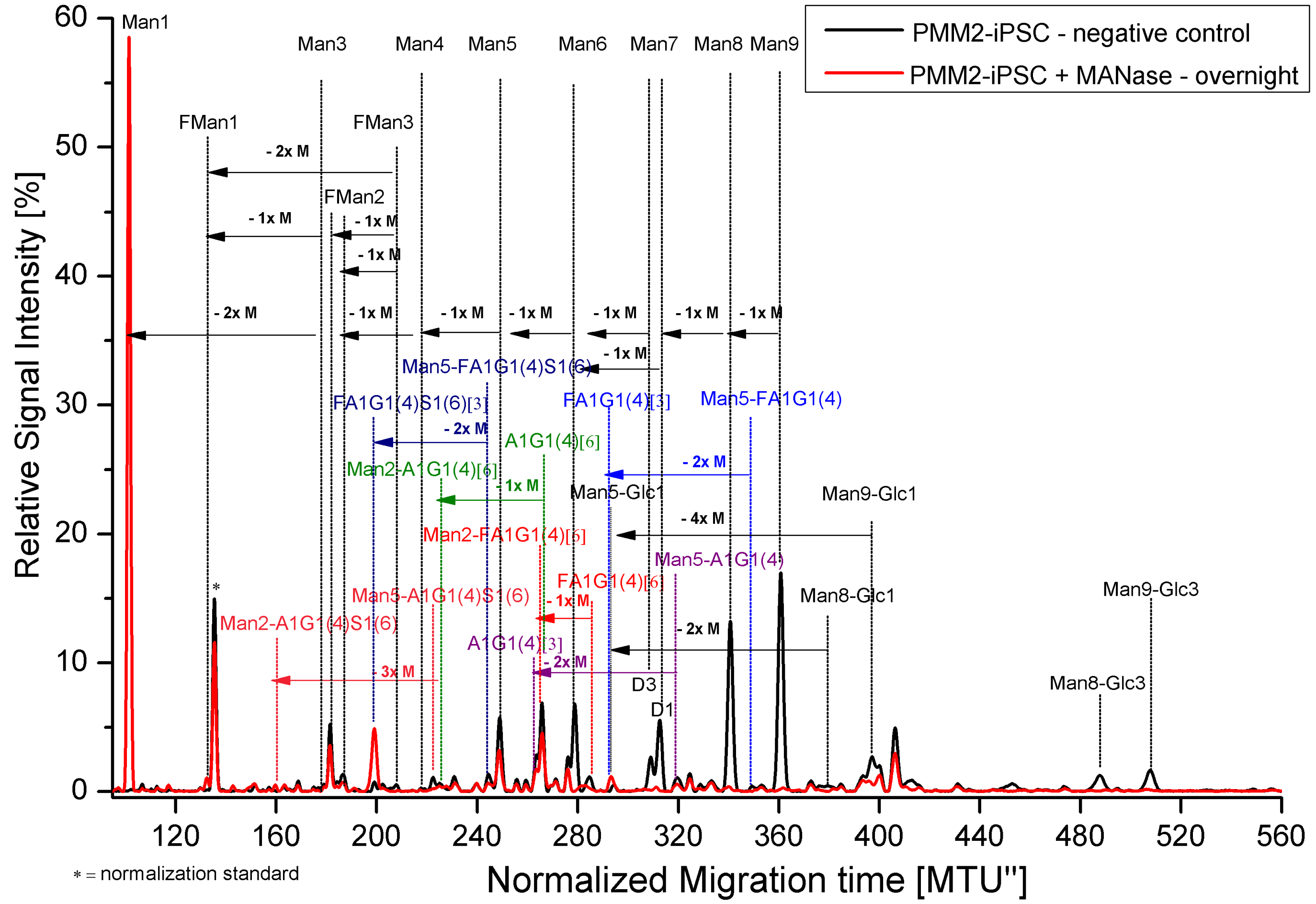

Positive Control

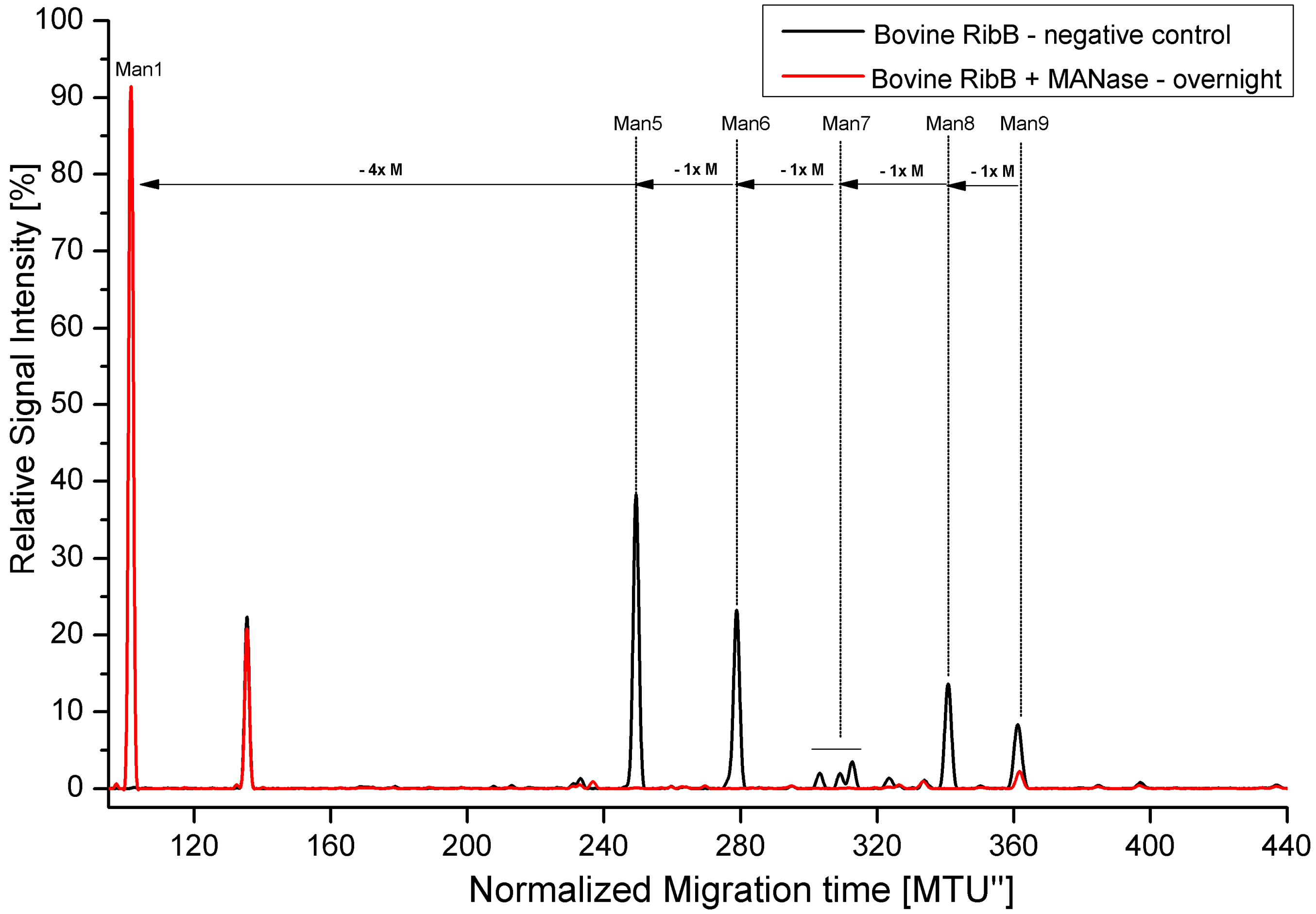

SUPPL. FIG. S2, related to Fig. 5. **Detailed analysis of stem cell N-glycosylation by exoglycosidase digests.** Exoglycosidase digests were performed on PMM2-iPSC-C3, CBiPSC2 and ES03 samples. For α(1-2) fucosidase, α(1-3,4) fucosidase, β(1-3) galactosidase and β(1-4) galactosidase digests, reaction was performed on mixture of equal amounts of PMM2-iPSC-C3, CBiPSC2 and ES03. Positive control was used for each enzyme in order to confirm its specific activity. Sialidase A releases α(2-3,6,8)-linked non-reducing terminal sialic acids. Sialidase S releases α(2-3)-linked non-reducing terminal sialic acids. α(1-3,4) fucosidase releases α(1-3,4)-linked non-reducing terminal fucose residues, whereas α(1-2) fucosidase removes α(1-2)-linked fucose and α(1-2,3,4,6) fucosidase releases α(1-6) core fucose more efficiently than α(1-3) and α(1-2)-linked non-reducing terminal fucose residues. β(1-4,6) galactosidase hydrolyses non-reducing terminal β(1-4) and β(1-6)-linked galactose, whereas β(1-4) galactosidase hydrolyses non-reducing terminal β(1-4) galactose, β(1-3) galactosidase β(1-3) releases non-reducing terminal β(1-3) galactose. β-N-acetylglucosaminidase digests β-N-acetylglucosamine (GlcNAc), but not a bisecting GlcNAc β(1-4)-linked to mannose. Jack bean α-mannosidase removes mannose linked α(1-2,3) > α(1-6). Structure abbreviations: all N-glycans have core sugar sequence consisting of two N-acetylglucosamines (GlcNAc) and three mannose; F at the start of abbreviation indicates a core α(1–6) fucose linked to the inner GlcNAc; Manx, number (x) of mannoses on core GlcNAcs; Ax, number (x) of antenna (GlcNAc) on trimannosyl core: A2, biantennary with both GlcNAcs β(1-2)-linked, A3[2,4], triantennary with a GlcNAc linked β(1-2) to both mannoses and the third GlcNAc linked β(1-4) to the α(1-3)-linked mannose; A4, GlcNAcs linked as A3[2,4] with additional GlcNAc β(1-6)-linked to α(1-6) mannose; B, bisecting GlcNAc linked β(1-4) to β(1-3) mannose; Gx, indicates number (x) of linked galactoses (Gal) on antennae, Gx(4) or Gx(3) indicates that the Gal is β(1-4) or β(1-3)-linked to antenna GlcNAc (if linkage is not specified, then the linkage is unknown); [3] and [6] at the end of the abbreviation indicate that the galactose is on the antenna of the α(1-3) or α(1-6) mannose; Fx(3) after Ax indicates number (x) of fucoses that are α(1-3)-linked to antennae GlcNAc;

Fx(2) after Gx indicates number (x) of fucoses that are α(1-2)-linked to antennae Gal; Sx, number (x) of sialic acids (N-acetylneuraminic acid) linked to galactose; Sx(3) and Sx(6) indicate whether the sialic acid is in an α(2-3) or α(2-6)-linkage; Glcx, number of glucoses at the non-reducing end.

Figure S3

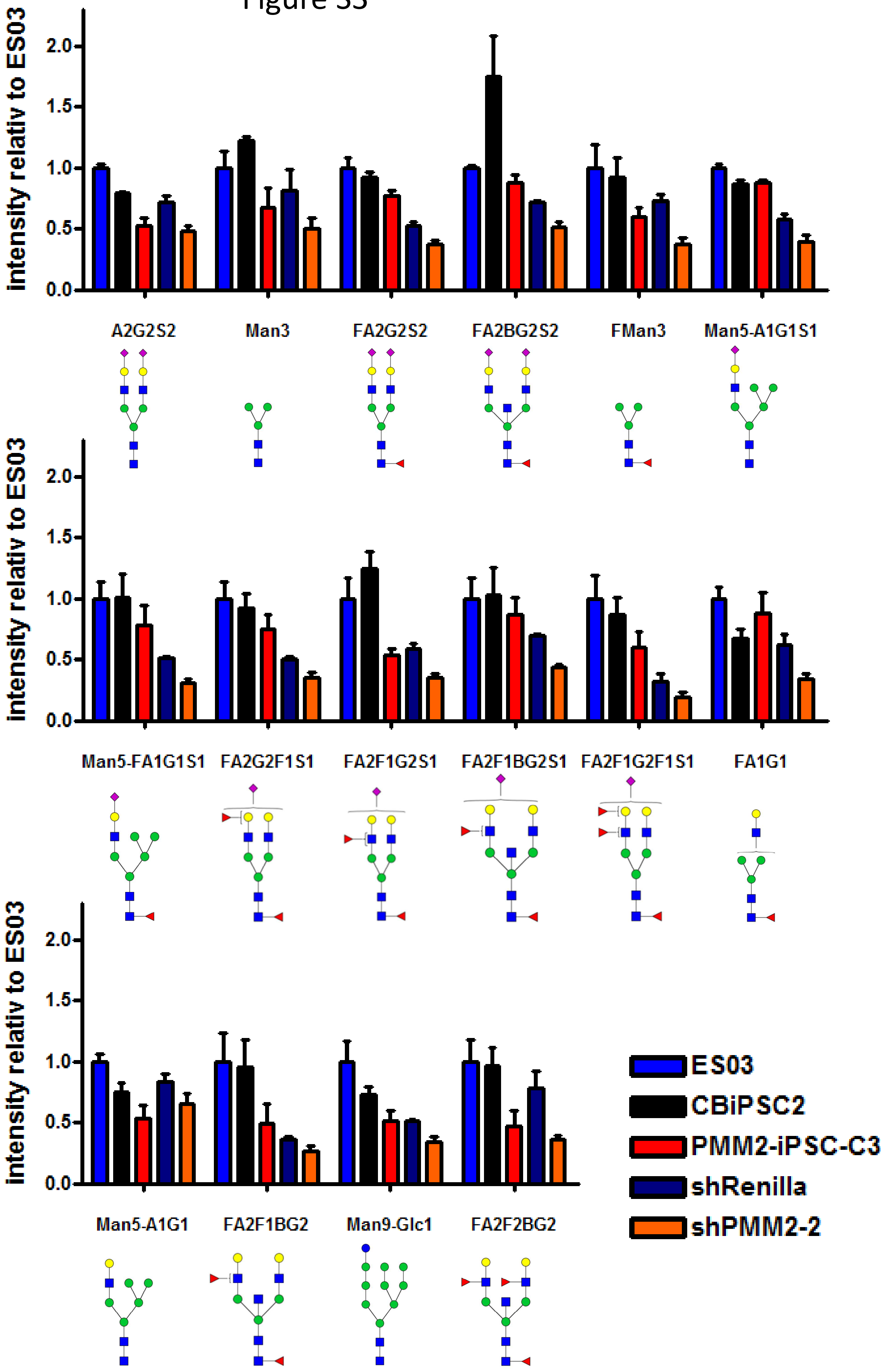
intensity relativ to ES03
2.0
1.5
1.0
0.5
0.0
A2G2S2
Man3
FA2G2S2
FA2BG2S2
FMan3
Man5-A1G1S1
Man5-FA1G1S1
FA2G2F1S1
FA2F1G2S1
FA2F1BG2S1
FA2F1G2F1S1
FA1G1
Man5-A1G1
FA2F1BG2
Man9-Glc1
FA2F2BG2
ES03
CBiPSC2
PMM2-iPSC-C3
shRenilla
shPMM2-2

SUPPL. FIG. S3, related to Fig. 5. **Quantification of hybrid- and complex-type N-glycans by quantitative xCGE-LIF.** Quantitative comparison of hybrid and complex-type N-glycan levels of ES03, CBiPSC2, PMM2-iPSC-C3, PMM2-iPSC-C3 transduced with shRenilla and PMM2-iPSC-C3 transduced with shPMM2-2. 200 µg protein of each sample were loaded and individual spectra were quantitatively normalized to a defined spiked-in asialofetuin N-glycan. Only peaks with a signal to noise ratio $S/N \geq 9$ were selected for quantification. Bar diagram shows mean + S.E.M, n = 3 each. The mean intensity of ES03 was set to 1 and intensities for other cell lines are relative to ES03. N-glycan structures were drawn with GlycoWorkbench v.1.0.3353 (1) by the guidelines of the Consortium for Functional Glycomics (2). Green circle: mannose; yellow circle: galactose; blue circle: glucose; blue quadratic rectangle: N-acetylglucosamine; violet diamonds: sialic acid; red triangle: fucose.

SUPPL. TABLE S1 related to Fig. 6: **shRNAs.**

Name	Sequence
shRNA Renilla	Fellmann *et al.*, 2013 (3)
shRNA PMM2_1	TGCTGTTGACAGTGAGCGACGGCAGAAAATTACCAAAGAATAGTGAAGCCACAGA TGTATTCTTTGGTAATTTTCTGCCGCTGCCTACTGCCTCGGA
shRNA PMM2_2	TGCTGTTGACAGTGAGCGCCGGAAAGAGTTTGCTGGAAAATAGTGAAGCCACAGA TGTATTTTCCAGCAAACTCTTTCCGTTGCCTACTGCCTCGGA
shRNA PMM2_3	TGCTGTTGACAGTGAGCGCTGAGTTCTACGAACTCGATAATAGTGAAGCCACAGA TGTATTATCGAGTTCGTAGAACTCAATGCCTACTGCCTCGGA

SUPPL. TABLE S2 related to Fig. 2 and Fig. 6: **Primary antibodies used for immunofluorescence (IF) and Western blots (WB)**.

Target	Host species (isotype)	Supplier	Supplier #	Clone	Dilution
OCT3/4 / Octamer-binding protein 3/4 / POU domain, class 5, transcription factor 1	mouse (IgG_{2b})	Santa Cruz Biotechnology	sc-5279	C-10	1:50 (IF)
SSEA-4 Stage-specific embryonic antigen-4	mouse ($IgG_{3\kappa}$)	BioLegend	330401	MC-813-70	1:100 (IF)
Alpha 1 antitrypsin	rabbit IgG	Abcam	ab166610	EPR9090	1:1000 (WB)

SUPPL. TABLE S3 related to Fig. 2, Fig. 4 and Fig. 6: **Secondary antibodies used for immunofluorescence (IF), Western blots (WB) and lectin blots (LB)**.

Target species (isotype)	Host species	Supplier	Supplier #	Reporter	Dilution (Application)
Anti-mouse (IgG_{2b})	goat	Molecular Probes	A 21141	Alexa Fluor® 488	1:250 (IF)
Anti-mouse IgG (whole molecule) $F(ab')_2$ fragment	sheep	Sigma Aldrich	C2181	Cy3	1:250 (IF)
Anti-digoxigenin-POD, Fab fragments	sheep	Roche	11633716001	Peroxidase	1:8000 (LB)
Anti-digoxigenin-AP, Fab fragments	sheep	Roche	11093274910	Alkaline phosphatase	1:1000 (LB)

SUPPL. TABLE S4 related to Fig. 2 and Fig. 6: **Primer used for qPCR.**

**Target/ full gene name**	**Primer name**	**Sequence (5' to 3')**
OCT3/4 / POU5F1 / POU class 5 homeobox 1	OCT 3/4_FW	AGAAGGAGAAGCTGGAGCAA
	OCT3/4_RV	CTTCCCAAATAGAACCCCCA
NANOG / Nanog homeobox	NANOG_FW	TACCTCAGCCTCCAGCAGAT
	NANOG_RV	TGCGTCACACCATTGCTATT
PAX6	PAX6_FW	CCTACGCAAGATGGCTGCC
	PAX6_RV	TTGCTCTTGGGTAAAGGATGTTC
T, brachyury homolog (mouse) (T)	T_FW	AATTGGTCCAGCCTTGGAAT
	T_RV	CGTTGCTCACAGACCACAG
SOX17 / SRY (sex determining region Y)-box 17	SOX17_FW	CAG AAT CCA GAC CTG CAC AA
	SOX17_RV	TACTTGTAGTTGGGGTGGTC
NKX2-5 / NK2 homeobox 5	NKX2-5_FW	AGAAGACAGAGGCGGACAAC
	NKX2-5_RV	CGCCGCTCCAGTTCATAG
MYH6 / myosin, heavy chain 6, cardiac muscle, alpha	MYH6_FW	ATTGCTGAAACCGAGAATGG
	MYH6_RV	CGCTCCTTGAGGTTGAAAAG
α1AT / SERPINA1 serpin peptidase inhibitor, clade A (alpha-1 antiproteinase, antitrypsin), member 1	a1AT_FW	ACCAGTCCAACAGCACCAAT
	a1AT_RV	AGGATTTCATCGTGAGTGTC
PMM2/ Phosphomannomutase 2	PMM2_FW	GGTAGCATACAAAGATGGGA
	PMM2_RV	TCGCAATGTAGCTCAGACAG
SDHB /succinate dehydrogenase complex, subunit B, iron sulfur protein	SDHB_FW	CTTCCGAAGATCATGCAGAG
	SDHB_RV	CATGTGTGGAAGAGGGTAGA
ARPC1A / actin related protein 2/3 complex, subunit 1A	ARPC1A_FW	GTGGAGCACGACTCATTTCT
	ARPC1A_RV	TGATCCTGCTGCCAGCAAAA
GAPDH / glyceraldehyde-3-phosphate dehydrogenase	GAPDH_FW	TCAAGGCTGAGAACGGGAAG
	GAPDH_RV	ATGGTGGTGAAGACGCCAGT

SUPPL. TABLE S5 related to Fig. 4 and Fig. 6: **Lectins used for lectin flow cytometry (LFC) and lectin blots (LB).**

**Lectin / label / abbreviation**	**Sugar specificity**	**Manufacturer**	**Code**	**Conc. / Dilution**
Galanthus nivalis agglutinin / digoxigenin-labeled (GNA-DIG)	α-1,3- and α-1,6-linked terminal mannose in oligo-mannose-type N-glycans (4)	Roche	11210238001	1:1000 for LB
Galanthus nivalis agglutinin / fluorescein isothiocyanate conjugate / (GNA-FITC)	α-1,3- and α-1,6-linked terminal mannose in oligo-mannose-type N-glycans (4)	Vector Laboratories	FL-1241	2 mg/ml (stock solution) / 1:200 for LFC
Galantus nivalus agglutinin / *Texas Red*® labelled / (GNA-Texas Red)	α-1,3- and α-1,6-linked terminal mannose in oligo-mannose-type N-glycans (4)	EY Laboratories	T-7401-1	1 mg/ml (stock solution) / 1:50 for LFC
Concanavalin A / tetramethylrhodamine isothiocyanate conjugate (Con A-TRITC)	High-mannose-type N-glycans and with less affinity complex-type biantennary glycans (5)	HiMedia Laboratories	C860	5 mg/ml (stock solution) / 1:250 for LFC
Sambucus nigra lectin (SNA) / tetramethylrhodamine isothiocyanate conjugate / (SNA-TRITC)	Neu5Acα(2,6)Gal/GalNAc (6)	EY Laboratories	F-7801-2	1 mg/ml (stock solution) / 1:100 for LFC
Maackia amurensis lectin (MAA) / fluorescein isothiocyanate	Sialic acid α(2,3) Galactose (7)	EY Laboratories	R-6802-1	1 mg/ml (stock

conjugate / (MAA-FITC)				solution) / 1:100 for LFC

SUPPL. TABLE S6 related to Fig. 2*D*: **Deep-sequencing-based transcriptomics for comparison of gene expression of ES03, CBiPSC2 and PMM2-iPSC-C3.**

"id" depicts the nomenclature according to HGNC (HUGO Gene Nomenclature Committee, http://www.genenames.org/). Transcript amounts for the cell lines ES03, CBiPSC2, PMM2-iPSC-C3 are presented in the respective columns. "Fold Change" is calculated as quotient of transcripts amounts of the indicated cell lines. If no transcript was detected for one of the two cell lines compares, "Fold Change" is either "Inf" or "NA". Pools of three biological replicates for each, ES03, CBiPSC2 and PMM2-iPSC-C3 were analyzed once. The "p-Value" reflects the technical significance of the analysis. Data are in a separate Excel file: SUPPL_TABLE_S6_deep-sequencing.

SUPPL. TABLE S7 related to Fig. 5: **Structures and relative intra-sample intensities of N-glycans identified in ES03, CBiPSC2, PMM2-iPSC-C3 and PMM2-iPSC-C3 transduced with control (shRenilla) and knock-down vector (shPMM2-2).**

**N-glycan Structure** 1)	**% Intensity** 2)				
	**ES03**	**CBiPSC2**	**PMM2-iPSC-3**	**shRenilla**	**shPMM2-2**
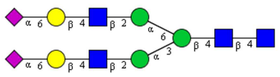	1.11 (±0.31)	1.07 (±0.30)	0.90 (±0.13)	1.22 (±0.04)	1.13 (±0.02)
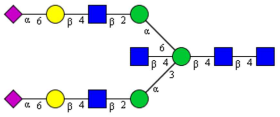	0.20 (±0.13)	0.35 (±0.17)	0.23 (±0.06)	0.19 (±0.06)	0.17 (±0.09)
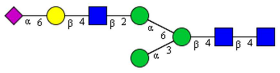	NQ	NQ	NQ	NQ	NQ
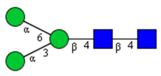	0.39 (±0.30)	0.81 (±0.29)	0.51 (±0.18)	0.99 (±0.03)	1.11 (±0.34)

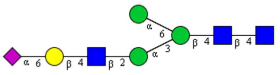	NQ	NQ	NQ	NQ	NQ
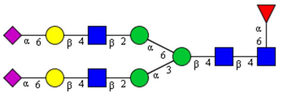	4.78 (±1.30)	6.01 (±0.80)	4.57 (±0.70)	4.91 (±0.26)	4.44 (±0.48)
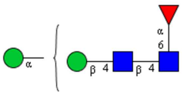	NQ	NQ	NQ	NQ	NQ
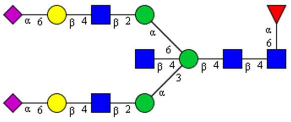	2.87 (±2.64)	2.76 (±0.93)	1.39 (±0.30)	1.75 (±0.23)	1.73 (±0.10)
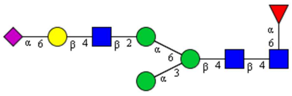	NQ	NQ	NQ	NQ	NQ
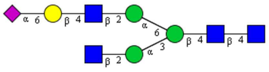 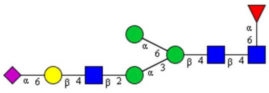	0.93 (±0.29)	0.96 (±1.07)	0.72 (±0.09)	0.80 (±0.06)	0.72 (±0.13)
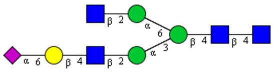	1.17 (±0.54)	0.54 (±0.31)	0.57 (±0.21)	0.80 (±0.20)	0.97 (±0.39)
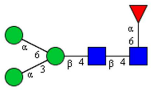 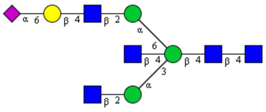	1.24 (±0.46)	0.82 (±0.34)	0.81 (±0.29)	1.92 (±0.33)	2.07 (±0.51)
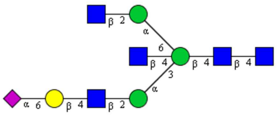	0.25 (±0.15)	0.26 (±0.14)	0.15 (±0.10)	0.33 (±0.08)	0.60 (±0.42)
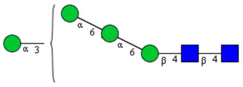	0.43 (±0.27)	0.20 (±0.21)	0.24 (±0.09)	0.21 (±0.12)	0.18 (±0.10)
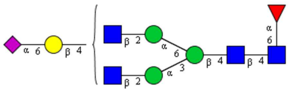	0.46 (±0.23)	0.41 (±0.18)	0.24 (±0.11)	0.35 (±0.10)	0.39 (±0.08)
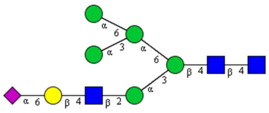	1.70 (±0.15)	1.48 (±0.28)	1.16 (±0.23)	1.29 (±0.06)	1.23 (±0.06)

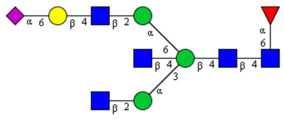	0.55 (±0.25)	0.68 (±0.17)	0.29 (±0.15)	0.34 (±0.05)	0.40 (±0.06)
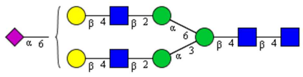 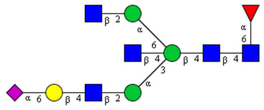	1.72 (±0.19)	1.45 (±0.24)	1.12 (±0.06)	0.98 (±0.07)	0.90 (±0.10)
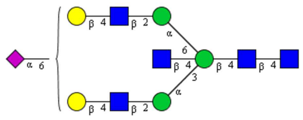	1.78 (±1.54)	0.95 (±0.38)	0.69 (±0.08)	0.62 (±0.04)	0.66 (±0.10)
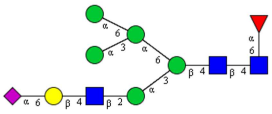 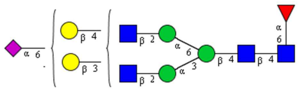	1.71 (±0.30)	1.57 (±0.39)	1.41 (±0.15)	1.19 (±0.21)	1.50 (±0.17)
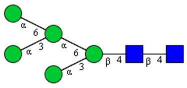 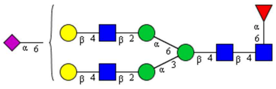 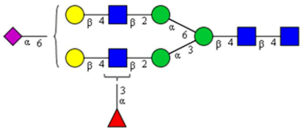	8.47 (±1.74)	7.04 (±0.30)	6.05 (±0.69)	5.07 (±0.83)	5.03 (±0.54)
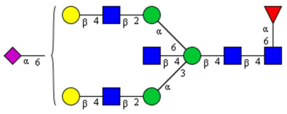	3.08 (±0.68)	0.74 (±0.29)	0.97 (±0.21)	0.79 (±0.13)	0.80 (±0.19)
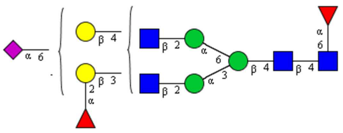	2.10 (±0.30)	2.43 (±0.65)	3.20 (±0.45)	3.67 (±0.64)	3.23 (±0.26)
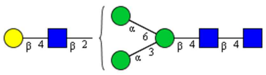	NQ	NQ	NQ	NQ	NQ
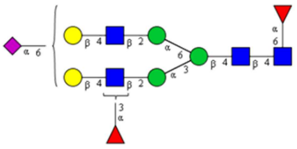	4.52 (±1.15)	9.40 (±1.31)	7.12 (±0.84)	7.00 (±0.39)	6.46 (±0.48)

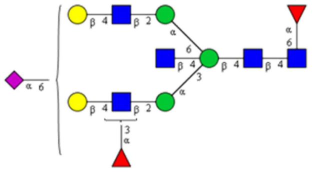<br>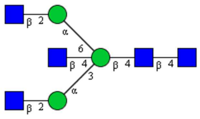	1.37<br>(±0.46)	0.82<br>(±0.23)	1.21<br>(±0.45)	1.40<br>(±0.27)	1.47<br>(±0.19)
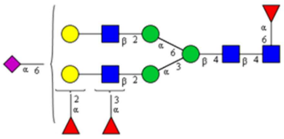	0.84<br>(±0.16)	1.31<br>(±0.29)	2.11<br>(±0.55)	1.64<br>(±0.35)	1.22<br>(±0.35)
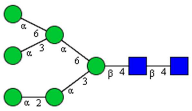	6.15<br>(±0.97)	7.24<br>(±1.35)	7.50<br>(±0.58)	8.49<br>(±1.23)	8.82<br>(±1.09)
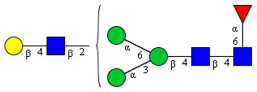<br>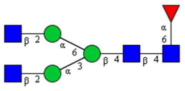	1.93<br>(±0.26)	1.33<br>(±0.48)	1.42<br>(±0.37)	1.29<br>(±0.12)	1.39<br>(±0.27)
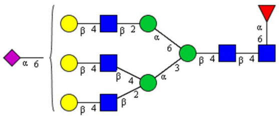	1.08<br>(±0.45)	0.32<br>(±0.26)	0.46<br>(±0.21)	0.13<br>(±0.04)	0.16<br>(±0.03)
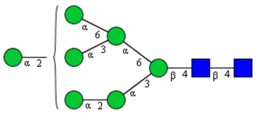	0.71<br>(±0.15)	0.36<br>(±0.12)	0.21<br>(±0.13)	0.19<br>(±0.08)	0.23<br>(±0.04)
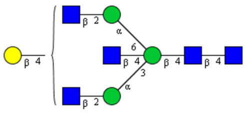	1.19<br>(±0.78)	NQ	0.48<br>(±0.22)	0.37<br>(±0.15)	0.71<br>(±0.31)
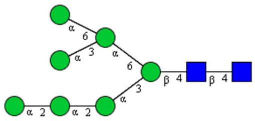	2.33<br>(±0.25)	2.95<br>(±0.20)	2.95<br>(±0.12)	3.01<br>(±0.29)	3.01<br>(±0.28)
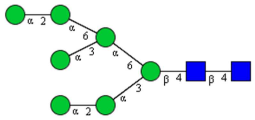	5.15<br>(±0.48)	4.61<br>(±1.42)	6.52<br>(±0.72)	5.00<br>(±0.52)	5.12<br>(±0.36)
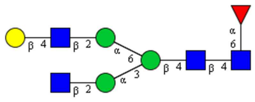	0.94	0.99	0.95	1.58	1.38

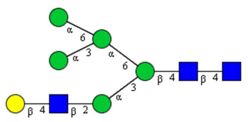	(±0.15)	(±0.33)	(±0.24)	(±0.31)	(±0.07)
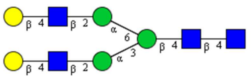<br>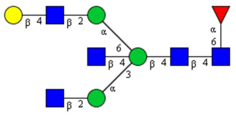	0.46 (±0.20)	0.42 (±0.20)	0.43 (±0.17)	0.73 (±0.18)	0.67 (±0.15)
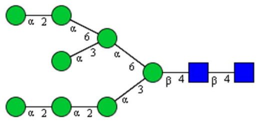	11.85 (±1.92)	13.20 (±1.34)	13.85 (±0.91)	13.88 (±0.36)	14.12 (±1.23)
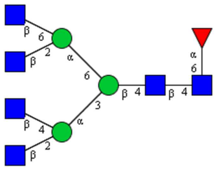<br>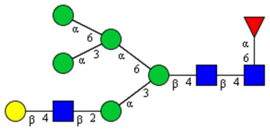	0.09 (±0.15)	0.18 (±0.08)	0.19 (±0.07)	0.61 (±0.18)	0.50 (±0.06)
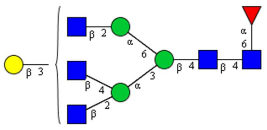<br>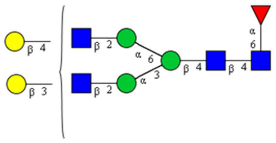	0.63 (±0.20)	0.58 (±0.41)	0.61 (±0.17)	0.38 (±0.07)	0.34 (±0.18)
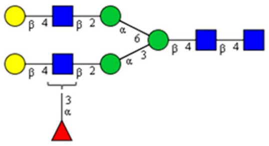	0.76 (±0.53)	0.20 (±0.09)	0.27 (±0.01)	0.16 (±0.05)	0.37 (±0.30)
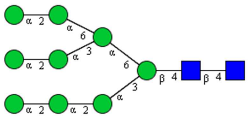<br>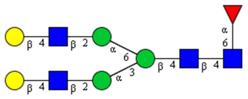	14.10 (±0.89)	16.18 (±1.38)	17.56 (±2.05)	15.30 (±1.27)	15.32 (±2.59)

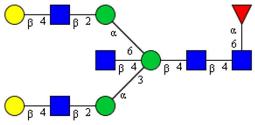	2.18 (±0.66)	0.68 (±0.23)	0.98 (±0.55)	0.58 (±0.09)	0.61 (±0.09)
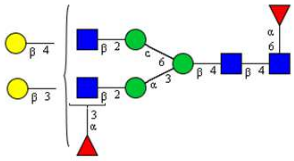	0.37 (±0.05)	0.61 (±0.40)	0.29 (±0.10)	0.41 (±0.07)	0.42 (±0.02)
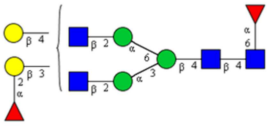<br>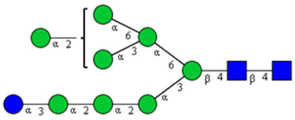	0.42 (±0.12)	0.46 (±0.06)	0.60 (±0.25)	0.52 (±0.02)	0.62 (±0.09)
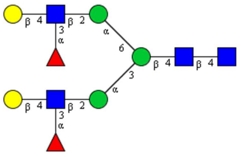	NQ	NQ	NQ	NQ	NQ
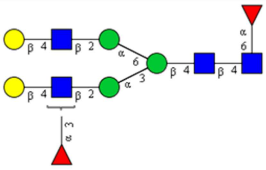	0.72 (±0.57)	1.42 (±1.24)	1.00 (±0.62)	0.47 (±0.03)	0.49 (±0.05)
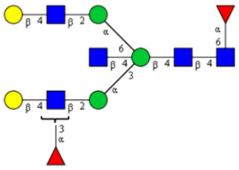	1.28 (±0.39)	0.83 (±0.35)	1.44 (±0.39)	1.24 (±0.17)	1.15 (±0.06)
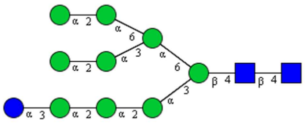<br>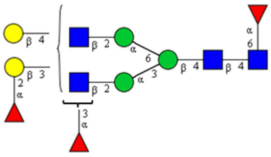	1.84 (±0.31)	2.07 (±0.29)	2.45 (±0.23)	2.33 (±0.22)	2.51 (±0.34)
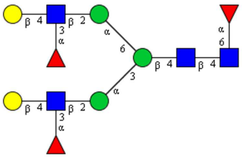	NQ	NQ	NQ	NQ	NQ

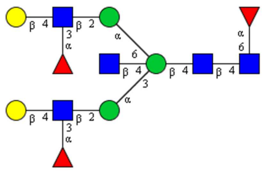	0.46 (±0.18)	0.85 (±0.22)	0.84 (±0.34)	1.36 (±0.30)	1.38 (±0.42)
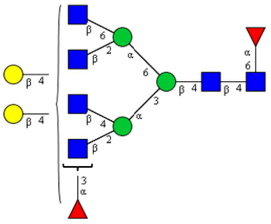	0.67 (±0.15)	0.44 (±0.15)	0.73 (±0.12)	0.51 (±0.17)	0.43 (±0.20)
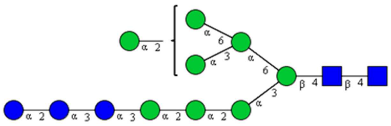	1.25 (±1.09)	0.67 (±0.39)	0.70 (±0.39)	1.18 (±0.17)	1.18 (±0.06)
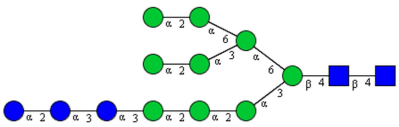	0.90 (±0.55)	0.77 (±0.37)	0.79 (±0.49)	1.20 (±0.11)	1.15 (±0.03)

1) 13 high-mannose-type and 48 complex (including some hybrid-type, isomers were counted as one structure) N-glycans that could be confirmed by exoglycosidase digest. Symbolic representation of N-glycan structures is drawn with GlycoWorkbench v.1.0.3353 (1) by the guidelines of the Consortium for Functional Glycomics (2). Green circle: mannose; yellow circle: galactose; blue circle: glucose; blue quadratic rectangle: N-acetylglucosamine; violet diamonds: sialic acid; red triangle: fucose. If the linkage is not written, then it is unknown.
2) Intensities of 49 quantifiable peaks were summed up for each cell line (total peak intensity = 100%) and relative peak intensities were calculated as % of total peak intensity. 300 µg of each sample was loaded and only peaks with a signal to noise ratio S/N ≥ 9 were selected for quantification. Peaks with 9 > S/N > 3 were defined as not quantifiable (NQ). Peaks with S/N ≤ 3 were not annotated. Values are average of three experiments with standard deviation.

SUPPL. MOVIE S1 related to Fig. 2: **Differentiation of PMM2-iPSC-C3 into cardiomyocytes.** Light microscopy movie of PMM2-iPSC-C3 upon cardiomyogenic differentiation at 4-fold magnification showing contractions characteristic for cardiomyocytes. Movie is in a separate file: SUPPL_MOVIE_S1

Reference List

1. Ceroni, A., Maass, K., Geyer, H., Geyer, R., Dell, A., and Haslam, S. M. (2008) GlycoWorkbench: a tool for the computer-assisted annotation of mass spectra of glycans. *J. Proteome. Res.* 7, 1650-1659

2. Varki, A., Cummings, R. D., Esko, J. D., Freeze, H. H., Stanley, P., Marth, J. D., Bertozzi, C. R., Hart, G. W., and Etzler, M. E. (2009) Symbol nomenclature for glycan representation. *Proteomics.* 9, 5398-5399

3. Fellmann, C., Hoffmann, T., Sridhar, V., Hopfgartner, B., Muhar, M., Roth, M., Lai, D. Y., Barbosa, I. A., Kwon, J. S., Guan, Y., Sinha, N., and Zuber, J. (2013) An optimized microRNA backbone for effective single-copy RNAi. *Cell Rep.* 5, 1704-1713

4. Shibuya, N., Goldstein, I. J., Van Damme, E. J., and Peumans, W. J. (1988) Binding properties of a mannose-specific lectin from the snowdrop (Galanthus nivalis) bulb. *J. Biol. Chem.* 263, 728-734

5. Baenziger, J. U., and Fiete, D. (1979) Structural determinants of concanavalin A specificity for oligosaccharides. *J. Biol. Chem.* 254, 2400-2407

6. Broekaert, W. F., Nsimba-Lubaki, M., Peeters, B., and Peumans, W. J. (1984) A lectin from elder (Sambucus nigra L.) bark. *Biochem. J.* 221, 163-169

7. Wang, W. C., and Cummings, R. D. (1988) The immobilized leukoagglutinin from the seeds of Maackia amurensis binds with high affinity to complex-type Asn-linked oligosaccharides containing terminal sialic acid-linked alpha-2,3 to penultimate galactose residues. *J. Biol. Chem.* 263, 4576-4585
